# Global Regulator SATB1 Recruits β-Catenin and Regulates T_H_2 Differentiation in Wnt-Dependent Manner

**DOI:** 10.1371/journal.pbio.1000296

**Published:** 2010-01-26

**Authors:** Dimple Notani, Kamalvishnu P. Gottimukkala, Ranveer S. Jayani, Amita S. Limaye, Madhujit V. Damle, Sameet Mehta, Prabhat Kumar Purbey, Jomon Joseph, Sanjeev Galande

**Affiliations:** 1 National Centre for Cell Science, Ganeshkhind, Pune, India; 2 Centre for Modelling and Simulation, University of Pune, Ganeshkhind, Pune, India; Stanford University School of Medicine, Howard Hughes Medical Institute, United States of America

## Abstract

In vertebrates, the conserved Wnt signalling cascade promotes the stabilization and nuclear accumulation of β-catenin, which then associates with the lymphoid enhancer factor/T cell factor proteins (LEF/TCFs) to activate target genes. Wnt/β -catenin signalling is essential for T cell development and differentiation. Here we show that special AT-rich binding protein 1 (SATB1), the T lineage-enriched chromatin organizer and global regulator, interacts with β-catenin and recruits it to SATB1's genomic binding sites. Gene expression profiling revealed that the genes repressed by SATB1 are upregulated upon Wnt signalling. Competition between SATB1 and TCF affects the transcription of TCF-regulated genes upon β-catenin signalling. GATA-3 is a T helper type 2 (T_H_2) specific transcription factor that regulates production of T_H_2 cytokines and functions as T_H_2 lineage determinant. SATB1 positively regulated *GATA-3* and siRNA-mediated knockdown of SATB1 downregulated *GATA-3* expression in differentiating human CD4^+^ T cells, suggesting that SATB1 influences T_H_2 lineage commitment by reprogramming gene expression. In the presence of Dickkopf 1 (Dkk1), an inhibitor of Wnt signalling, GATA-3 is downregulated and the expression of signature T_H_2 cytokines such as IL-4, IL-10, and IL-13 is reduced, indicating that Wnt signalling is essential for T_H_2 differentiation. Knockdown of β-catenin also produced similar results, confirming the role of Wnt/β-catenin signalling in T_H_2 differentiation. Furthermore, chromatin immunoprecipitation analysis revealed that SATB1 recruits β-catenin and p300 acetyltransferase on *GATA-3* promoter in differentiating T_H_2 cells in a Wnt-dependent manner. SATB1 coordinates T_H_2 lineage commitment by reprogramming gene expression. The SATB1:β-catenin complex activates a number of SATB1 regulated genes, and hence this study has potential to find novel Wnt responsive genes. These results demonstrate that SATB1 orchestrates T_H_2 lineage commitment by mediating Wnt/β-catenin signalling. This report identifies a new global transcription factor involved in β-catenin signalling that may play a major role in dictating the functional outcomes of this signalling pathway during development, differentiation, and tumorigenesis.

## Abstract 2

Chromatin organizer SATB1 and Wnt transducer β-catenin form a complex and regulate expression of GATA3 and T_H_2 cytokines in Wnt-dependent manner and orchestrate T_H_2 lineage commitment.

## Author Summary

In vertebrates the canonical Wnt signalling culminates in β-catenin moving into the nucleus where it activates transcription of target genes. Wnt/β-catenin signalling is essential for the thymic maturation and differentiation of naïve T cells. Here we show that SATB1, a T cell lineage-enriched chromatin organizer and global regulator, binds to β-catenin and recruits it to SATB1's genomic binding sites so that genes formerly repressed by SATB1 are upregulated by Wnt signalling. Some of the genes known to be regulated by SATB1 (such as genes encoding cytokines and the transcription factor GATA3) are required for differentiation of Th2 cells, an important subset of helper T cells. Specifically we show that siRNA-mediated knockdown of SATB1 downregulated *GATA-3* expression in differentiating human CD4^+^ T cells. Inhibiting Wnt signalling led to downregulation of GATA-3 and of signature T_H_2 cytokines such as IL-4, IL-10, and IL-13. Knockdown of β-catenin also produced similar results, thus together these data confirm the role of Wnt/β-catenin signalling in T_H_2 differentiation. Our data demonstrate that SATB1 orchestrates T_H_2 lineage commitment by modulating Wnt/β-catenin signalling.

## Introduction

Wnt growth factors regulate a variety of developmental processes by altering specific gene expression patterns [1]. Wnt proteins are secreted molecules that coordinate cell-to-cell interactions in many different cell types by binding to a member of the Frizzled (Fz) family of transmembrane receptors [2]. Binding of Wnt to Fz (Table S1) elicits a complex cascade of molecular events culminating in the inhibition of the negative regulatory kinase GSK-3β [3]. Phosphorylation of β-catenin by GSK-3β targets it for degradation via the β-TrCP ubiquitin ligase-proteasome pathway [4]. Dephosphorylated β-catenin accumulates inside the nucleus [5] where it associates with the lymphoid enhancer factor/T cell factor (LEF/TCF) transcription factors to induce target gene transcription [6]. In vertebrates, β-catenin acts as a transcriptional activator, which is required to overcome the transcriptional repression by repressor complexes [7]. The C-terminus of β-catenin is indispensable for the transactivation function, presumably since it harbours binding sites for transcriptional coactivators such as p300/CBP and TBP [7],[8]. Thus, recruitment of chromatin remodelling factors on TCF's genomic targets to modulate the gene transcription appears to be the principal function of stabilized β-catenin [8].

Within the thymus, thymocyte maturation involves a series of discrete phenotypic stages that correspond to developmental checkpoints and are subsequently referred to as CD4^−^CD8^−^ (DN), CD4^+^CD8^+^ (DP), and CD4^+^CD3^+^ or CD8^+^CD3^+^ (SP). In addition to the well-studied T cell receptor (TCR)-mediated signals and the Notch pathway, thymic epithelial cells also contribute towards thymocyte development and differentiation by producing Wnt [2],[9]. The role of Wnt signalling in T cell development is highlighted by the fact that soluble Fz receptors have been shown to block early thymocyte development in fetal thymic organ culture at the DN-to-DP transition [10]. Furthermore, ablation of β-catenin from T cells resulted in poor β-selection and the DN stage of T cell development was impaired [11]. The SP cells then further differentiate into various functional T cell populations such as the T helper (T_H_) cells. Upon antigenic stimulus, naïve CD4^+^ T_H_ cells differentiate into distinct effector cell lineages referred to as T_H_1 and T_H_2. This differentiation is mediated by signalling proteins such as STAT4 and STAT6 and is accompanied by upregulation of marker transcription factors such as GATA-3 and c-Maf in T_H_2 cells and T-bet in T_H_1 cells [12]. The transcription factors GATA-3 and T-bet dictate differentiation of CD4^+^ T cells into T_H_2 and T_H_1 cell types, respectively [13],[14]. However, although the contributions of various transcription factors and signalling pathways towards the T_H_ cell differentiation have been studied extensively, there is no report demonstrating the direct role of Wnt signalling in T_H_ cell differentiation.

The T-lineage-enriched chromatin organizer special AT-rich sequence binding protein 1 (SATB1) was shown to regulate distant genes by selectively tethering matrix attachment regions (MARs) to the nuclear matrix [15]. Furthermore, SATB1 acts as a “docking site” for several chromatin modifiers, including ACF, ISWI, and HDAC1 [16],[17], and these chromatin modifiers were suggested to suppress gene expression through histone deacetylation and nucleosome remodelling at SATB1-bound MARs [16]. SATB1 organizes the T helper 2 (T_H_2) cytokine and MHC class-I loci into distinct chromatin loops by tethering MARs to the nuclear matrix at fixed distances [18],[19]. The densely looped and transcriptionally active chromatin structure organized by SATB1 is essential for coordinated expression of the T_H_2 cytokine genes [18]. Moreover, SATB1 seems to play a role in dynamic organization of the transcriptionally poised chromatin [20]. SATB1 also regulates gene expression by recruiting various chromatin modifiers to promoters [21]. Interaction between SATB1 and partner proteins is frequently mediated by its N-terminal PDZ-like domain, which is also important for SATB1 homodimerization [22],[23]. Additionally, SATB1 possesses a MAR-binding domain in its C-terminal half comprising a cut domain (CD) and a homeodomain (HD) that together contribute towards recognition and high affinity binding of MARs [23],[24]. SATB1 regulates a large number of genes involved in T cell proliferation, development, and differentiation [21],[25]. SATB1 itself is differentially expressed in various subsets of T_H_ cells [26], however the role of SATB1 in their differentiation has not been demonstrated. Interestingly, many of SATB1's target genes such as *c-Myc* [15] and *Bcl-2* [27] are also targeted by Wnt/β-catenin [28], suggesting a functional overlap between Wnt/β-catenin pathway and SATB1.

In this study, we set out to unravel the molecular mechanisms contributing towards the functional overlap between SATB1 and β-catenin pathways, especially with respect to their target gene regulation. We show that SATB1 physically interacts with β-catenin and recruits it to its genomic targets. Interestingly, deacetylation of SATB1 upon Wnt/β-catenin signalling leads to an increase in its occupancy on genomic targets. Recruitment of β-catenin alters the transcription of SATB1's target genes in thymocytes. Chromatin immunoprecipitation (ChIP) analysis of SATB1 binding sites (SBSs) in promoters of multiple genes revealed that interaction with β-catenin modulated SATB1 function on its target genes by increasing its occupancy and altering histone H3 lysine 9 (H3K9) acetylation. β-catenin-responsive genes are also targeted by SATB1, suggesting that both are functionally linked in the Wnt/β-catenin signalling pathway. Additionally, β-catenin-induced TCF responsive genes are dysregulated upon SATB1 overexpression, suggesting competitive interaction of TCF/LEF and SATB1 with β-catenin. Furthermore, SATB1 directly regulates GATA-3 expression and dictates T_H_2 lineage commitment via Wnt/β-catenin signalling. As a functional consequence of abrogating function of SATB1 or β-catenin or Wnt signalling, we demonstrate that the production of signature T_H_2 cytokines such as IL-4, IL-10, and IL-13 is reduced, indicating that T_H_2 differentiation is affected. Thus, these studies establish SATB1 as a determinant of T_H_2 differentiation and downstream effector in the Wnt/β-catenin signalling pathway.

## Results

### SATB1 Interacts with C-Terminal of β-Catenin via Its N-Terminal PDZ-Like Domain

**Figure 1:**
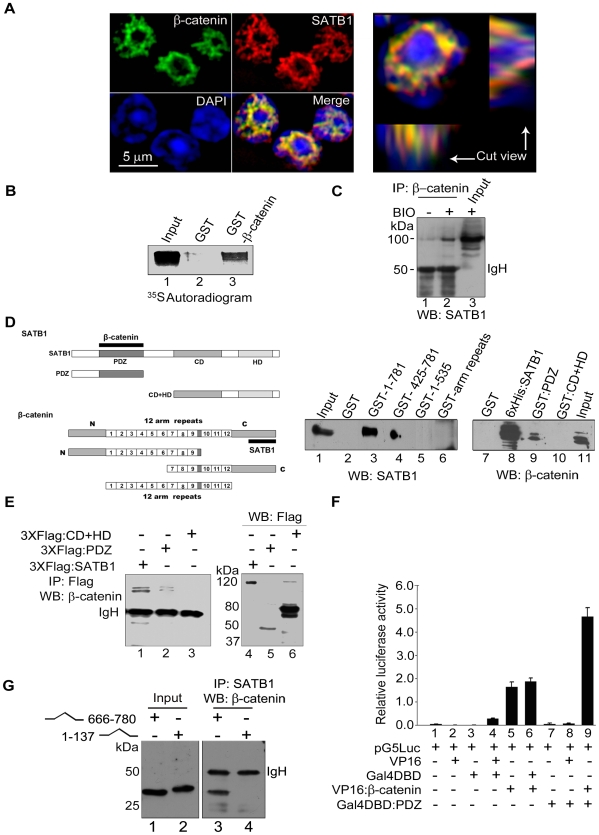
Delineation of physical interaction between SATB1 and β-catenin. (A) SATB1 and β-catenin colocalize in the thymocyte nuclei. Indirect immunofluorescence staining of thymocytes using antibodies to SATB1 (red) and β-catenin (green) was performed as described in Materials and Methods. DNA counterstaining was performed using DAPI (blue). The cut view panel depicts two perpendicular transverse sections of a triple-stained thymocyte as indicated by white lines, intersecting at the point of the brightest fluorescence signal. (B) Direct interaction between SATB1 and β-catenin was monitored by in vitro pulldown assays performed as described in Materials and Methods. ^35^S-labeled SATB1 was specifically pulled down after incubation with immobilized GST-β-catenin (lane 3) and not with control immobilized GST (lane 2). (C) In vivo interaction of SATB1 and β-catenin was assessed by performing coimmunoprecipitation analysis as described in Materials and Methods. Nuclear extracts derived from BIO treated (+) and control (−) human thymocytes were immunoprecipitated using anti-β-catenin followed by WB with anti-SATB1. (D) The interacting regions of SATB1 and β-catenin were mapped by in vitro pulldown assay. GST pulldowns of SATB1 and β-catenin were performed by passing Jurkat nuclear extract on immobilized domains of GST-β-catenin (lanes 1–6) and SW480 nuclear extract on immobilized domains of SATB1 (lanes 7–11) including both full-length proteins followed by WB with anti-SATB1 and anti-β-catenin. SATB1 and β-catenin truncations used are depicted schematically on the left. Solid black bars depict the respective interacting regions. (E) Coimmunoprecipitation analysis of extracts derived from HEK 293T cells overexpressing 3XFlag-SATB1 and its functional domains using anti-Flag antibody followed by WB with anti-β-catenin (lanes 1–3). Expression levels of the 3XFlag-fused domains of SATB1 were monitored by WB using anti-Flag (lanes 4–6). (F) Mammalian two hybrid assay was performed to score for protein-protein interactions in HEK 293T cells essentially as described [23]. The C-terminus of β-catenin and the PDZ-like domain of SATB1 were expressed as fusions with VP-16 and GAL4-DBD using the pACT and pBIND vectors of the CheckMate mammalian two-hybrid system (Promega). pBIND and pACT fusion constructs were transfected along with a reporter vector pG5-Luc containing 4× GAL4 responsive element and luciferase activity was compared with the control. Error bars represent standard deviation calculated from triplicates. (G) The C-terminus and not the N-terminus of β-catenin is involved in its interaction with SATB1. VP-16 fused C-terminal (aa 666–780, lane 1) and N-terminal (aa 1–137, lane 2) regions of β-catenin were overexpressed in HEK 293T cells. Co-immunoprecipitation was performed as described in Materials and Methods using anti-SATB1 followed by WB using anti-β-catenin.

Gene expression profiles of cells expressing phosphorylation- or acetylation-defective mutants of SATB1 [21] indicated shared target genes with the β-catenin signalling pathway (Figure S1). SATB1 is expressed abundantly in thymocytes [15], and therefore we monitored the subcellular localization of β-catenin and SATB1 in thymocytes upon Wnt induction. Surprisingly, the intranuclear immunostaining pattern of β-catenin also resembled the “cage-like” architecture of SATB1-containing nuclear domains in thymocytes (Figure 1A, Videos S1 and S2) [15]. Optical sectioning revealed that at least part of these signals colocalized across the depth of the nucleus, indicating that they occupy similar areas within the thymocyte nucleus (Figure 1A). To test whether SATB1 and β-catenin interact physically, we performed in vitro pulldowns using immobilized β-catenin. When SATB1 was passed on GST-β-catenin and GST immobilized on Sepharose beads, SATB1 eluted specifically from the GST-β-catenin affinity matrix, suggesting their physical interaction (Figure 1B). Coimmunoprecipitation analysis using nuclear extract from Jurkat T lymphoblastic cells indicated that β-catenin and SATB1 can be immunoprecipitated by antibodies against each other (Figure S2). We then tested the effect of Wnt signalling on the physical association of SATB1 and β-catenin. LiCl and 6-bromoindirubin-3′-oxime (BIO) are potent GSK-3β inhibitors that mimic Wnt signalling by stabilizing β-catenin [29]. Upon BIO treatment, increased interaction was observed in human thymocytes (Figure 1C). In vitro pulldown experiments using the various truncated and tagged proteins (Figure S3) indicated that the PDZ-like domain of SATB1 and carboxyl (C) terminal region of β-catenin (577–781 amino acids) are indispensable for this interaction (Figure 1D). Additionally, in vivo co-immunoprecipitation of β-catenin with Flag:PDZ (1–204 aa) but not with Flag:CD+HD (330–763 aa) indicated that β-catenin interacts with the PDZ-like domain of SATB1 (Figure 1E). This interaction is very specific since expression of Flag-PDZ is at least 5-fold less as compared to that of Flag:CD+HD (Figure 1E, compare lanes 5 and 6). Finally, luciferase reporter based mammalian two-hybrid assay further independently confirmed that the PDZ-like domain of SATB1 is involved in its interaction with β-catenin in vivo. Although overexpression of the C-terminal region of β-catenin itself led to transactivation (Figure 1F, bar 5), along with the PDZ-like domain the reporter activity is enhanced further (bar 9), suggesting their functional association. To further assess the involvement of the C- terminal regions of β-catenin towards its interaction with SATB1, we generated deletion constructs of β-catenin where N-terminus harbouring truncation consisted of aa 1–137 and C-terminus that of aa 666–781. GST pulldown assay revealed that the C-terminal region encompassing aa 577–781 of β-catenin is important for its interaction with SATB1, and immunoprecipitation data using 1–137 aa and 666–780 aa regions also confirmed the importance of extreme C-terminus (666–781 aa) in this interaction (Figure 1G). Collectively, these results indicate that the N-terminal PDZ-like domain of SATB1 interacts with the C-terminal region of β-catenin and that this interaction occurs in the nucleus.

### Recruitment of β-Catenin upon Wnt Signalling Alters the Transcription of SATB1 and Wnt Target Genes

**Figure 2:**
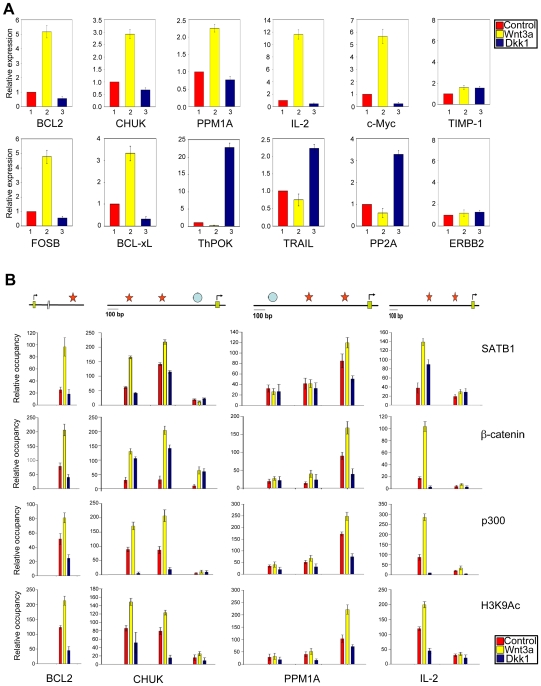
Wnt signalling results in upregulation of SATB1 targeted genes by recruitment of β-catenin-p300 complex. (A) Effect of Wnt signalling on the transcription status of representative SATB1 regulated genes (Upper row) and Wnt regulated genes (lower row) in thymocytes. Quantitative RT-PCR analysis was performed using RNA extracted from control human thymocytes (bar 1) and thymocytes treated for 48 h with Wnt3a (bar 2), or Dkk1 (bar 3) as described in Materials and Methods. The values for gene expression in treated cells were normalized with respect to the untreated control, which was set to 1. Each error bar indicates standard deviation calculated from triplicates. *TIMP-1* and *ERBB2* served as control genes such that TIMP-1 is not regulated by both SATB1 and β-catenin, whereas ERBB2 served as SATB1-dependent [49] but β-catenin-independent control gene. (B) Occupancy of SATB1, β-catenin, p300 acetyltransferase, and H3K9 acetylation across the 1 kb upstream regulatory regions of SATB1 regulated genes *Bcl-2*, *PPM1A*, *CHUK*, and *IL-2* was monitored by ChIP analysis. Chromatin was isolated from control, Wnt3a, or Dkk1 treated human thymocytes and ChIP analysis was performed as described in Materials and Methods. Relative occupancy was calculated by performing quantitative real-time PCR analysis and normalizing the *C*_T_ values with input and IgG controls. Each error bar indicates standard deviation calculated from triplicates. The relative positions of regions analyzed by ChIP within respective genes are schematically indicated above their occupancy profiles. Stars represent in vitro SBSs whereas circles denote non-binding sites. Names of genes are depicted below each column of graphs, whereas that of antibodies used for ChIP are depicted on the right side of each row.

To investigate whether SATB1 and β-catenin collaborate functionally, we monitored the effect of SATB1 and β-catenin interaction on SATB1-mediated gene expression. For in vivo reporter assay, we used a construct in which the well-characterized SATB1 binding MAR from the IgH locus is cloned into a promoterless vector [30]. Overexpression of β-catenin enhanced the IgH MAR-driven luciferase reporter activity in the wild-type but not the mutant IgH MAR, suggesting that this effect is mediated by SATB1 (Figure S4). The T41A mutant of β-catenin used in reporter studies cannot be phosphorylated by GSK-3β [31] and is therefore constitutively stabilized and activated. We next monitored the effect of SATB1:β-catenin interaction on the transcription of multiple genes that are known to be targets of SATB1 [21] as well as Wnt signalling in immature T cells [32]. Wnt signalling was induced in human thymocytes by treating them with soluble Wnt3a ligand for 48 h, and the transcriptional activity of SATB1 targets was monitored by quantitative RT-PCR. In prolonged suspended cultures, thymocytes are known to undergo cell death. However, during the course of these treatments over 48 h in culture, thymocyte viability was not significantly reduced even without using the OP9-DL1 co-culture system (Figure S5A). The transcription status of multiple Wnt- and SATB1-responsive genes was reversed upon induction of Wnt signalling by addition of Wnt3a in thymocyte culture (Figure 2A, bar 2). Thus, genes that are downregulated by SATB1 such as *BCL-2* [27], *CHUK*, *PPM1A*, *c-Myc*, and *IL-2* [21] were upregulated upon overexpression of β-catenin or addition of Wnt3a in a manner akin to the known Wnt targets *FOSB* and *BCL-X_L_* [32], suggesting that these are also β-catenin targets. Furthermore, inhibition of Wnt signalling by Dickkopf 1 (Dkk1) treatment led to transcriptional repression of all SATB1 regulated genes that are also targeted by Wnt signalling (Figure 2A, bar 3). Among the SATB1 regulated genes, *ThPOK* (ZBTB1-7B) showed a reciprocal pattern of regulation. ThPOK was downregulated upon Wnt3a treatment and dramatically upregulated (more than 20-fold) upon inhibition of Wnt signalling by Dkk1. Among the known Wnt targets in thymocytes, few genes such as *TRAIL* and *PP2A* [32] were downregulated upon Wnt3a treatment and upregulated upon Dkk1 treatment. More importantly, *TIMP1* that is neither targeted by SATB1 nor targeted by β-catenin is not affected by Wnt3a or Dkk1 treatment of thymocytes, indicating that this effect is specific to SATB1 and Wnt/β-catenin targets (Figure 2A). *ERBB2* is a target of SATB1 but not that of β-catenin and therefore is not affected upon Wnt3a treatment, further demonstrating that to overcome the SATB1 mediated repression, SATB1:β-catenin functional interaction is essential to SATB1 genomic targets (Figure 2A). Gene expression analysis using thymocytes co-cultured with OP9-DL1 cells supplemented with IL-7 and Flt3 ligand revealed that transcription of Wnt targets PP2A and *BCL-X_L_* is not significantly different than those cultured on the control OP9 cells (Figure S5B). These results indicated that under the culture conditions employed, the effects on gene expression are not due to differential effects of these treatments on thymocyte viability. Thus, interaction with β-catenin dramatically alters the transcription status of SATB1 regulated genes, many of which are also known Wnt targets. To study how SATB1 directly regulates expression of these genes, we performed in vitro binding assays to assess the binding status of SATB1 on various regulatory regions from these genes and observed that SATB1 binds to at least one site located within the upstream 1 kb regions of multiple genes (Figure S6). To confirm whether the upregulation of transcription is mediated by SATB1 and factors recruited by SATB1, we performed ChIP analysis of promoters of these genes. Chromatin was isolated from untreated control, Wnt3a or Dkk1 treated human thymocytes and subjected to immunoprecipitation using antibodies to SATB1, β-catenin, p300, and H3K9 acetylation (H3K9Ac). The DNA purified from immunoprecipitate was subjected to PCR amplification using oligonucleotide primers flanking various regions within the upstream 1 kb regulatory regions of *CHUK*, *PPM1A*, and *IL-2* promoters and the SBS within the major breakpoint region of *BCL2* [27]. ChIP analysis revealed that not all of the in vitro binding sites were occupied by SATB1 in vivo (Figure 2B). Strikingly, upon Wnt3a treatment the occupancy of SATB1 was enriched by 1.5- to 4-fold on promoters of genes that are downregulated by SATB1. This change in occupancy is an outcome of Wnt signalling since treatment with Dkk1 resulted in a 1.5- to 6-fold decrease in SATB1 occupancy on its in vivo binding sites (Figure 2B). The changes in occupancy of β-catenin, p300, and H3K9Ac also mirrored that of SATB1, suggesting that SATB1 occupancy is the primary event leading to recruitment of β-catenin and p300 (Figure 2B). These changes in occupancy were highly specific and restricted to SBSs since the regions in proximal *CHUK* and distal *PPM1A* promoters that were not bound by SATB1 in vitro and in vivo did not show any significant change in the occupancy of SATB1, β-catenin, and p300 (Figure 2B). Thus, Wnt signalling results in increased occupancy of SATB1 on its targets, which then recruits β-catenin and p300 to upregulate target genes.

### Direct Interaction between SATB1 and β-Catenin Is Required for their Transcriptional Effects

**Figure 3:**
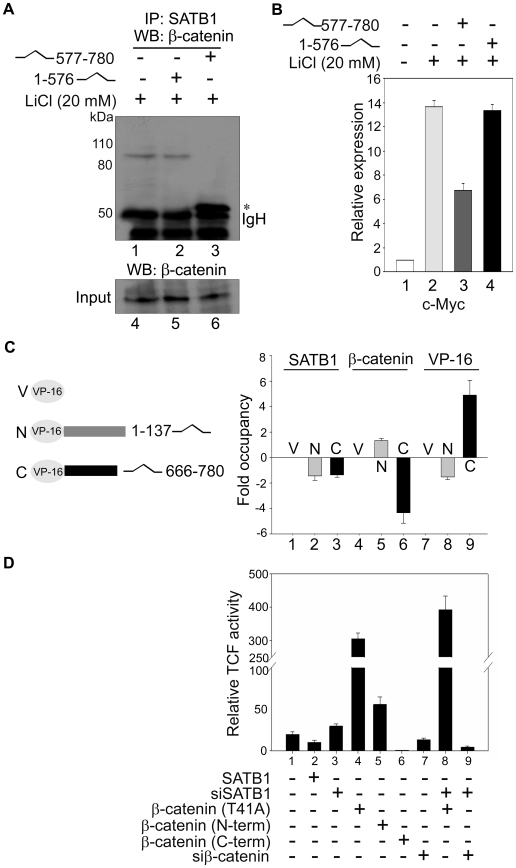
Direct interaction between SATB1 and β-catenin is essential for target gene regulation. (A) Overexpression of truncated β-catenin (577–780 aa) competitively overcomes association of SATB1 with endogenous full-length β-catenin. Co-immunoprecipitation was performed using extracts from LiCl-treated HEK 293T cells with anti-SATB1 followed by WB using anti-β-catenin as described in Materials and Methods. Asterisk indicates position of the immunoprecipitated truncated C-terminus of β-catenin, which migrates just above the immunoglobulin heavy chain (IgH). Lower panel depicts WB with input extracts used for co-immunoprecipitation. (B) Effect of SATB1–β-catenin interaction on the transcription status of the SATB1 regulated gene *c-Myc*. Quantitative RT-PCR analysis was performed as described in Materials and Methods using RNA extracted from HEK 293T cells without LiCl treatment (bar 1, normalized to unit value), with LiCl (bars 2–4), and transfected with C-term (577–780 aa) (bar 3) and N-term (1–576 aa) (bar 4) of β-catenin. Cells were cultured for 48 h after administering all these treatments prior to isolation of RNA. Each error bar depicts standard deviation calculated from triplicates. (C) Overexpression of truncated β-catenin C-terminus (666–780 aa) competitively overcomes association of endogenous full-length β-catenin on SATB1 genomic targets. HEK 293T cells were transfected with pACT vector encoding either the VP16 tag, VP16-fused N-term (1–137 aa), or C-term (666–780 aa) domains of β-catenin and treated with LiCl. After 24 h, cells were harvested and chromatin was isolated by sonication and subjected to ChIP assay using antibodies indicated above the bars. The antibody to β-catenin used in this assay was raised against its N-terminus. The expression vectors and constructs used are depicted schematically on the left. ChIP-PCRs were performed using oligonucleotide primers specific to *c-Myc*-SBS as described in Materials and Methods. Graph depicts fold enrichment of the ChIP-PCR products over the vector control (“V”) measured by quantitative real-time PCR analysis. Negative values indicate reduced occupancy, whereas positive values depict increased occupancy with respect to the vector control. Grey and black bars represent the fold changes in occupancy of SATB1 (bars 2–3), β-catenin (bars 5–6), and VP16 (bars 8–9) in LiCl treated HEK 293T cells overexpressing the N-term (“N”) or the C-term (“C”) domain truncations of β-catenin, respectively. (D) Wnt signalling activity was measured by performing transactivation assay in HEK 293T cells using the TOPFlash and FOPFlash reporter constructs and cotransfections of SATB1, β-catenin (T41A), siSATB1, si-β-catenin, β-catenin N-term (1–576 aa), and β-catenin C-term (577–780 aa) in indicated combinations. The T41A mutant of β-catenin used in reporter assays cannot be phosphorylated by GSK-3β and is therefore constitutively stabilized and activated. The reporter activity was measured after 48 h as described in Materials and Methods. The ratio of luciferase activities in TOPFlash-transfected versus FOPFlash-transfected cells was determined and plotted as the relative TCF activity. Each error bar indicates standard deviation calculated from triplicates.

To ascertain whether transcription regulation upon Wnt signalling is a direct consequence of the interaction between SATB1 and β-catenin or is an indirect effect of their interaction, we used the deletion constructs of β-catenin where C-terminus deleted truncation (ΔC-term) consisted of aa 1–576 and only C-terminus harbouring construct that of aa 577–780. We tested the possibility of whether overexpression of this interaction region works as a dominant negative for SATB1 and β-catenin interaction. Towards this, we overexpressed 1–576 and 577–780 regions of β-catenin as red fluorescent protein (RFP) fusions in Human Embryonic Kidney (HEK)-293T cells and induced β-catenin signalling by adding LiCl. SATB1 immunoprecipitation followed by immunoblot analysis with anti-β-catenin confirmed that they form a complex in LiCl-treated cells (Figure 3A, lane 1). However, upon overexpression of the C-terminus (577–780), endogenous β-catenin no longer associated with SATB1 as evidenced by a lack of signal in the coimmunoprecipitation assay (Figure 3A, lane 3). Overexpression of the ΔC-term (1–576) did not lead to the displacement of endogenous β-catenin (Figure 3A, lane 2). This result suggests that endogenous full-length β-catenin and overexpressed C-terminus of β-catenin compete with each other to bind with SATB1; hence the latter serves as a dominant negative for their association. Interestingly, quantitative RT-PCR analysis under such conditions yielded reduction in the activating effect of stabilized β-catenin on *c-Myc* that is known to be repressed by SATB1 [15]. However, overexpression of the ΔC-term β-catenin did not alter the expression profile of *c-Myc* (Figure 3B). Thus, overexpression of C-terminus alters the effect of β-catenin on transcription of SATB1 targets in vivo. To investigate the molecular mechanism of this effect, we tested the occupancy of SATB1 and β-catenin at the *c-Myc* promoter. ChIP analysis demonstrated the occupancy of both SATB1 and β-catenin specifically at the SBS of *c-Myc* promoter in vivo but not at another region 10 kb upstream of the start site (Figure S7). Next, we independently overexpressed VP16-fused truncations 1–137 and 666–780 of β-catenin representing its extreme N- and C-termini, respectively, in HEK 293T cells followed by treatment with LiCl for 24 h. Extreme terminal regions of β-catenin were overexpressed in this assay to avoid any interference from proteins interacting with the arm repeats such as TCF. ChIP analysis using SATB1 and β-catenin antibodies followed by quantitative ChIP PCRs revealed that overexpression of C-terminal region of β-catenin (666–780) led to the reduced occupancy of β-catenin on *c-Myc* SBS (Figure 3C, bar 6). In contrast, overexpression of N-terminal region of β-catenin (1–137) failed to exert the same effect (Figure 3C, bar 5). Since the epitope of anti-β-catenin used here spans only the N-terminal region, we used an antibody for the VP16 fusion tag to monitor whether the C-terminus is indeed recruited by SATB1. Quantitative ChIP PCRs yielded about 5-fold enrichment of the C-terminus of β-catenin at the *c-Myc* SBS (Figure 3C), providing compelling evidence for the in vivo displacement of full-length β-catenin. We next monitored transcription activity under the control of a concatemerized TCF binding site–driven reporter TOP-Flash and its non-TCF binding mutant version FOP-Flash [33]. Interestingly, although SATB1 does not directly bind to the consensus TCF binding site (Figure S8), overexpression of SATB1 led to repression of TCF reporter–driven transcription (Figure 3D, compare bars 1 and 2), indicating that SATB1 may compete with TCF for forming a complex with β-catenin. Strikingly, silencing of SATB1 upregulated TCF-driven transcription (bar 3), which was dramatically induced when β-catenin was simultaneously overexpressed, suggesting a role of SATB1 in titrating β-catenin in vivo (Figure 3D, bar 8). This increase in reporter activity was significantly more than with β-catenin alone (bar 4). We used the T41A constitutively active mutant of β-catenin for the TCF reporter assay. Next, we used the RFP-fused N-term (1–576) and C-term (577–780) regions of β-catenin as described above to test whether the Wnt-responsive reporter activity is a direct consequence of the interaction between SATB1 and β-catenin. Overexpression of the N-term (1–576) β-catenin that interacts with TCF but does not interact with SATB1 upregulated the TCF-reporter activity, although to a much lower extent (bar 5). The activating effect of this arm repeat containing region of β-catenin may be explained by its ability to sequester inhibitors such as ICAT that inhibit TCF-β-catenin interaction. However, upon overexpression of only the C-terminal region (577–780) of β-catenin that interacts with SATB1 but not with TCF, the reporter activity was completely abolished (bar 6), presumably due to the dominant negative effect exerted by the titration of endogenous p300. Such interaction may result in the diminished availability of CBP/p300 for association with the TCF:β-catenin complex on TCF responsive elements, resulting in the non-functional interaction between TCF and β-catenin. Alternatively, the pool of endogenous full-length β-catenin that is displaced from SATB1 upon overexpression of C-terminal of β-catenin may not be available for binding to TCF and the subsequent activation of target genes and may simply be turned over. Collectively, these findings provide direct evidence that SATB1-β-catenin interaction is required for mediating the Wnt-dependent effects on target gene expression.

### Competition between SATB1 and TCF Affects the Transcription of TCF-Regulated Genes upon β-Catenin Signalling

**Figure 4:**
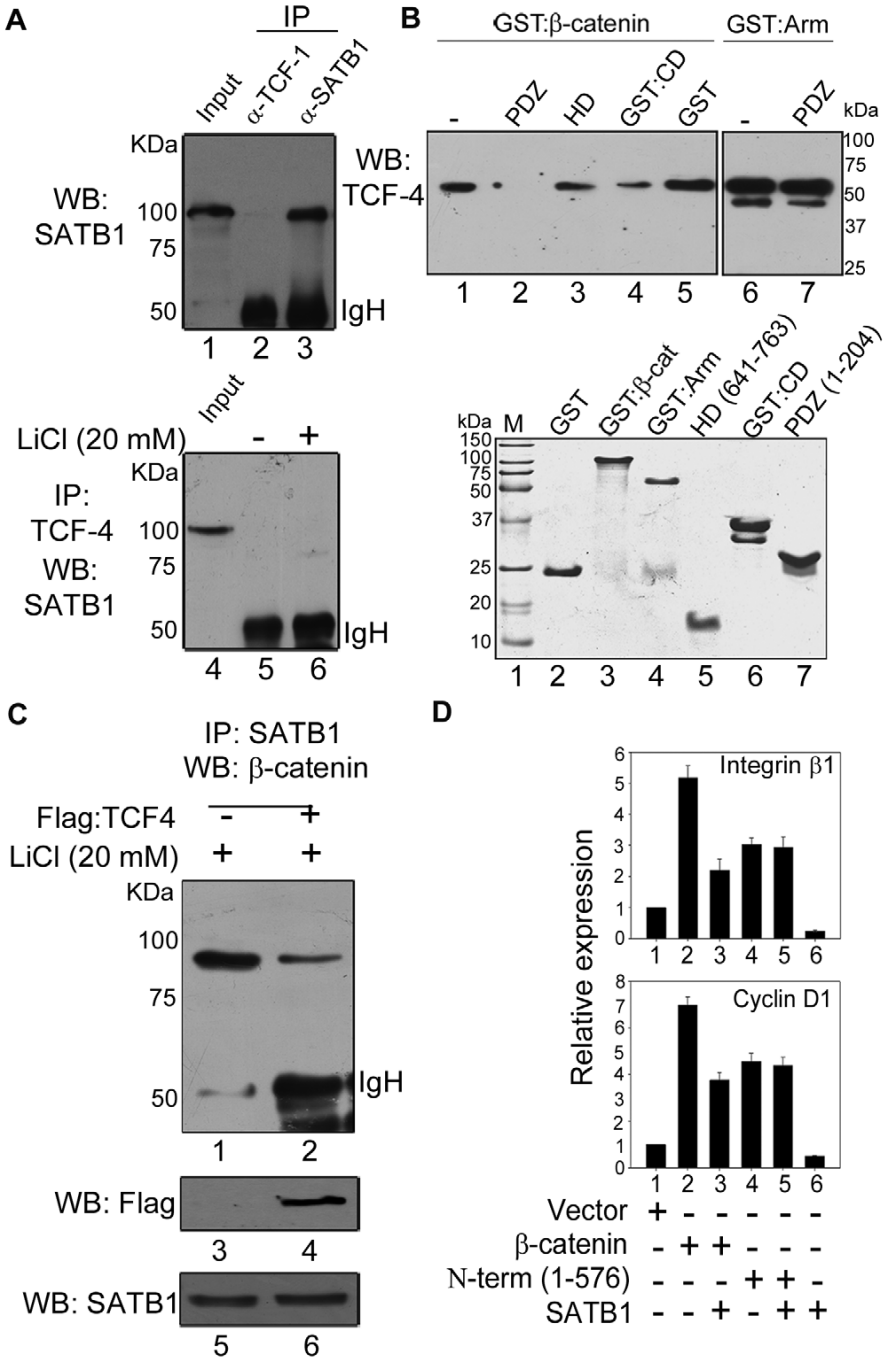
Competition between TCF and SATB1 for recruitment of β-catenin affects the TCF mediated transcription regulation. (A) SATB1 does not interact with TCF proteins. Immunoprecipitation was performed as described in Materials and Methods. Nuclear extracts derived from human thymocytes treated with LiCl treated for 24 h were immunoprecipitated using anti-TCF-1 (lane 2) and anti-SATB1 (lane 3) followed by immunoblot with anti-SATB1. Similarly, immunoprecipitation was performed by incubating anti-TCF-4 with nuclear extracts from HEK 293T cells cultured in the presence (lane 6) and absence (lane 5) of LiCl for 24 h followed by immunoblot with anti-SATB1. Input (lanes 1 and 5) represents 5% of the nuclear extract used for IP. (B) In vitro competition assay was performed as described in Materials and Methods. TCF-4 contains all known functional domains of the TCF family proteins. Briefly, TCF-4 from nuclear extracts of Flag-TCF-4 transfected HEK 293 T cells was separately bound to GST-β-catenin (lanes 1–5) or GST-Arm (lanes 6–7) immobilized on glutathione-Sepharose. The bound complex was then incubated with various recombinant proteins as indicated, and the TCF-4 remaining bound to the column was monitored by Western blot analysis. “-” indicates that no protein was added and TCF-4 was eluted directly from the column. The upper panels represent WB analysis using anti-TCF-4, whereas the lower panel depicts Coomassie brilliant blue-stained SDS-polyacrylamide gel (15%) profile of various recombinant proteins used. (C) SATB1 and TCF-4 compete in vivo for association with β-catenin. Co-immunoprecipitation was performed to pulldown the SATB1:β-catenin complex from LiCl treated extracts from HEK 293T cells transfected with Flag-TCF4 (lane 2) or with empty vector (lane 1). IP and WB were performed using anti-SATB1 and anti-β-catenin, respectively. Expression of Flag-TCF4 and SATB1 was monitored by immunoblot analysis using respective antibodies as indicated in middle and lower panels. (D) SATB1 represses TCF targets in vivo. HEK 293T cells were transfected with indicated constructs for overexpression of full-length β-catenin (T41A), N-term β-catenin (1–567 aa), and SATB1 or empty vector in indicated combinations. β-catenin proteins encoded by both these constructs can translocate into nucleus and are therefore capable of transactivating Wnt/TCF/β-catenin targets; however, the N-term (1–576) protein does not interact with SATB1. RNA was isolated 48 h after transfection, and quantitative transcript profiling of TCF responsive genes integrin β1 and cyclin D1 was performed by real-time RT-PCR analysis as described in Materials and Methods.

Since we tested transcription of a number of genes that are targets of both SATB1 and TCF-1, we tested whether they interact with each other. Immunoprecipitation of thymocyte extract with anti-TCF-1 did not pulldown SATB1, suggesting a lack of their physical interaction (Figure 4A, lane 2). The TCF family of proteins consists of four members that are expressed as multiple isoforms through alternative splicing [34]. TCF-4 is the largest of all TCF family proteins containing all known functional domains, and therefore we employed it in the binding studies such that all possible domains including those that are not present in other TCF isoforms are also tested. TCF-4 is not known to play any role in T cell development [35] but plays an important role in epithelial cells such as HEK-293T [36]. We therefore used extract derived from control and LiCl treated HEK 293T cells for immunoprecipitation using anti-TCF-4. Immunoblot analysis using anti-SATB1 revealed an absence of interaction between SATB1 and TCF-4 even upon LiCl treatment (Figure 4A, lanes 5 and 6). After establishing that these two proteins do not interact with each other, we then explored whether they compete for association with β-catenin. We performed an in vitro competition experiment by loading TCF-4 on immobilized GST-β-catenin and GST-Arm proteins. The bound TCF-4 was then challenged separately with three functional domains of SATB1, namely PDZ, HD, and CD, and the TCF-4 remaining bound to the columns was then eluted and monitored by immunoblot analysis. Strikingly, although TCF-4 protein could be detected in eluates after incubation with HD or CD (Figure 4B, lanes 3 and 4), it was virtually undetectable after incubation with PDZ domain (lane 2), suggesting dissociation of TCF-4 from the β-catenin affinity matrix. In contrast, TCF-4 bound to the GST-Arm repeat affinity column was not displaced by PDZ domain (Figure 4B, lane 7), indicating that direct interaction between PDZ of SATB1 and the C-terminal region of β-catenin is required for its ability to compete with TCF-4. To monitor if TCF-4 and SATB1 compete in vivo, we performed co-immunoprecipitation analysis using extracts from HEK-293T cells transfected with control Flag vector or Flag-TCF4 and then treated with LiCl. Immunoprecipitation with anti-SATB1 yielded a marked reduction in β-catenin pulldown upon TCF-4 overexpression as compared to the vector control (Figure 4C). This observation provides strong evidence towards in vivo competition between SATB1 and TCF-4 for interaction with β-catenin. To test whether in vivo competition between these two transcription factors for recruitment of β-catenin affects gene expression, we monitored expression of two TCF responsive genes, integrin β1 and cyclin D1 [37],[38]. Overexpression of the T41A constitutively active β-catenin upregulated these genes (Figure 4D, bar 2). Expression of both these genes was negatively affected upon SATB1 overexpression presumably through titration of TCF-associated β-catenin (bar 6). When SATB1 and β-catenin were overexpressed simultaneously, the transcription of Integrin β1 and Cyclin D1 was reduced by about 2-fold (bar 3), indicating that overexpression of β-catenin partially derepresses the SATB1-mediated downregulation. However, overexpression of N-term (1–576) β-catenin does not yield a similar effect upon coexpression of SATB1 (Figure 4D, compare lanes 4 and 5), presumably since it lacks the SATB1-interacting C-terminal region of β-catenin. This result provides compelling evidence for requirement of SATB1: β-catenin interaction towards observed effects on gene regulation. Considered together, these data suggest that SATB1 mediates β-catenin signalling by competitively recruiting β-catenin and thereby also affects the transcription of TCF-regulated genes.

### SATB1 Is Deacetylated upon β-Catenin Signalling and Recruits β-Catenin to Its Target Genes

**Figure 5:**
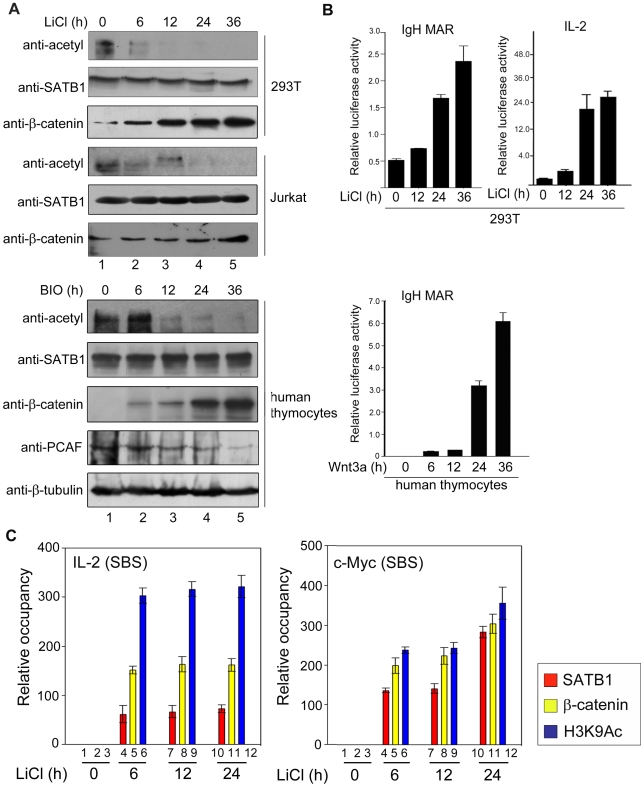
β-catenin signalling enhances SATB1 binding on its targets in vivo. (A) Deacetylation of SATB1 is a functional consequence of Wnt/β-catenin signalling. SATB1 was immunoprecipitated using nuclear extracts from LiCl treated HEK 293T and Jurkat cells and BIO treated human thymocytes at indicated time points, followed by WB with anti-pan-acetyl antibody. Lower panels indicate expression levels of SATB1 and β-catenin during the time course. While levels of SATB1 did not change much during this time course, β-catenin was progressively stabilized indicating that Wnt/β-catenin signalling is active in all three cell types. Immunoblot analysis of these extracts with anti-PCAF revealed downregulation of PCAF upon induction of Wnt signalling in human thymocytes. Immunoblot with anti-β-tubulin served as a loading control for thymocyte extracts. (B) MAR-linked reporter activity is upregulated upon induction of Wnt signalling. The activity of IgH-MAR and IL-2 reporter during the time course of LiCl treatment in HEK-293T cells or that of IgH-MAR reporter in Wnt3a treated human thymocytes was monitored as described in Materials and Methods. Each error bar represents standard deviation calculated from triplicates, and the *p* values are less than 0.001. (C) ChIP analysis was performed to monitor the occupancy of SATB1, β-catenin, and H3K9Ac at the SBSs within the *IL-2* and *c-Myc* loci as described in Materials and Methods. Change in occupancy was calculated from real-time PCR analysis of respective SBSs using anti-SATB1, anti-β-catenin, and anti-H3K9Ac immunoprecipitated chromatin from Jurkat cells treated with LiCl over 24 h. Occupancy at zero time was considered as base line control and the change in occupancy at other time points was calculated as relative occupancy with respect to the control.

The DNA binding activity of SATB1 is regulated by its post-translational modifications such as phosphorylation and acetylation. In particular, acetylation has a drastic negative influence on DNA binding activity of SATB1 [21]. To investigate the molecular mechanism of increased occupancy of SATB1 on its in vivo genomic targets, we monitored the acetylation level of SATB1 during the course of β-catenin accumulation. We performed immunoprecipitation of SATB1 in HEK 293T and Jurkat cells treated with LiCl over a time course of 36 h at an interval of every 6 h using SATB1 antibody followed by immunoblotting by pan-acetyl antibody. Interestingly, LiCl treatment led to a time-dependent decrease in acetylation of SATB1 in HEK-293T cells and Jurkat T cells (Figure 5A). We observed two bands of acetylated proteins in this study. However, molecular weight of SATB1 matched with the upper band only. The lower band could be due to immunoprecipitation of a SATB1 interacting protein that is also deacetylated upon Wnt signalling. While SATB1 levels did not change during the time course; as expected, β-catenin was stabilized over 36 h (Figure 5A). Similarly, upon BIO treatment we observed time-dependent deacetylation of SATB1 and accumulation of β-catenin over 36 h in primary human thymocytes (Figure 5A). Interestingly, PCAF acetyltransferase that is known to acetylate SATB1 [21] is downregulated in a time-dependent manner upon BIO treatment of thymocytes (Figure 5A), suggesting a probable mechanism for deacetylation of SATB1 during this time course. Consequently, IgH MAR and *IL-2* promoter-mediated reporter activity was upregulated in a time-dependent manner upon LiCl treatment (Figure 5B). To induce Wnt signalling in naïve T cells, we used the soluble Wnt3a ligand and performed IgH MAR-luciferase reporter assay. Upon treatment with Wnt3a, the reporter activity was stimulated more than 6-fold over 36 h, indicating stabilization of β-catenin and presumably its increased association with deacetylated SATB1. Deacetylated SATB1 has higher affinity for DNA [21] and recruits various cofactors such as β-catenin and p300 upon Wnt signalling, leading to the upregulation of MAR-linked transcription. Both IgH MAR and *IL-2* distal promoter reporter constructs harbour SBS [17] but do not contain the consensus TCF binding site; hence the observed upregulation is due to SATB1 only. Thus, these data argue that Wnt/β-catenin signalling induces deacetylation of SATB1, which then promotes its association with β-catenin and thereby alleviates SATB1 mediated repression. Next, to evaluate occupancy of SATB1-β-catenin to its genomic targets in a time-dependent manner, we monitored their occupancy at promoters of various genes containing SBS by ChIP. Upon LiCl treatment, SATB1 occupancy on *c-Myc* promoter is enhanced and β-catenin also follows a similar pattern (Figure 5C). Both *IL-2* and *c-Myc* are negatively regulated by SATB1 [15],[17] but are induced upon Wnt signalling [28], presumably by direct recruitment of the SATB1:β-catenin complex to their upstream regulatory regions containing the SBSs (Figure S7). Moreover, the time-dependent increase in the occupancy of the SATB1:β-catenin complex is accompanied with an increase in H3K9 acetylation at the promoters indicative of transcriptional activation (Figure 5C). Collectively, these findings further confirm that recruitment of β-catenin drastically alters the fate of SATB1-regulated genes.

### Wnt Signalling Is Active in Undifferentiated CD4^+^ T Cells and Differentiating T_H_ Cells

**Figure 6:**
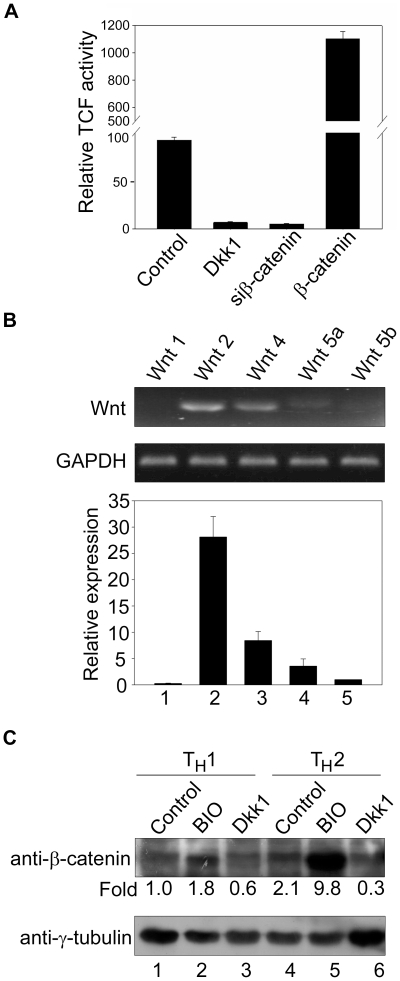
Wnt signalling is active in naïve CD4^+^ T cells and differentiating T_H_ cells. (A) Naïve CD4^+^ T cells were isolated from cord blood as described in Materials and Methods. Wnt signalling activity was measured by performing transactivation assay using TOPFlash and FOPFlash reporter constructs. TCF reporter activity was measured after 48 h in untreated control cells (bar 1), cells treated with Wnt inhibitor Dkk1 (bar 2), and upon cotransfections of si-β-catenin (bar 3) and β-catenin (T41A) (bar 4) as indicated. In all samples the reporter activity was measured without adding any Wnt agonist. The ratio of luciferase activities in TOPFlash transfected versus FOPFlash-transfected cells was determined and plotted as the relative TCF activity. Each error bar indicates standard deviation calculated from triplicates. (B) Expression of Wnts in CD4^+^ T cells. Naïve CD4^+^ T cells were isolated from cord blood as described in Materials and Methods. The mRNA levels of indicated Wnts were determined by RT-PCR analysis of total RNA extracted from these cells (top panel). Wnt expression was also monitored by quantitative RT-PCR analysis of RNAs from two biological replicates (lower graph). Expression level of Wnt 5b was considered as one unit for calculating relative fold expression of other Wnts after normalizing with GAPDH expression in the same sample. (C) Immunoblot analysis was performed to monitor stabilization of β-catenin in differentiating T_H_ cells. Naïve CD4^+^ cells were activated using plate-bound anti-CD3 and soluble anti-CD28 and differentiated by adding IL-4 or IL-12 as described in Materials and Methods. Nuclear extracts were prepared from control cells and cells polarized to T_H_1 and T_H_2 and were treated with Wnt agonist BIO or Wnt inhibitor Dkk1, followed by immunoblot analysis using anti-β-catenin (upper panel) and anti-γ-tubulin (lower panel). Each lane represents protein isolated from 5×10^6^ differentiated and treated cells. The numbers below the β-catenin panel represent fold change in β-catenin expression in the indicated lanes of the immunoblot upon normalization with that of γ-tubulin as loading control. Fold change values were calculated from densitometric quantitation of respective bands. Numbers below the γ-tubulin panel denote individual lanes of the immunoblot.

Expression of multiple members of the Wnt family has been documented in thymocytes [39]. However, expression of neither the Wnt molecules nor any of the downstream effectors has been documented in differentiating CD4^+^ T cells. To test whether Wnt signalling is active in CD4^+^ T cells, we isolated naïve CD4^+^ T cells from human umbilical cord blood and monitored transcription activity under the control of a concatemerized TCF binding site–driven reporter TOP-Flash in absence of any Wnt agonist. Control untreated CD4^+^ T cells showed low but consistent TCF reporter activity (Figure 6A, bar 1), suggesting that low levels of Wnt signals are produced by the T cells. Treatment with Dkk1 or transfection of siβ-catenin almost completely abolished the endogenous Wnt activity (Figure 6A, bars 2 and 3), suggesting that Wnt signalling is active in undifferentiated CD4^+^ T cells. Overexpression of the constitutively active T41A β-catenin yielded a dramatic increase in the TCF activity, indicating that CD4^+^ T cells are responsive to Wnt/β-catenin signals. The differential sensitivity of various subsets of thymocytes has been attributed to differential expression of the Wnt components [39]. To investigate whether CD4^+^ T cells isolated from cord blood and cultured ex vivo are responsive to Wnt signalling due to the Wnts produced by them, we examined the presence of several Wnts using RT-PCR and real-time quantitative RT-PCR. Most of the investigated Wnts appeared to be differentially expressed in these cells. Wnt 2 was the most prominent Wnt, followed by Wnt 4 and Wnt 5a (Figure 6B). Wnt 5b and Wnt 1 were expressed at extremely low levels and detected only by quantitative RT-PCR (Figure 6B). Wnt 5b is highly expressed in SP and DP thymocytes [39], and therefore it was used as a reference for calculating fold expression of other Wnts in CD4^+^ T cells. In second assay, the naïve CD4^+^ T cells were first activated with plate-bound anti-CD3 and soluble anti-CD28. T_H_1 and T_H_2 differentiation was then induced by adding cytokines IL-12 and IL-4, respectively, and cells were differentiated for 72 h. Expression of the T_H_1 marker cytokines IFN-γ and IL-12 as well as T_H_2 marker cytokine IL-4 in the cell supernatants was analyzed using the protein bead array to confirm their polarization (Figure S9). Stabilization of β-catenin upon differentiation was monitored by immunoblot analysis of nuclear extracts from the two subtypes of T_H_ cells. Endogenously stabilized β-catenin was detected in both T_H_1 and T_H_2 cells, although T_H_2 cells expressed 2-fold more β-catenin as compared to T_H_1 (Figure 6C, compare lane 4 with lane 1). To monitor the effect of Wnt signalling on the stabilization of β-catenin during differentiation, activated CD4^+^ T cells were differentiated for 72 h in the presence of soluble Dkk1 or Wnt agonist BIO. Treatment with Dkk1 resulted in reduction in endogenously stabilized β-catenin, which was more prominent in T_H_2 cells as compared to that of T_H_1 (compare lane 6 with lane 3). Treatment with BIO resulted in stabilization of β-catenin predominantly in T_H_2 cells (Figure 6C, compare lane 4 with lane 5), indicating that Wnt signalling was active in these cells. Thus, these results corroborate the finding that low levels of Wnt signals are produced by the T_H_ cells themselves and that the downstream processes such as stabilization of β-catenin also occur in the two subtypes of T_H_ cells, albeit to differential extent.

### Wnt Signalling and SATB1 Affect T_H_ Cell Lineage Commitment

**Figure 7:**
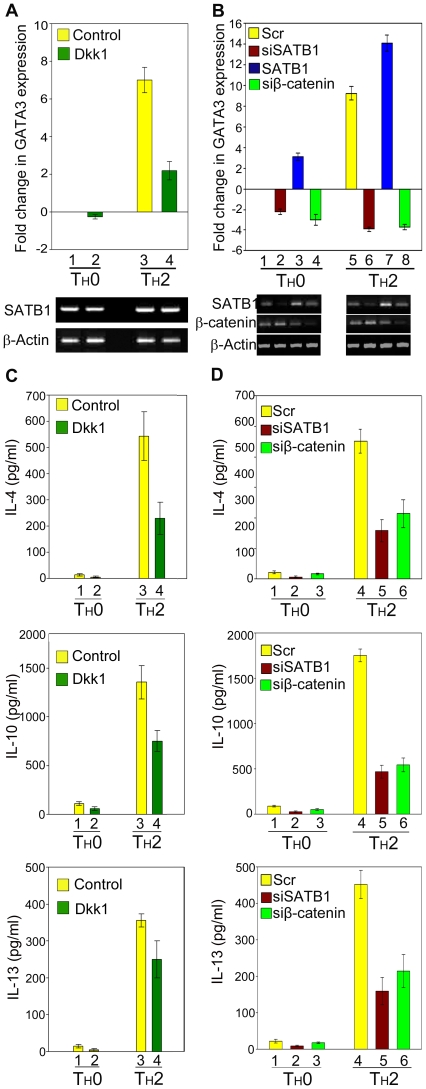
SATB1 and β-catenin regulate expression of GATA3 and T_H_2 cytokines in a Wnt-dependent manner in differentiating T_H_2 cells. (A) Active Wnt signalling is important for T helper cell differentiation. T_H_ cells were differentiated ex vivo in the presence or absence of Dkk1 as described in Materials and Methods [26]. Control cells were treated with the vehicle (1×PBS) only. Upon polarization for 72 h, total RNA was isolated and *GATA-3* transcripts were analyzed by quantitative RT-PCR as described in Materials and Methods. *GATA-3* expression was normalized with β-actin expression in these cells. The graph shows transcript levels of *GATA-3* in control and Dkk1 treated T_H_ cells. Fold changes were calculated with respect to the control T_H_0 subset in which the *GATA-3* expression level was set to baseline (bar 1). Each error bar represents standard deviation calculated from triplicates. Lower panels depict the corresponding transcript profile of SATB1 and β-actin. (B) SATB1 and β-catenin regulate *GATA-3* expression in T helper cells. Naïve CD4^+^ T cells were transfected with duplex siSATB1, siβ-catenin, or SATB1 overexpression plasmid DNA as described in Materials and Methods and differentiated ex vivo as described [26]. As control, duplex scrambled RNA (Scr) was transfected. Upon polarization for 72 h, total RNA was isolated and *GATA-3* transcripts were analyzed by quantitative RT-PCR as described in Materials and Methods. *GATA-3* expression was normalized with β-actin expression in these cells. The graph shows fold changes in *GATA-3* transcript in control (Scr) (bars 1 and 5), SATB1 silenced (bar 2 and 8), SATB1 overexpressed (bars 3 and 7), and β-catenin silenced (bars 4 and 8) T_H_0 and T_H_2 cells. Fold changes were calculated with respect to the scrambled RNA transfected T_H_0 subset in which the *GATA-3* expression level was set to baseline (bar 1). Each error bar represents standard deviation calculated from triplicates. Lower panels depict the corresponding transcript profile of SATB1, β-catenin, and β-actin in T_H_0 and T_H_2 cells upon knockdowns and overexpression, respectively. (C, D) Quantitation of T_H_2 cytokines Il-4, IL-10, and IL-13 in culture supernatants harvested from T_H_ cells grown for 72 h in the presence or absence of Dkk1 (C) or upon transfection of siSATB1 and siβ-catenin (D) was performed using a multiplex bead array reader as described in Materials and Methods. The expression of the indicated T_H_2 cytokines is presented in pg/ml, and each error bar represents standard deviation calculated from triplicates.

To test whether Wnt signalling is involved in T_H_ cell commitment, we monitored the effect of Wnt inhibitor Dkk1 on the transcription of the T_H_2 marker *GATA-3*. Naïve CD4^+^ T cells were isolated from human umbilical cord blood and activated with plate-bound anti-CD3 and soluble anti-CD28 (T_H_0 state) [26] and differentiated in the presence of specific cytokines as described above. Soluble Dkk1 was added to the medium in which T_H_2 cells were cultured. Cells were differentiated for 72 h and supplemented with Dkk1 after every 24 h. Expression of *GATA-3* was monitored as an indicator of the T_H_2 differentiation. Quantitative transcript profiling revealed that *GATA-3* expression was suppressed by over 3-fold upon Dkk1 treatment in T_H_2 subset, suggesting that Wnt signalling is necessary for the upregulation of *GATA-3* during differentiation of T_H_2 cells (Figure 7A). To directly assess the role of SATB1 in T_H_ cell differentiation, we altered their expression level by overexpression and siRNA mediated silencing and monitored the differentiation of CD4^+^ cells. Scrambled siRNA was used as a control. CD4^+^ cells transfected with siSATB1 or SATB1 expression construct were cultured for 24 h, activated with plate-bound anti-CD3 and soluble anti-CD28, polarized by adding cytokines, and grown further for 24 h. The expression of T_H_2 marker *GATA-3* was monitored by quantitative RT-PCR using total RNA extracted from these cells. Upon siRNA mediated silencing of SATB1 expression *GATA-3* was downregulated in T_H_0 and T_H_2 cells (Figure 7B). The expression of *GATA-3* in scrambled siRNA treated control T_H_0 cells was considered as baseline (Figure 7B, bar 1). Upon SATB1 knockdown, *GATA-3* expression was reduced by 2-fold in T_H_0 cells (bar 2). Notably, in T_H_2 cells *GATA-3* was downregulated more than 11-fold upon SATB1 knockdown (bar 6). The downregulation of *GATA-3* in T_H_2 was more striking as compared to the T_H_0 subset, indicating that SATB1 is required for *GATA-3* expression in T_H_2 cells. The influence of SATB1 on *GATA-3* expression was further confirmed by overexpression of SATB1 that led to a significant increase in the expression of *GATA-3* in both subsets as compared to the respective controls (compare bars 3 and 7 with 1 and 5, respectively), suggesting that SATB1 positively regulates *GATA-3* expression. Under these conditions, the expression of another T_H_2-specific transcription factor c-Maf was not altered significantly (Figure S10), suggesting that the regulatory effect of SATB1 is specific for GATA-3 at least during the early differentiation of T_H_ cells.

The differentiation of T_H_2 cells is characterized by secretion of signature cytokines that are encoded by the T_H_2 cytokine locus [40]. Therefore, to monitor the effect of Wnt/β-catenin signalling and SATB1 on expression of signature interleukins from differentiated T_H_ cells, culture supernatants of T_H_ cells that were subjected to *GATA-3* expression profiling upon knockdown of SATB1 and β-catenin and also culture supernatants from Dkk1 treated T_H_ cells were collected. The expression of secreted interleukins in the control, Dkk1 treated, and siRNA treated cell supernatants was monitored using multiplex protein bead array. Strikingly, signature T_H_2 cytokines such as IL-4, IL-10, and IL-13 were downregulated upon Dkk1 treatment (Figure 7C) and upon knockdown of SATB1 as well as β-catenin (Figure 7D), independently confirming the requirement of SATB1 and Wnt/β-catenin signalling during T_H_ cell differentiation. Knockdown of SATB1 also led to dramatic downregulation of the IL-4 transcript as well as intracellular IL-4 levels in T_H_2 cells (Figure S11). Together, these results argue that SATB1 and Wnt/β-catenin signalling positively regulate expression of signature interleukins in differentiating T_H_2 cells.

### SATB1 Regulates *GATA-3* Expression in T_H_2 Cells by Recruiting β-Catenin and p300 on *GATA-3* Promoter in a Wnt-Dependent Manner

**Figure 8:**
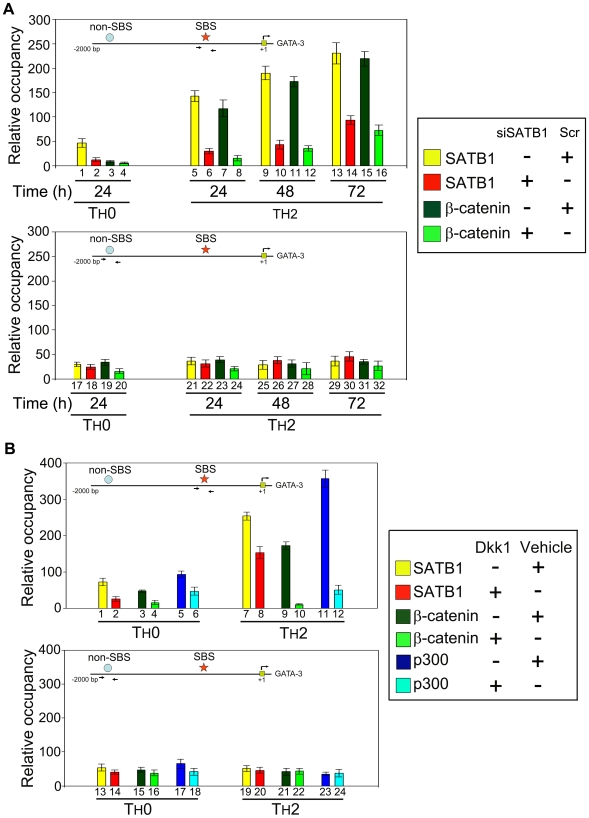
Recruitment of β-catenin and p300 by SATB1 at its binding site in *GATA-3* promoter is Wnt-dependent. (A) SATB1 recruits β-catenin at the SBS within *GATA-3* promoter. Naïve CD4^+^ T cells were differentiated to T_H_2 subtype by adding IL-4 for 24, 48, and 72 h in the presence or absence of siSATB1 or scrambled RNA (Scr) as described in Materials and Methods. Occupancy of the *GATA-3* promoter by SATB1 and β-catenin during T_H_2 differentiation was monitored by quantitative real-time PCR of the SBS (upper graph) and an upstream non-SBS region (lower graph) using chromatin immunoprecipitated from ex vivo differentiated T_H_ cells as described in Materials and Methods. Data represent relative occupancy of ChIP products as compared to the corresponding IgG controls, after normalizing for the input chromatin. Each error bar depicts standard deviation calculated from triplicates. (B) Naïve CD4^+^ T cells were differentiated to T_H_2 subtype by adding IL-4 for 72 h in the presence or absence of the Wnt inhibitor Dkk1 as described in Materials and Methods. Differential occupancy of the *GATA-3* promoter by SATB1, β-catenin, and p300 during T_H_2 differentiation was monitored by quantitative real-time PCR of the SBS (upper graph) and an upstream non-SBS regions (lower graph) using chromatin immunoprecipitated from ex vivo differentiated T_H_ cells. Vehicle (1×PBS) treated cells were used as controls. The occupancy of these three proteins on the two selected regions within *GATA-3* promoter was determined in T_H_0 and T_H_2 subsets. Data represent relative occupancy of ChIP products as compared to the corresponding IgG controls, after normalizing for the input chromatin. Error bars depict standard deviation calculated from triplicates. Insets depict schematic representation of the upstream 2 kb region of the *GATA-3* promoter showing relative positions of the SBS (star) and non-SBS (circle). Arrows depict positions of regions corresponding to which PCR primers were designed.

To understand whether SATB1 and β-catenin regulate *GATA-3* expression independently or they are functionally linked, we monitored the occupancy of SATB1 on the 1 kb upstream region from the transcription start site of *GATA-3* and found one potential SBS. Direct binding of SATB1 within this region encompassing around 600–900 bp upstream of the transcription start site was established in vitro by EMSA (Figure S12). Next, we monitored the occupancy of SATB1 in vivo using human CD4^+^ T_H_ cells in which expression of SATB1 was silenced using siSATB1. Cells transfected with scrambled RNA (Scr) served as the control. Both siSATB1 and Scr transfected cells were differentiated into T_H_2 cell lineage by adding IL-4 over the next 72 h. Cells that were only activated by plate bound anti-CD3 and anti-CD28 and not treated with IL-4 were referred to as T_H_0. We subjected these differentiated cells to ChIP analysis and monitored the occupancy of SATB1 and β-catenin at the SBS within the upstream region of *GATA-3* promoter by quantitative PCR analysis of the eluted chromatin. As a control for the ChIP analysis, we used another region at −1,500 to −1,800 bp of GATA-3 promoter that was not directly bound by SATB1 (non-SBS) (Figure S12). The occupancy of SATB1 gradually increased on promoter from 24 to 72 h of differentiation in control (Scr) T_H_2 cells (Figure 8A, bars 5, 9, and 13). SATB1 knockdown resulted in drastic reduction of its occupancy on *GATA-3* promoter (bars 6, 10, and 14). Strikingly, β-catenin occupancy also increased during the differentiation of control T_H_2 cells from 24 to 72 h and was significantly reduced upon SATB1 knockdown (compare bars 7, 11, and 15 with bars 8, 12, and 16, respectively). Such increase in the occupancy of SATB1 or β-catenin was not observed at the upstream non-SBS during the time course of differentiation (bars 21–32), suggesting that SATB1 recruits β-catenin at the SBS in *GATA-3* promoter.

To assess the contribution of β-catenin towards the positive influence of the SATB1:β-catenin complex on *GATA-3* promoter, we inhibited the Wnt signalling by treating the differentiating T_H_ cells with Dkk1. The T_H_ cells were differentiated as mentioned above and treated with Dkk1 for 72 h. The activated T_H_ cells (T_H_0) were used as controls. We differentiated cells for 72 h because the maximum occupancy of β-catenin and SATB1 on *GATA-3* was observed upon 72 h of differentiation. Chromatin isolated from differentiated T_H_0 (control) and T_H_2, Dkk1 treated, and untreated cells was subjected to immunoprecipitation using antibodies to SATB1, β-catenin, and p300. The occupancy of these proteins at the SBS within the *GATA-3* promoter was monitored during Wnt-on and -off conditions by quantitative PCR of immunoprecipitated DNAs. Upon Dkk1 treatment (Wnt-off), since the nuclear β-catenin level is reduced, its occupancy on *GATA-3* promoter was also reduced significantly (Figure 8B, compare bars 9 and 10). Occupancy of SATB1 was reduced at the SBS upon Dkk1 treatment (compare bars 7 and 8), suggesting that Wnt signalling is required for increased SATB1 occupancy on its genomic targets. Furthermore, p300 occupancy is also reduced at the *GATA-3* promoter upon Dkk1 treatment (compare bars 11 and 12), corroborating the observation that the SATB1:β-catenin complex recruits p300. This further explains the observed downregulation of *GATA-3* upon Dkk1 treatment (Figure 7A). Such changes in the occupancy of SATB1, β-catenin, and p300 were not observed at the non-SBS within *GATA-3* promoter (Figure 8A, lanes 19–24), indicating that Wnt signalling-dependent recruitment of SATB1 at the SBS in *GATA-3* promoter is required for upregulation of *GATA-3* during T_H_2 differentiation.

## Discussion

The non-random occupancy of SATB1 across the entire genome presumably results in the formation of a characteristic “cage-like” network in mouse thymocytes that demarcates heterochromatin from euchromatin [15],[20]. Furthermore, the “clustering” type occupancy exhibited by SATB1 at MARs and the upstream regulatory sequences of genes indicates a dichotomous role for SATB1 as a structural and regulatory chromatin component [19]. SATB1 presumably occupies the interchromatin territory or the nuclear space that contains genes that are transcriptionally poised [20]. What could be the consequence of sequestration of β-catenin by SATB1 in this nuclear compartment? The immunostaining of SATB1 and β-catenin in primary thymocytes indicates that they colocalize in the interchromatin space and form a distinguished substructure inside the nucleus. Since SATB1 is the only chromatin-associated protein known to harbour a PDZ-like signalling domain and the C-terminus of β-catenin interacts with SATB1 via this domain, it may also be speculated that the Wnt signalling cascade may cross-talk with the PDZ signalling cascade. β-catenin does not bind to DNA by itself; therefore SATB1 must recruit it to its genomic targets. Once recruited by SATB1 onto its genomic targets, β-catenin may then recruit the chromatin-modifying and -remodeling complexes to transcribe the Wnt target genes. This aspect is similar to the TCF/LEF family HMG box transcription factors, which need accessory factors to activate transcription at the site of their recruitment [34],[41]. The HMG box mediates sequence-specific binding to a core consensus sequence AGATCAAAGGG [42]. SATB1 also binds DNA in a sequence-specific manner to a 12-mer consensus sequence TATTAGTAATAT resembling the HD consensus [23]. Given that SATB1 and TCF have different recognition sites on the DNA, it is less likely that SATB1 can directly compete for TCF binding sites. Indeed, in vitro binding studies provide evidence that SATB1 does not bind directly to the TCF consensus element. However, since Wnt signalling also appears to affect the SATB1 responsive IgH-MAR-linked reporter in vivo, the possibility of SATB1's effect on TCF binding sites cannot be excluded. Many of the SATB1 regulated genes could also respond to Wnt signalling, and therefore this study has the potential to unravel hitherto unknown Wnt targets in T cells. In fact our gene expression profiling data unravelled ThPOK as a new Wnt target in thymocytes. ThPOK is repressed by SATB1 [21] (Limaye and Galande, manuscript in preparation) and is important for CD4 T cell development [43]. However, the precise mechanism of how only a specific subset of SATB1 targets is responsive to Wnt/β-catenin and not all targets requires further investigation.

The cellular expression levels of TCF and SATB1 could play a decisive role in determining the outcome of their interaction with β-catenin. We found that SATB1 competes with TCF for sequestering β-catenin. Since not all of SATB1 targets are Wnt targets and vice versa, the choice among these effector proteins as a partner of β-catenin could dictate the developmental fate of cells. There could be functional redundancy between SATB1 and TCF because TCF is expressed in some cell types but SATB1 is not and vice versa. After 48 h of differentiation, T_H_1 cells predominantly express TCF-1 and SATB1 is expressed at low levels, whereas T_H_2 cells express SATB1 only [26]. It would be interesting to study the mechanism that determines the occupancy of SATB1 and TCF-1 on promoters when both these proteins are expressed simultaneously in the same cell. One possibility that we have tested is that of their competition to occupy the genomic targets by interacting with β-catenin. TCF interacts with β-catenin via arm repeats, whereas SATB1 interacts with its C-terminus and therefore this competition is not driven by the site of interaction on β-catenin. Thus, the competition could be driven by the post-translational modifications and associated partners of these proteins. The DNA binding sites for TCF and SATB1 are not similar, and therefore the competition is not at the level of DNA binding but at the level of protein-protein interactions. Strikingly, upon SATB1 overexpression, TCF responsive genes that are not SATB1 targets are negatively affected in a β-catenin-dependent manner, indicating titration of β-catenin.

A number of studies have specifically addressed the relevance of Wnt signalling for lymphocyte development and typically involve genetic and/or biochemical analyses of TCF functions [2],[32],[35],[39],[44]–[48]. Wnt signalling is essential for T cell development in thymus, and it occurs in all thymocyte subsets but to different extents [2],[35],[39],[45]. The differential sensitivity to Wnt signalling is attributed to differential expression of Wnt(s) and its receptor and that of activating signalling molecules such as the long form of TCF-1 and β-catenin in different subsets of T cells [39]. The high redundancy of various Wnts and Fz in thymus masks their importance in knockout mouse models [2]. However, the involvement of β-catenin in T cell development could be elucidated by overexpression of the negative regulators of the Wnt/β-catenin pathway [10]. Studies of the molecular mechanisms of regulation of gene expression in T cells upon Wnt/β-catenin signalling have also been restricted to the TCF/LEF family proteins [34]. Our findings of physical association between SATB1 and β-catenin and its role in regulation of transcription of multiple genes provides an unprecedented clue towards the role of Wnt/β-catenin signalling in T cell development and differentiation. SATB1 is known to orchestrate spatio-temporal expression of multiple genes during T cell development [21],[25]. The effect of SATB1 deficiency on thymocyte development resembles that of β-catenin or TCF-deficient thymocytes [11],[25],[46]. Although Wnt signalling is known to occur in all thymocyte subsets, it occurs predominantly in the double negative (DN) subsets due to the high expression of activating Wnt transducers such as β-catenin and TCF-1 [39]. Interestingly, in *SATB1* deficient mice, DN cells were greatly reduced in number [25]. Wnt/β-catenin signalling also plays a role in late stages of thymocyte development such as differentiation into CD4^+^CD8^+^ DP cells [47], generation of mature CD8^+^ SP thymocytes [48], and differentiation of CD4^+^ SP cells to T_H_2 cells (this investigation). SATB1 presumably contributes to all of the above since in *SATB1* null mice the development of thymocytes is arrested at the DP stage, very few CD8 SP cells are observed [25], and *SATB1* is upregulated early during T_H_ cell differentiation [26]. However, SATB1 has not been directly shown to be important for T_H_ cell development, and the role of Wnt signalling in this process is also unprecedented. We show that CD4^+^ T cells are receptive to Wnt signals presumably because they produce different Wnts themselves. The differential sensitivity of T_H_ cell subtypes to Wnt signalling could be due to the fact that the downstream processes such as stabilization of β-catenin occur prominently in the T_H_2 subtype. Our study defines the role of SATB1:β-catenin collaboration in this important biological phenomenon. Further, SATB1 reprogrammes chromatin organization and gene expression profiles to promote breast tumor growth and metastasis [49]. Our study provides a plausible mechanism for such reprogramming via recruitment of β-catenin and chromatin modifying machinery. The implications of SATB1-mediated orderly deposition of β-catenin and its partners on chromatin towards global gene regulation upon Wnt signalling in various developmental systems and during tumorigenesis await further investigation.

In light of our findings, we propose that in absence of Wnt signalling when β-catenin is phosphorylated and degraded, SATB1 acts predominantly as a repressor on most of the genes and recruits different chromatin remodelling complexes to its genomic targets. However, upon β-catenin stabilization, it associates with β-catenin inside the euchromatic nuclear compartment, and its binding to target DNA is enhanced along with recruitment of β-catenin and H3K9 acetylation. Thus our study provides a mechanism explaining how SATB1's regulatory functions are modulated from repressor to activator in a signal-dependent manner. SATB1 acts as a “landing platform” for chromatin remodelling factors [16] and depending upon its post-translational modification status may selectively use its associated partners to modulate target gene transcription [21]. Wnt signalling activates the SATB1 target genes, which are otherwise repressed by SATB1. This switching mechanism is similar to that reported for the TCF:β-catenin complex, where interaction of β-catenin with TCF overcomes the repressor effect and multiple genes are activated [34]. Similarly, the β-catenin-HD containing protein Prop1 complex also works as a binary switch to simultaneously activate expression of the critical lineage-determining factor *Pit1* and represses the gene encoding the lineage-inhibiting transcription factor *Hesx1* acting via the TLE/Reptin/HDAC1 corepressor complexes [50]. This function resembles that of MyoD, CREB, and STAT-1 factors that simultaneously interact with several modifiers and selectively utilize their enzymatic activities for promoter stimulation [51],[52].

Deacetylation of SATB1 upon Wnt signalling is a key event modulating transcription. Acetylation has been shown to abrogate the DNA-binding activity of SATB1 [21] and therefore deacetylation is mandatory for enhancing its occupancy on genomic targets. However, acetylation of these two proteins has opposing effects on their association and ability to regulate transcription (Notani and Galande, manuscript in preparation). Thus, Wnt signalling leads to deacetylation of SATB1 that facilitates its interaction with β-catenin and thereby results in enhanced recruitment of SATB1 on its genomic targets. The Histone Acetyl Transferases (HATs) that acetylate these two proteins, namely PCAF and p300, respectively, may therefore regulate SATB1-mediated transcription upon Wnt signalling. TCF, the transcription factor known to recruit β-catenin on Wnt target genes, requires p300 as a cofactor in order to work as an activator [7], whereas SATB1 is dependent on PCAF for such functions [21]. Thus, post-translational modifications of TCF, SATB1, and β-catenin may fine tune the transcriptional outcome of the Wnt signalling cascade. Interestingly, PCAF itself is downregulated upon Wnt signalling in human thymocytes, especially at later time points. However, at very early time points (up to 9 h upon induction of Wnt signalling), PCAF is upregulated and so is the acetylation of SATB1, leading to its dissociation from the CtBP1 corepressor [53]. Therefore, acetylation seems to be the molecular switch governing the ability of SATB1 to function as an activator or repressor of multiple genes in a Wnt-dependent manner.

Role of SATB1 in regulation of T_H_2 specific cytokine locus has been suggested wherein SATB1 facilitates the expression of *IL-4*, *IL-5*, *IL-13*, and *c-Maf* via formation of a densely looped, transcriptionally active, higher-order chromatin structure upon T_H_2 cell activation [18]. T_H_ cell differentiation is mediated by signalling proteins, including STAT4 and STAT6, resulting in expression of transcription factors such as *GATA-3* and *c-Maf* (in T_H_2 cells) or *T-bet* (in T_H_1 cells) [12]. GATA-3 mediates T_H_2 responses through three different mechanisms: induction of T_H_2 cytokine production, selective growth of T_H_2 cells, and inhibition of T_H_1 promoting factors [13],[54]. Moreover, GATA-3 expression is necessary and sufficient for T_H_2 polarization [55]. GATA-3 is a marker of T_H_2 cells and SATB1 is also upregulated in T_H_2 cells [26]. Therefore, to monitor the role of SATB1 in T_H_ cell differentiation, we silenced SATB1 expression and monitored the T_H_ cell differentiation by quantifying *GATA-3* expression. Interestingly, upon siRNA mediated knockdown of SATB1, *GATA-3* expression is drastically decreased, indicating that T_H_2 polarization is SATB1-dependent. This is further substantiated by results of SATB1 overexpression in T_H_2 cells, which clearly establish SATB1 as a positive regulator of *GATA-3*. The Wnt/β-catenin signalling is active in CD4^+^ T_H_ cells as revealed by stabilization of β-catenin. The differential stabilization of β-catenin in T_H_1 and T_H_2 subtypes may explain the differential pattern of gene expression in these cells. We therefore used Dkk1 treatment to inhibit the active Wnt signalling in T_H_2 cells. Upon Dkk1 treatment, *GATA-3* expression was reduced drastically, suggesting that *GATA-3* is regulated in a Wnt/β-catenin signalling-dependent manner. Notably, c-Maf is not regulated by SATB1 or β-catenin under these conditions. Thus, at least during the early commitment and differentiation of human CD4^+^ T_H_ cells, SATB1 does not seem to regulate c-Maf. SATB1 regulates GATA-3 by directly binding to its promoter elements and may not regulate c-Maf in this manner. However, further studies may be required to address the role of SATB1 in regulation of c-Maf during different stages of T_H_ differentiation since positive regulation of c-Maf by SATB1 has been demonstrated in the differentiated mouse T_H_2 cell line (clone D10) [18]. Most strikingly, the expression of signature T_H_2 cytokines such as IL-4, IL-10, and IL-13 is also positively regulated by both SATB1 and Wnt signalling, as evident from their downregulation upon silencing of SATB1 or β-catenin and inhibition of Wnt signalling by Dkk1. The early time points in differentiation as employed in this study do not yield a very high amount of secreted cytokines in cell culture supernatants. Interestingly, at the RNA level as well as at the intracellular protein level, dramatic downregulation of IL-4 was observed. The secretion of IL-4 and other cytokines presumably surges after 72 h of differentiation. However, the silencing effect of siRNA would not last over such a long time period, and therefore effects of siSATB1 or siβ-catenin could not be monitored beyond 72 h in culture. Intriguingly, *IL-10* gene is located outside of the T_H_2 cytokine locus and yet is co-ordinately regulated by SATB1 in a Wnt-dependent manner. In *GATA-3*-deficient T_H_2 cells, production of signature T_H_2 cytokines such as IL-4 and IL-13 as well as that of IL-10 was also reduced, indicating that *GATA-3* is a major orchestrator of coordinated T_H_2 response [13]. Furthermore, ectopic expression of *GATA-3* induced T_H_2 cytokine expression in both differentiating and irreversibly committed T_H_1 cells [56]. Therefore, it is established that change in *GATA-3* expression directly affects T_H_2 differentiation. Thus, in the presence of the inhibitor of Wnt signalling (Dkk1) or upon knockdown of β-catenin, T_H_2 differentiation seems to be affected due to the downregulation of *GATA-3*. We therefore propose that SATB1 mediates T_H_2 differentiation by regulating *GATA-3* expression and thereby the expression of signature T_H_2 cytokines in a Wnt/β-catenin-dependent manner. Interestingly, a recent study reported that nephric duct-specific inactivation of *GATA-3* leads to massive ectopic ureter budding, suggesting that GATA-3 acts downstream of β-Catenin signalling to prevent ectopic metanephric kidney induction [57]. A recent study using TCF-1 and β-catenin deficient mice established that TCF-1 initiates T_H_2 differentiation of activated CD4^+^ T cells by promoting GATA-3 expression and suppressing IFN-γ expression [58]. Thus, there is increasing evidence that *GATA-3* is regulated by Wnt signalling.

Our study also provides a molecular mechanism for regulation of *GATA-3* expression by the SATB1:β-catenin complex in a Wnt signalling-dependent manner. ChIP analysis of an SBS within the *GATA-3* promoter in differentiating T_H_2 cells revealed a time-dependent increase in occupancy of SATB1 and β-catenin. Upon silencing of SATB1, its occupancy was expectedly decreased, but remarkably, even that of β-catenin was decreased by several-fold, suggesting that SATB1 is the key factor mediating the recruitment of β-catenin on *GATA-3* and presumably other targets. The recruitment of the SATB1:β-catenin complex on *GATA-3* promoter is sensitive to Dkk1 treatment. Moreover, since β-catenin recruits p300 on SATB1 genomic targets, the occupancy of p300 is also reduced concomitantly as that of β-catenin, presumably leading to the observed downregulation of *GATA-3* upon inhibition of Wnt signalling by Dkk1 treatment. Although control T_H_0 cells also show a similar Dkk1 sensitive pattern of occupancy of these three factors on the *GATA-3* promoter SBS, their fold occupancy is significantly more in the T_H_2 cells. The occupancy of the SATB1:β-catenin complex is very low at a non-SBS located upstream on the *GATA-3* promoter and is not sensitive to Dkk1. As with multiple other genes, the differential occupancy of various factors on different regions of the same promoter seems to be the mechanism governing regulation of transcription for *GATA-3*. The intriguing observation is that such differential recruitment is dependent on Wnt signalling. Thus, our results provide a molecular mechanism towards understanding the role of Wnt signalling in T_H_2 differentiation and define a unique role of SATB1 in this process.

## Materials and Methods

### Antibodies and Reagents

Antibodies to β-catenin and SATB1 were purchased from BD Biosciences. Antibodies to PCAF and pan-acetyl lysine were from Santa Cruz Biotechnology; antibodies to N-terminus of β-catenin, VP-16, and H3K9ac were obtained from Upstate Technologies. Anti-Flag was procured from Sigma-Aldrich Corp. siSATB1 and siβ-catenin duplex RNAs were from Santa Cruz Biotechnology, and BIO was obtained from Calbiochem. Recombinant Wnt3a, Dkk1, and cytokines were obtained from R&D Systems. Anti-SATB1 used for IP and WB was raised in rabbit and was purified using immunoaffinity chromatography using standard procedures.

### Plasmids

Expression constructs for β-catenin mutants T41A and GST 1–12 arm repeats were kindly provided by Dr. C. Neuveut, and pGEX6P3-β-catenin was gifted by Dr. M. Dunach. Deletion construct pGEX6P3-β-catenin-1–535 was generated by digesting pGEX6P3-β-catenin with SpeI and XhoI and relegating the larger fragment into pGEX6P3. pGEX6P3-β-catenin was digested with EcoRI, and the 1 Kb fragment was subcloned into pGEX6P2 to obtain pGEX6P2-β-catenin-425–781. RFP-fused versions of truncated β-catenin were produced by subcloning regions of β-catenin cDNA corresponding to aa 1–576 and 577–780 in pDsRed Express C1 vector (Takara Clontech). Various VP16-β-catenin truncations and Gal4DBD:SATB1, and Gal4DBD:PDZ fusions were cloned in pACT and pBIND plasmids (Promega), respectively. TOP and FOP reporter constructs were kind gifts by Dr. R. T. Moon. pTriEx-SATB1, various SATB1 domain constructs, and the reporter constructs pGL3-Basic-IgH MAR and pGL3-Basic-IL-2 have been described previously [17],[21].

### Immunostaining

Thymocytes were isolated from 3-wk-old Balb/C mice. Thymocytes were fixed using 2% paraformaldehyde and were permeabilized with 0.1% Triton X-100 followed by antibody staining of SATB1 and β-catenin (BD Biosciences). The secondary antibodies used were conjugated to Alexafluor dyes 488 and 594 (Invitrogen). DNA counterstaining was performed using DAPI. Cells were visualized under an upright fluorescence microscope (model AxioImager Z1, Carl Zeiss), and digital images were enhanced using the Apotome module (Carl Zeiss). The sectional views of the stained cells revealing the signals from the nuclear interior were generated using the Axiovision software (Carl Zeiss).

### In Vitro Binding Assay

GST:1–178, GST:1–535, GST:425–781, and GST:Arm repeats of β-catenin and GST:PDZ, GST:CD+HD, GST:CD, and GST:HD of SATB1 were expressed and purified from *Escherichia coli* (*E. coli*). Specific fusion proteins were cleaved on column by caspase-6 to obtain GST-free proteins as described [59]. Intact GST-fusion proteins were incubated with glutathione Sepharose 4B beads (GE Healthcare) for 2 h. ^35^S-labeled SATB1 was prepared by coupled in vitro transcription and translation as per manufacturer's instructions (Promega). Full-length SATB1 was expressed as 6XHis-SATB1 in *E. coli* and was bound to Ni-NTA beads (Qiagen). Bound proteins were washed with 1× PBS and incubated either with affinity purified recombinant proteins or with 100 µg nuclear extract from Jurkat or SW 480 cells or with in vitro transcribed and translated SATB1 for 2–3 h. The complexes were then washed with 1× PBS containing 0.1% triton X-100 and eluted using 2× SDS-loading buffer, resolved by SDS-polyacrylamide gel electrophoresis and analyzed by immunoblotting.

### Cell Culture, Transfections, and Reporter Assays

HEK cell line 293T, colon cancer cell line SW480, and T cell lymphoblastoid Jurkat cells were cultured in DMEM or in RPMI 1640 (Invitrogen), respectively, supplemented with 10% FCS (Invitrogen). Cells were transfected with indicated expression constructs using Lipofectamine 2000 (Invitrogen). After transfections cells were cultured for 24 h and then either LiCl or BIO were added to media at final concentrations of 20 mM or 1.0 µM, respectively, and cells were cultured further for 24 h unless mentioned otherwise. Transactivation assays were performed after 48 h of transfections as described [17]. Briefly, cells were harvested and washed with 1×PBS, followed by lysis with Luclite reagent (Perkin Elmer) as per manufacturer's instructions. Luciferase counts were measured using TopCount (Packard) and plotted as relative activity using Sigmaplot ver.10. For monitoring Wnt/TCF activity, the TOPFlash and FOPFlash reporter assay system was used. HEK 293T cells or naïve CD4^+^ T cells were cotransfected with the indicated expression vectors and the TOPFlash or FOPFlash reporter constructs. Equal masses of DNA were used in each transfection, and vector DNAs were used for normalization of DNA if required in each transfection reaction. The ratio of luciferase activities in TOPFlash-transfected versus FOPFlash-transfected cells was calculated and plotted as the relative TCF activity. All reporter assays were repeated at least thrice using independently transfected HEK 293T cells unless mentioned otherwise, and bar graphs represent values +/− s.d. The statistical significance of observed differences was calculated by *t* test.

### Coimmunoprecipitation

Five hundred µg of nuclear extract was diluted three times using 1× PBS containing 0.1% Triton X-100 and was precleared using 1.0 µg of either rabbit or mouse IgG and protein A/G beads (Pierce). Precleared lysate was subjected to immunoprecipitation by incubating with anti-SATB1 or anti-β-catenin for 2 h; protein A/G beads were then added and mixed further by incubating on an end-to-end rotator at 4°C for 4 h. Protein complexes were analyzed by immunoblotting with respective antibodies.

### ChIP

ChIP was performed essentially as described [17]. Briefly, cells were crosslinked by addition of formaldehyde to 1% final concentration in media and incubation at room temperature for 10 min, neutralized with 125 mM glycine, and then subjected to sonication using Bioruptor (Diagenode) to fragment the chromatin to obtain 200–500 bp fragments. Sonicated chromatin was precleared with a cocktail containing 50% protein A/G beads slurry (Pierce), Salmon sperm DNA, and BSA. Precleared chromatin was incubated with specific antibodies and respective Ig types were used as isotype controls. Protein A/G bead cocktail was then added to pull down the antibody-bound chromatin and was subjected to elution using sodium biocarbonate buffer containing SDS and DTT. Eluted chromatin was de-crosslinked and protein was removed by treating with proteinase K. Purified immunoprecipitated chromatin was subjected to PCR amplification using specific primers. Input chromatin was used as a control.

### Real-Time PCR

Quantitative PCRs were performed using iCycler (BioRad) using iQ SYBR Green mix (Bio Rad). ΔCt values were calculated using the formula: ΔCt = (Ct _Target_−Ct _Input_). Fold differences in gene expression were calculated as follows: Fold difference = 2 ^(Treatment−Loading Control)^/2^ (Control−Loading Control)^. Fold changes were calculated by dividing the level of expression of the experimental sample with the corresponding control sample.

### Ethics Statement

This study was conducted according to the principles expressed in the Declaration of Helsinki. Studies involving human samples were approved by the institutional ethics committee for medical research, and tissues were obtained according to the guidelines of the committee. All patients provided written informed consent for the collection of samples and subsequent analysis.

### Isolation of Immature Human Thymocytes

Thymic tissue was obtained from local hospitals from children undergoing cardiac surgery who did not have any medical history of immunological abnormalities. The tissue was minced into fine pieces, and thymocytes were collected by grinding on tissue sieve in RPMI 1640 media. The thymocyte suspension was passed through a 70 µm sieve to remove clumps and debris. The thymocytes obtained in this manner were devoid of thymic epithelial cells as confirmed by lack of staining by epithelial/stromal cell markers EPCAM and UEA-1. Thymocytes from 3–4 donors were pooled to reduce individual variation.

### Isolation of Peripheral CD4^+^ Cells

Peripheral human mononuclear cells were isolated from the human umbilical cord blood procured from local hospitals using Ficoll-Paque (GE Healthcare). CD4^+^ T cells were selectively isolated from PBMCs using human CD4^+^ T cell isolation (BD Biosciences) as per manufacturer's instructions.

### Ex Vivo Polarization of T_H_1 and T_H_2 Cells

CD4^+^ T cells were cultured in RPMI 1640 media supplemented with 10% FCS (Invitrogen). Cells were cultured in 24 well plates precoated with 0.5 µg/ml of anti-CD3 and in the presence of 0.5 µg/ml soluble anti-CD28 (eBiosciences). T_H_1 polarization was induced using 2.5 ng/ml IL-12 and T_H_2 polarization with 10 ng/ml IL-4 (R&D Systems). After 48 h of polarization, 17 ng/ml of IL-2 (R&D Systems) was added. Polarized cells were grown further for various time points from 24 to 72 h.

### Cytokine Quantitation

Supernatants from the control and treated T_H_ cells were collected by centrifugation. Levels of various cytokines in these supernatants were measured using the Bio-Plex T_H_1-T_H_2 kit and the Bio-Plex protein array reader (Bio-Rad) as per manufacturer's instructions. Levels of secreted cytokines were normalized against the respective media.

### Transfection of T Cells and RNA Isolation

Isolated T cells were differentiated ex vivo as described [26]. Thymocytes and CD4^+^ T cells were electroporated using the Nucleofector device (Amaxa) according to manufacturer's instructions. Cells were cultured in RPMI 1640 media supplemented with 10% FCS for 24 h. After 24 h cells were activated and polarized as described above. Wnt signalling was induced by addition of purified recombinant Wnt3a (R&D Systems) at 50 ng/ml or inhibited by addition of Dkk1 (R&D Systems) at 400 ng/ml. After 72 h of polarization in presence or absence of Dkk1, cells were harvested and divided into three parts. Chromatin was prepared from two portions of cells as described above. Total RNA was isolated from the remaining one third portion of cells, and cDNA was prepared using the Cells-to-Signal kit (Ambion) as per the manufacturer's instructions.

## Supporting Information

Figure S1 **Common targets of SATB1 and β-catenin.** Expression profiling of cells in which β-catenin was overexpressed, knocked out, or mutated available on NCBI public domain GEO datasets GSE1473 [60] and GSE1579 [61] were analyzed against expression profiling of SATB1 overexpression series GSE4317 [21] as described below. Analysis of multiple arrays generated a gene list that contained commonly regulated genes between SATB1 and β-catenin signalling studies. The genes with significant *p* value (<0.05) are tabulated. Description of microarray datasets used: The data from datasets GSE1473, GSE2519, and GSE1579 are in Affymetrix format, whereas the dataset GSE4317 is in UHN cDNA array format. Data deposited in GSE4317 were generated in our lab and contain gene expression profiling data from Jurkat (T-cell) and HeLa (non-T cell), where the cDNA from control untransfected cells was referenced and cells overexpressing wild-type SATB1, SATB1-phosphorylation deficient S185A mutant, and SATB1-acetylation deficient K136A mutant were the “treated” cells. GSE1473 represents expression profiling of 293T cells infected with RCAS vector carrying β-catenin S37A mutant. β-catenin S37A mutant is oncogenic and more stable than the corresponding wild-type protein. GSE1579 contains analysis of skin of deltaNβ-cateninER transgenics following the activation of β-catenin for up to 7 d. Onset and duration of β-catenin activation in deltaNβ-cateninER transgenics was controlled by 4-hydroxytamoxifen. Results provide insight into how β-catenin induces hair follicle growth. Method for analysis of microarray data: The data from these four datasets were downloaded from GEO (www.ncbi.nlm.nih.gov/GEO). We used the RAW scores and normalized these scores with controls to obtain fold-increase/decrease. For the datasets where replicate samples were available, we used sample means to calculate the *p*-value for the two-tailed Student's *t* test. For other datasets we used the probe-means to calculate the *p*-value. In study for dataset GSE 1473 above, the *p*-values are for the genes affected because of constitutive expression of the S37A in the transfected cells and are also SATB1 targets. In study for dataset GSE 1579 above, the *p*-value is for the genes that are differentially expressed on day 7 as compared to day 0 and are SATB1 targets based on GSE 4317.
(0.05 MB TIF)

Figure S2 **SATB1 and β-catenin interact in vivo.** (A) Coimmunoprecipitation reactions was performed by separately incubating anti-β-catenin and anti-SATB1 antibodies with aliquots of Jurkat nuclear extract as described in Materials and Methods followed by Western blot (WB) using anti-SATB1 and anti-β-catenin, respectively. IgH, immunoglobulin heavy chain. (B) Expression levels of SATB1 and β-catenin in HEK 293T, Jurkat, and SW480 cells were compared by immunoblot analysis using respective antibodies. The cells were treated with LiCl for 24 h prior to preparation of nuclear extracts. Immunoblot analysis revealed that SATB1 is endogenously expressed in HEK 293 and Jurkat cells (lanes 1, 2) and not in SW480 cells (lane 3). β-catenin is expressed in all three cell lines (lanes 4–6); however, its expression level is highest in SW480 cells.
(1.58 MB TIF)

Figure S3 **Qualitative analysis of proteins used for in vitro pulldowns.** Coomassie stained gels depicting the purity of various GST-β-catenin (lanes 3–5, 9, 10) and SATB1 (lanes 13, 14) truncations used in GST pulldown experiments. His-SATB1 corresponds to 6× histidine-tagged full-length SATB1, CD+HD is the C-terminal half of SATB1 containing the DNA-binding region, whereas PDZ indicates the N-terminal 1–204 amino acids of SATB1 harbouring its PDZ-like signalling domain.
(0.24 MB TIF)

Figure S4 **β-catenin activates SATB1-mediated transcription.** The functional consequence of overexpression of SATB1 and constitutively active mutant β-catenin (T41A) on MAR-linked reporter assay was monitored as described in Materials and Methods. The IgH-MAR-luciferase construct consists of seven copies of the 25 bp AT-rich core of the IgH-MAR [62] that is a high affinity binding site for SATB1 [23],[24] cloned in the pGL3Basic vector (Promega). The IgH-MAR itself promotes the transcription of luciferase [30]. SATB1 is known to bind to this element and repress transcription of linked reporter [17]. Each error bar indicates standard deviation calculated from triplicates.
(0.10 MB TIF)

Figure S5 **Human thymocytes were isolated from neonatal thymii as described in Materials and Methods and co-cultured with the OP9 or OP9-DL1 cells as described** [**63**] **.** In brief, thymocytes were cultured on monolayers of OP9 or OP9-DL1 cells for 48 h in RPMI 1640 medium (Invitrogen) supplemented with 10% FCS, along with Wnt3a (10 µg/ml) (Wnt agonist) or DKK1 (100 ng/ml) (Wnt inhibitor). The co-cultures were also supplemented with rIL-7 (5 ng/ml) and FlT3 ligand (5 ng/ml) (R&D Systems). (A) The cells were harvested after 48 h and the cell viability was monitored by MTT assay using standard protocol. Briefly, Thiazolyl Blue Tetrazolium Bromide (USB) was added to the thymocyte culture at a concentration of 1 mg/ml and incubated for 1 h. Cells were harvested by centrifugation, and cell pellet was resuspended in DMSO and measured at 570 nm. The absorbance readings were converted to percentages in viability, and the viability of control untreated cells was normalized to 100%. (B) Cell death was also assessed by the trypan blue dye exclusion method after 48 h co-culture. Thymocytes were harvested after respective time points and resuspended in PBS. To the cell suspension, few drops of 0.4% Tryan blue were added and incubated for 5 min at room temperature. Cells were then observed using hemocytometer, and cells that have not internalized the dye due to active dye exclusion were counted and converted to percentage viability. The viability of control untreated cells was normalized to 100%. As shown in (A) and (B), no significant difference was observed in the viability of thymocytes treated with Wnt3a or DKK1 and co-cultured with control OP9 or OP9-DL1 cells. (C, D) Effect of Wnt signalling on the transcription status of representative Wnt regulated genes BCL-X_L_ (C) and PP2A (D) in thymocytes. Thymocytes were co-cultured with control OP9 or OP9-DL1 cells as described above. Quantitative RT-PCR analysis was performed using RNA extracted from control human thymocytes and thymocytes treated for 48 h with Wnt3a or Dkk1 as described in Materials and Methods. The values for gene expression in treated cells were normalized with respect to the untreated control, which was set to 1. Colour key on the right indicates various treatments and co-cultures.
(0.39 MB TIF)

Figure S6 **Analysis of SBSs in regulatory regions of multiple genes.** EMSA analysis was performed using ^32^P-labeled upstream regulatory regions from *CHUK*, *PPM1A*, *c-Myc* genes, and the major breakpoint region of *BCL2*. Purified GST was used as a control for the GST:SATB1 fusion protein. The multiple regions tested from the 1 kb upstream region of *CHUK* and *PPM1A* are depicted schematically on top of the EMSA panels. Two regions (one in proximal *CHUK* promoter and one in distal *PPM1A* promoter) bound SATB1 at very high concentrations indicating very low affinity. Stars indicate SBSs whereas circles denote non-binding regions. Panel on bottom left depicts the dose-dependent binding of recombinant SATB1 with a ^32^P-labeled region encompassing −1,183 to −1,383 bp of human *c-Myc* promoter.
(0.72 MB TIF)

Figure S7 **SATB1 and β-catenin occupy the SBSs in** ***IL-2*** **and** ***c-Myc*** **promoters in vivo.** (A) ChIP-PCR analysis in Jurkat cells showing the binding of SATB1 and β-catenin on *IL-2* promoter in presence (+) and absence (−) of LiCl. ChIP analysis was performed as described in Materials and Methods. LiCl treatment resulted in increased occupancy of SATB1 at IL-2 promoter SBS, which then presumably recruits β-catenin at this locus. Antibodies used for ChIP are indicated on top of lanes. (B) ChIP-PCR analysis of Jurkat cells treated with Wnt agonist BIO was performed as described in Materials and Methods. SATB1 and β-catenin both occupy the SBS of human *c-Myc* promoter under these conditions (top panel, lanes 2 and 3). An upstream region (−10 kb) of *c-Myc* was used as a negative control for ChIP (lower panel).
(0.11 MB TIF)

Figure S8 **SATB1 does not bind to the TCF consensus in vitro.** In vitro binding analysis of TCF consensus binding site using recombinant SATB1 was performed by EMSA as described in Materials and Methods. The various panels depict EMSA using ^32^P-labeled wild-type (lanes 1–5) and mutant TCF (lanes 6–10) consensus sequences. ^32^P-labeled IgH-MAR was used as a positive control for SATB1 binding (lanes 11–15).
(0.18 MB TIF)

Figure S9 **Confirmation of polarization of CD4^+^ T cells to T_H_1 and T_H_2 subsets.** Quantitation of marker cytokines in culture supernatants harvested from T_H_ cells grown for 72 h was performed simultaneously using a multiplex bead array reader as described in Materials and Methods. As expected, TH1 cells produced IFN-γ and IL-12 whereas T_H_2 cells produced IL-4, confirming that the cells were committed to the respective lineages.
(5.36 MB TIF)

Figure S10 **Analysis of c-Maf expression in differentiating T_H_ cells.** Naïve CD4^+^ T cells were transfected with duplex siSATB1, siβ-catenin, or SATB1 overexpression plasmid DNA and differentiated ex vivo as described in Materials and Methods. As the control, duplex scrambled RNA (Scr) was transfected. Upon polarization for 72 h, total RNA was isolated and *GATA-3* transcripts were analyzed by quantitative RT-PCR as described in Materials and Methods. c-Maf expression was normalized with β-actin expression in these cells. The graph shows relative changes in c-Maf transcript in control (Scr) (bars 1 and 5), SATB1 silenced (bars 2 and 6), SATB1 overexpressed (bars 3 and 7), and β-catenin silenced (bars 4 and 8) T_H_0 (bars 1–4) and T_H_2 (bars 5–8) cells. Changes in expression levels were calculated with respect to the scrambled RNA transfected T_H_0 subset in which the c-Maf expression level was set to 1 (bar 1). Each error bar represents standard deviation calculated from triplicates. Naïve CD4^+^ T cells were transfected with duplex siSATB1, siβ-catenin, or SATB1 overexpression plasmid DNA as described in Materials and Methods and differentiated ex vivo as described [26]. As the control, duplex scrambled RNA (Scr) was transfected. Upon polarization for 72 h, total RNA was isolated and *GATA-3* transcripts were analyzed by quantitative RT-PCR as described in Materials and Methods. *GATA-3* expression was normalized with β-actin expression in these cells. The graph shows fold changes in *GATA-3* transcript in control (Scr) (bar 1), SATB1 silenced (bar 2), SATB1 overexpressed (bar 3), and β-catenin silenced (bar 4) T_H_2 cells. Fold changes were calculated with respect to the scrambled RNA transfected T_H_0 subset in which the *GATA-3* expression level was set to baseline (bar 1). Each error bar represents standard deviation calculated from triplicates.
(0.11 MB TIF)

Figure S11 **IL-4 is downregulated upon siRNA-mediated silencing of SATB1 in differentiating T_H_2 cells.** Human CD4^+^ T cells were transfected with Scrambled (Scr) and si SATB1 synthetic duplex RNAs and then polarized to T_H_2 as described in Materials and Methods. After 72 h the cells were harvested and used for monitoring the expression of IL-4. (A) IL-4 gene expression profiling. Total RNA was isolated and IL-4 transcripts were analyzed by RT-PCR as described in Materials and Methods. GAPDH expression served as the control for amount of RNA in the RT-PCRs. (B) The CD4^+^ T cells were polarized under T_H_2 conditions and harvested after 3 d. Four h prior to harvesting the cells, Brefeldin A was added to the culture media. The cells were fixed using 1% paraformaldehyde for 20 min at room temperature. The cells were then permeabilized using 0.5% Saponin and stained with anti-IL-4-PE conjugate (BD Biosciences) for 30 min. Stained cells were acquired on flow cytometer (BD FACS Calibur) and analyzed using CellQuest software (BD Biosciences).
(1.96 MB TIF)

Figure S12 **Analysis of upstream regulatory regions of** ***GATA-3*** **.** In vitro binding analysis of *GATA-3* promoter using recombinant SATB1 was performed by EMSA as described in Materials and Methods. The binding of SATB1 across the 2 kb (+1 to −2,000 bp) upstream regulatory region of *GATA-3* was monitored by using ^32^P-labeled fragments of this region. We focused on a high affinity SBS at a region spanning −900 to −600 bp and a non-SBS at an upstream region encompassing −1,800 to −1,500 bp of the human *GATA-3* promoter. The schematic on top of the EMSA panels depicts the relative positions of these regions within the promoter. A star indicates position of SBS, whereas a circle denotes non-binding site.
(0.25 MB TIF)

Table S1 **List of genes and gene IDs.** The genes studied here or mentioned in the text are tabulated along with their respective gene IDs.
(0.05 MB DOC)

Video S1 **SATB1 and β-catenin colocalize in the nuclei of mouse thymocytes.** Triple stained immunofluorescence image (Alexa 488, Alexa 546, and DAPI). Thymocyte nuclei were counterstained with DAPI to mark the DAPI intense and less intense areas in the nuclei. The movie depicts a 3-D stack of images from top to bottom in the same thymocytes as in Figure 1A. The optical sections of the images were captured at an interval of 0.2 µm using the “Apotome” module of AxioImager Z1 upright fluorescence microscope (Carl Zeiss). The images were further processed using inverse theorem in Deconvolution software (Carl Zeiss). The “cage-like” staining pattern of both proteins circumscribing heterochromatic blobs overlaps at least partially throughout the thymocyte nucleus.
(0.05 MB WMV)

Video S2 **β-catenin forms a characteristic “cage-like” substructure in the nuclei of mouse thymocytes.** Single stained immunofluorescence image (Alexa 488). The movie depicts a 3-D stack of images from top to bottom in the same thymocytes as in Figure 1A. Images were captured using the Apotome module (Carl Zeiss) as described above. β-catenin staining pattern clearly resembles the “cage-like” distribution characteristic of SATB1.
(0.04 MB WMV)

## Abbreviations

ACF: ATP utilizing chromatin assembly and remodelling factor
Arm: Armadillo
BIO: 6-bromoindirubin-3′-Oxime
CBP: CREB Binding Protein
CD: Cut Domain
ChIP: Chromatin Immunoprecipitation
CREB: cAMP responsive element binding protein
C_T_: threshold cycle
DAPI: 4′,6-Diamidino-2-PhenylIndole
DKK1: Dickkopf 1
DL1: Delta-like Ligand 1
DMSO: Dimethyl Sulfoxide
DN: double negative
DP: double positive
EMSA: electrophoretic mobility shift assay
FCS: Foetal Calf Serum
FlT3: FMS-like Tyrosine Kinase 3
Fz: frizzled
GSK-3 β: Glycogen Synthase 3 beta
GST: Glutathione S Transferase
H3K9: Histone 3 Lysine 9
HAT: Histone Acetyl Transferase
HD: Homeo Domain
HDAC1: Histone Deacetylase 1
HEK: Human Embryonic Kidney
HMG: High Mobility Group
IFNγ: Interferon gamma
IL-10: Interleukin 10
IL-12: Interleukin 12
IL-13: Interleukin 13
IL-4: Interleukin 4
IL-7: Interleukin 7
IP: Immunoprecipitation
LEF: Lymphoid Enhancer Binding Factor 1
LiCl: Lithium Chloride
MAR: Matrix Attachment Regions
MTT: (3-(4,5-Dimethylthiazol-2-yl)-2,5-diphenyltetrazolium bromide)
PBS: Phosphate Buffered Saline
PCAF: p300/CBP-associated factor
PDZ: Post synaptic density protein (PSD95), Drosophila disc large tumor suppressor (DlgA), and Zonula occludens-1 protein (zo-1)
RFP: red fluorescent protein
RPMI: Roswell Park Memorial Institute
RT-PCR: Reverse Transcription-Polymerase Chain Reaction
SATB1: Special AT rich Binding protein 1
SBS: SATB1 Binding Sequences
SDS: sodium dodecyl sulfate
SP: single positive
STAT4: Signal Transducer and activator of Transcription 4
STAT6: Signal Transducer and activator of Transcription 6
T_H_: T Helper
TBP: TATA Binding Protein
TCF: T Cell Factor
TCR: T cell Receptor
WB: Western Blotting
Wnt: Wingless type
