# Expression of Concern: Global Regulator SATB1 Recruits β-Catenin and Regulates T_H_2 Differentiation in Wnt-Dependent Manner

**DOI:** 10.1371/journal.pbio.3001908

**Published:** 2022-11-23

**Authors:** The PLOS Biology Editors

After this article [1] was published, concerns were raised about similarities and discontinuities between some of the lanes in the western blot images in Figs 1, 5, S2, and S3, and between some of the lanes in the EMSA autoradiograms in Figure S6.

The corresponding author provided the original underlying images and quantitative data to support several figures in the article (S1 File), but stated that the original data are no longer available for the western blots in Fig 1B and 1D left panel, Figure 4B bottom panel, Figure 4C bottom panels, Figure 7B β-actin panels, Figure S3, Figure S7, Figure S8 and Figure S12 left panel. In some cases, the authors provided replication data to support the figures in question (S2 File).

Overall, the data provided resolved some but not all of the image integrity concerns.

Specifically:

- In the left western blot panel in Fig 1D there appear to be vertical discontinuities, and the pixel patterns appear similar between different areas within lane 5. The original underlying data for this panel were not provided and so these issues remain unresolved. The authors stated that lanes were removed during figure preparation, and replication data were provided (S2 File) that support the results reported in the article.
- In the right western blot panel in Fig 1D lanes 7 and 10 appear similar. The authors confirmed that the background from lane 10 seems to have been duplicated in lane 7. The underlying image provided for this experiment is in S1 File. The backgrounds of lanes 7 and 10 appear different in the underlying blot image (compared to each other and compared to the published figure for lane 7), but underlying data for both lanes indicated negative results.
- When levels are adjusted to visualise background, there appear to be discontinuities suggestive of image splicing in Figures S2A, S2B, S3, and S6. In Figure S3 the M lanes in the left and centre blots appear similar.
  - The images provided in S1 File to support the left western blot panel of Figure S2A do not appear to match the published figure panel and did not clarify the concern about discontinuities in the published figure. The published figure has a stronger and clearer interaction signal than the underlying images; however, the underlying images- while different from the published figure- show a band in the position that would indicate an interaction, and support the conclusion.
  - For Figures S2B and S6, the original data (S1 File) clarified the concerns. The raw data indicated that there was undeclared splicing in Figure S6 after lane 5 of the CHUK panel. For other panels in these figures the discontinuities observed in the figures were also present in the raw images.
  - For Figure S3, the authors stated that the original data are no longer available and so we were unable to resolve the concerns about the published figure. However, the authors provided replication data (S2 File) that support the results reported in the article.
- During our assessment of this case, faint bands were noted in the original image (S1 File) for the left western blot panel in Fig 1E that are not evident in the published figure. The corresponding author indicated that they used a lower exposure in the published image, the underlying image for which is no longer available. The data in S1 File are consistent with the conclusion that there is more signal with the PDZ domain than the CD+HD domains, although the difference between lanes in S1 File is not as striking as in the published figure.

The authors stand by the conclusions of the study and consider that the additional data provided support all original claims. The corresponding author has provided a revised schematic for Fig 1D below which shows where the two proteins interact and with the relevant amino acids identified.

Overall, *PLOS Biology* concluded that the article’s main conclusions appear to be supported. However, in light of the unresolved issues and the extent of concerns about the integrity of image data reported in the article, the *PLOS Biology* Editors issue this Expression of Concern.

**Fig 1:**
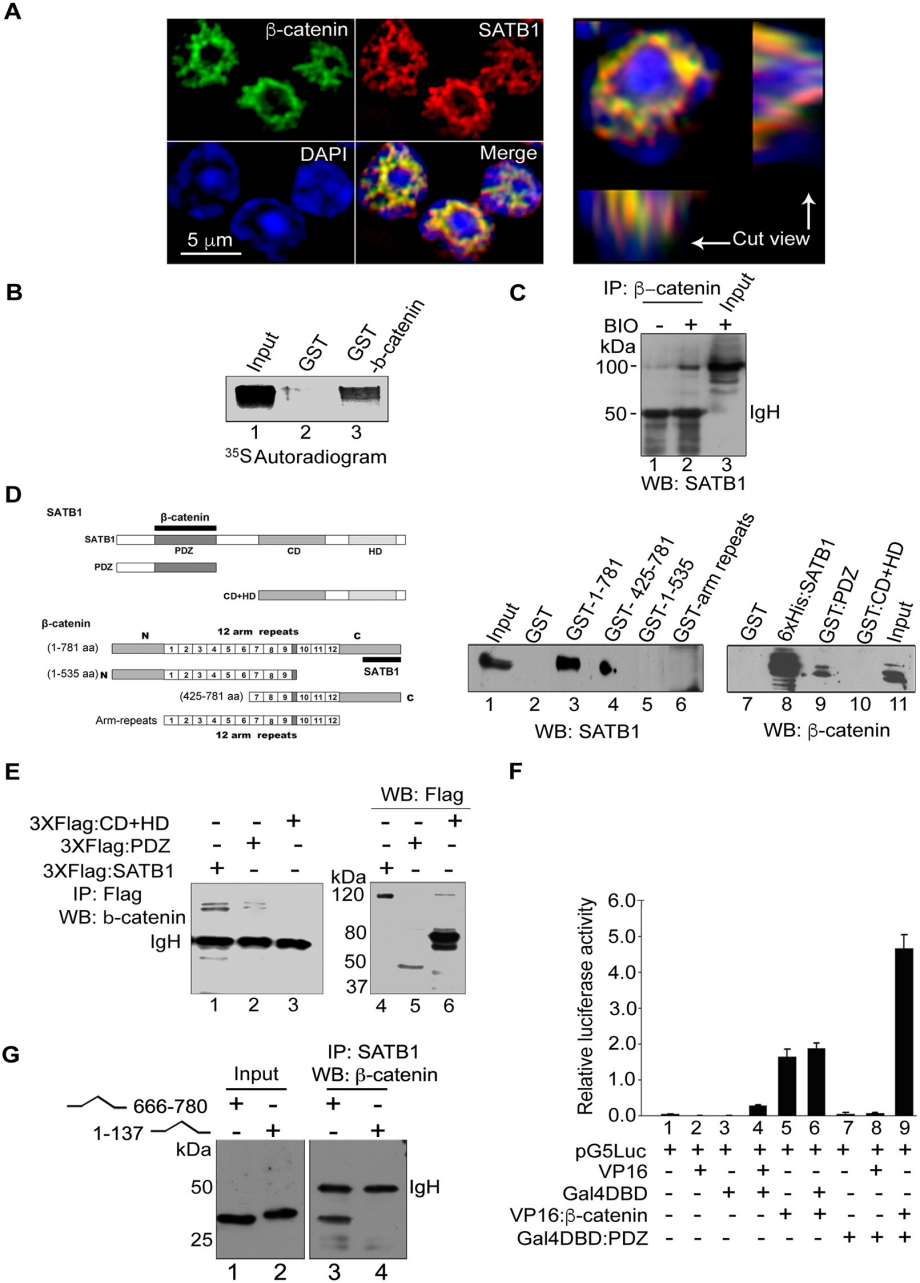
Delineation of physical interaction between SATB1 and β-catenin. (A) SATB1 and β-catenin colocalize in the thymocyte nuclei. Indirect immunofluorescence staining of thymocytes using antibodies to SATB1 (red) and β-catenin (green) was performed as described in Materials and Methods. DNA counterstaining was performed using DAPI (blue). The cut view panel depicts two perpendicular transverse sections of a triple-stained thymocyte as indicated by white lines, intersecting at the point of the brightest fluorescence signal. (B) Direct interaction between SATB1 and β-catenin was monitored by in vitro pulldown assays performed as described in Materials and Methods. ^35^S-labeled SATB1 was specifically pulled down after incubation with immobilized GST-β-catenin (lane 3) and not with control immobilized GST (lane 2). (C) In vivo interaction of SATB1 and bcatenin was assessed by performing coimmunoprecipitation analysis as described in Materials and Methods. Nuclear extracts derived from BIO treated (+) and control (-) human thymocytes were immunoprecipitated using anti-β-catenin followed by WB with anti-SATB1. (D) The interacting regions of SATB1 and β-catenin were mapped by in vitro pulldown assay. GST pulldowns of SATB1 and β-catenin were performed by passing Jurkat nuclear extract on immobilized domains of GST-β-catenin (lanes 1–6) and SW480 nuclear extract on immobilized domains of SATB1 (lanes 7–11) including both full-length proteins followed by WB with anti-SATB1 and anti- β-catenin. SATB1 and β-catenin truncations used are depicted schematically on the left. Solid black bars depict the respective interacting regions. (E) Coimmunoprecipitation analysis of extracts derived from HEK 293T cells overexpressing 3XFlag-SATB1 and its functional domains using anti-Flag antibody followed by WB with anti-β-catenin (lanes 1–3). Expression levels of the 3XFlag-fused domains of SATB1 were monitored by WB using anti-Flag (lanes 4–6). (F) Mammalian two hybrid assay was performed to score for protein-protein interactions in HEK 293T cells essentially as described [23]. The C-terminus of β-catenin and the PDZ-like domain of SATB1 were expressed as fusions with VP-16 and GAL4-DBD using the pACT and pBIND vectors of the CheckMate mammalian two-hybrid system (Promega). pBIND and pACT fusion constructs were transfected along with a reporter vector pG5-Luc containing 4xGAL4 responsive element and luciferase activity was compared with the control. Error bars represent standard deviation calculated from triplicates. (G) The C-terminus and not the N-terminus of β-catenin is involved in its interaction with SATB1. VP-16 fused C-terminal (aa 666–780, lane 1) and N-terminal (aa 1–137, lane 2) regions of β-catenin were overexpressed in HEK 293T cells. Co-immunoprecipitation was performed as described in Materials and Methods using anti-SATB1 followed by WB using anti-β-catenin.

## Supporting information

S1 File Original underlying images and quantitative data to support some figures. (ZIP)

S2 File Data from replicate experiments conducted at the time of the original experiments. (ZIP)
