# Supplementary figures and images for: Global Regulator SATB1 Recruits β-Catenin and Regulates TH2 Differentiation in Wnt-Dependent Manner
Source: PLoS Biol. 2010 Jan 26;8(1):e1000296. doi: 10.1371/journal.pbio.1000296 (PMC2811152; doi:10.1371/journal.pbio.1000296)

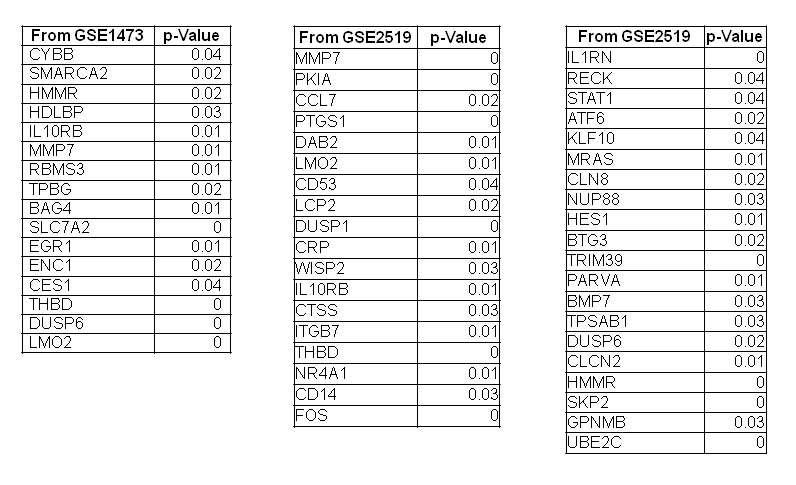

Supplement: Figure S1 — Common targets of SATB1 and β-catenin. Expression profiling of cells in which β-catenin was overexpressed, knocked out, or mutated available on NCBI public domain GEO datasets GSE1473 [60] and GSE1579 [61] were analyzed against expression profiling of SATB1 overexpression series GSE4317 [21] as described below. Analysis of multiple arrays generated a gene list that contained commonly regulated genes between SATB1 and β-catenin signalling studies. The genes with significant p value (<0.05) are tabulated. Description of microarray datasets used: The data from datasets GSE1473, GSE2519, and GSE1579 are in Affymetrix format, whereas the dataset GSE4317 is in UHN cDNA array format. Data deposited in GSE4317 were generated in our lab and contain gene expression profiling data from Jurkat (T-cell) and HeLa (non-T cell), where the cDNA from control untransfected cells was referenced and cells overexpressing wild-type SATB1, SATB1-phosphorylation deficient S185A mutant, and SATB1-acetylation deficient K136A mutant were the “treated” cells. GSE1473 represents expression profiling of 293T cells infected with RCAS vector carrying β-catenin S37A mutant. β-catenin S37A mutant is oncogenic and more stable than the corresponding wild-type protein. GSE1579 contains analysis of skin of deltaNβ-cateninER transgenics following the activation of β-catenin for up to 7 d. Onset and duration of β-catenin activation in deltaNβ-cateninER transgenics was controlled by 4-hydroxytamoxifen. Results provide insight into how β-catenin induces hair follicle growth. Method for analysis of microarray data: The data from these four datasets were downloaded from GEO (www.ncbi.nlm.nih.gov/GEO). We used the RAW scores and normalized these scores with controls to obtain fold-increase/decrease. For the datasets where replicate samples were available, we used sample means to calculate the p-value for the two-tailed Student's t test. For other datasets we used the probe-means to calculate the p-value. In stu [file pbio.1000296.s001.tif]

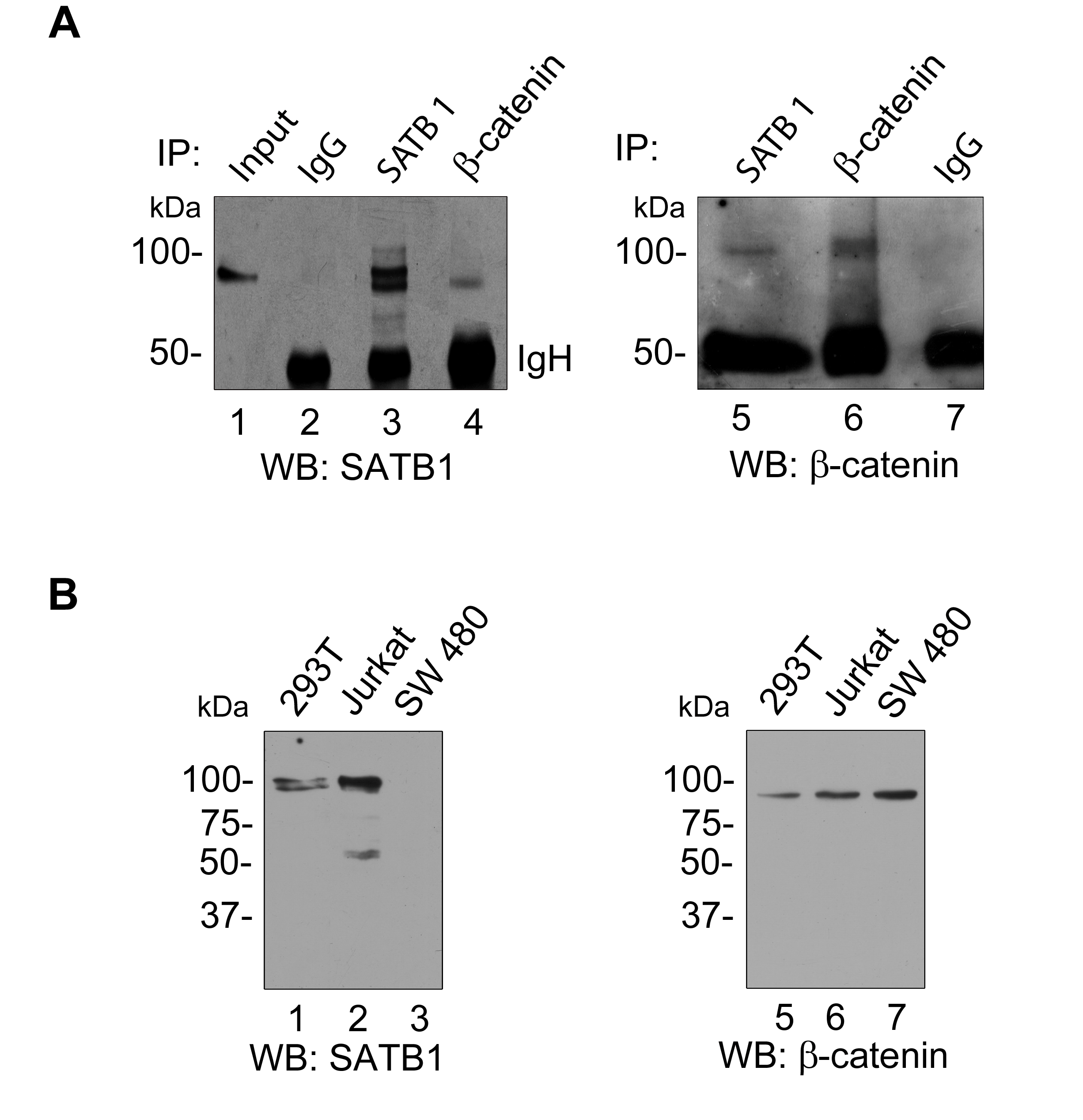

Supplement: Figure S2 — SATB1 and β-catenin interact in vivo. (A) Coimmunoprecipitation reactions was performed by separately incubating anti-β-catenin and anti-SATB1 antibodies with aliquots of Jurkat nuclear extract as described in Materials and Methods followed by Western blot (WB) using anti-SATB1 and anti-β-catenin, respectively. IgH, immunoglobulin heavy chain. (B) Expression levels of SATB1 and β-catenin in HEK 293T, Jurkat, and SW480 cells were compared by immunoblot analysis using respective antibodies. The cells were treated with LiCl for 24 h prior to preparation of nuclear extracts. Immunoblot analysis revealed that SATB1 is endogenously expressed in HEK 293 and Jurkat cells (lanes 1, 2) and not in SW480 cells (lane 3). β-catenin is expressed in all three cell lines (lanes 4–6); however, its expression level is highest in SW480 cells. (1.58 MB TIF) [file pbio.1000296.s002.tif]

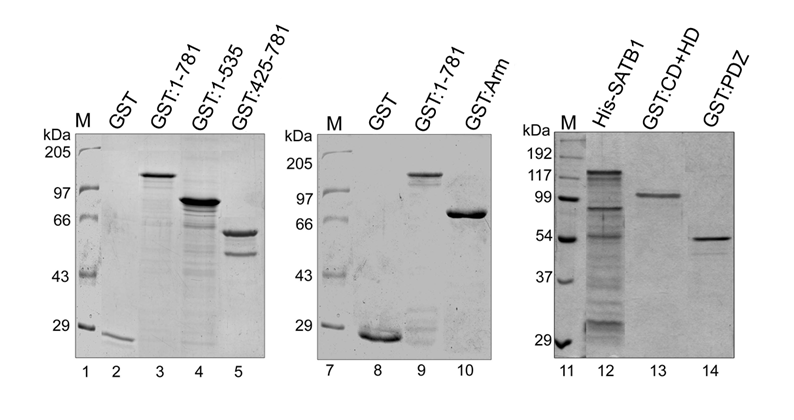

Supplement: Figure S3 — Qualitative analysis of proteins used for in vitro pulldowns. Coomassie stained gels depicting the purity of various GST-β-catenin (lanes 3–5, 9, 10) and SATB1 (lanes 13, 14) truncations used in GST pulldown experiments. His-SATB1 corresponds to 6× histidine-tagged full-length SATB1, CD+HD is the C-terminal half of SATB1 containing the DNA-binding region, whereas PDZ indicates the N-terminal 1–204 amino acids of SATB1 harbouring its PDZ-like signalling domain. (0.24 MB TIF) [file pbio.1000296.s003.tif]

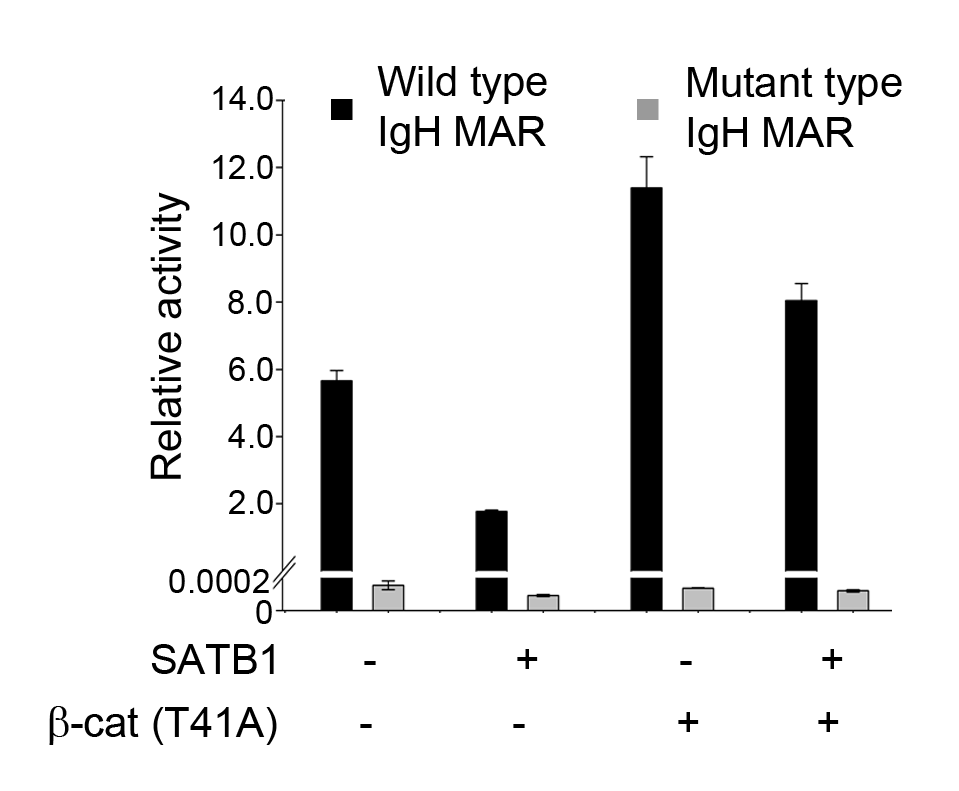

Supplement: Figure S4 — β-catenin activates SATB1-mediated transcription. The functional consequence of overexpression of SATB1 and constitutively active mutant β-catenin (T41A) on MAR-linked reporter assay was monitored as described in Materials and Methods. The IgH-MAR-luciferase construct consists of seven copies of the 25 bp AT-rich core of the IgH-MAR [62] that is a high affinity binding site for SATB1 [23],[24] cloned in the pGL3Basic vector (Promega). The IgH-MAR itself promotes the transcription of luciferase [30]. SATB1 is known to bind to this element and repress transcription of linked reporter [17]. Each error bar indicates standard deviation calculated from triplicates. (0.10 MB TIF) [file pbio.1000296.s004.tif]

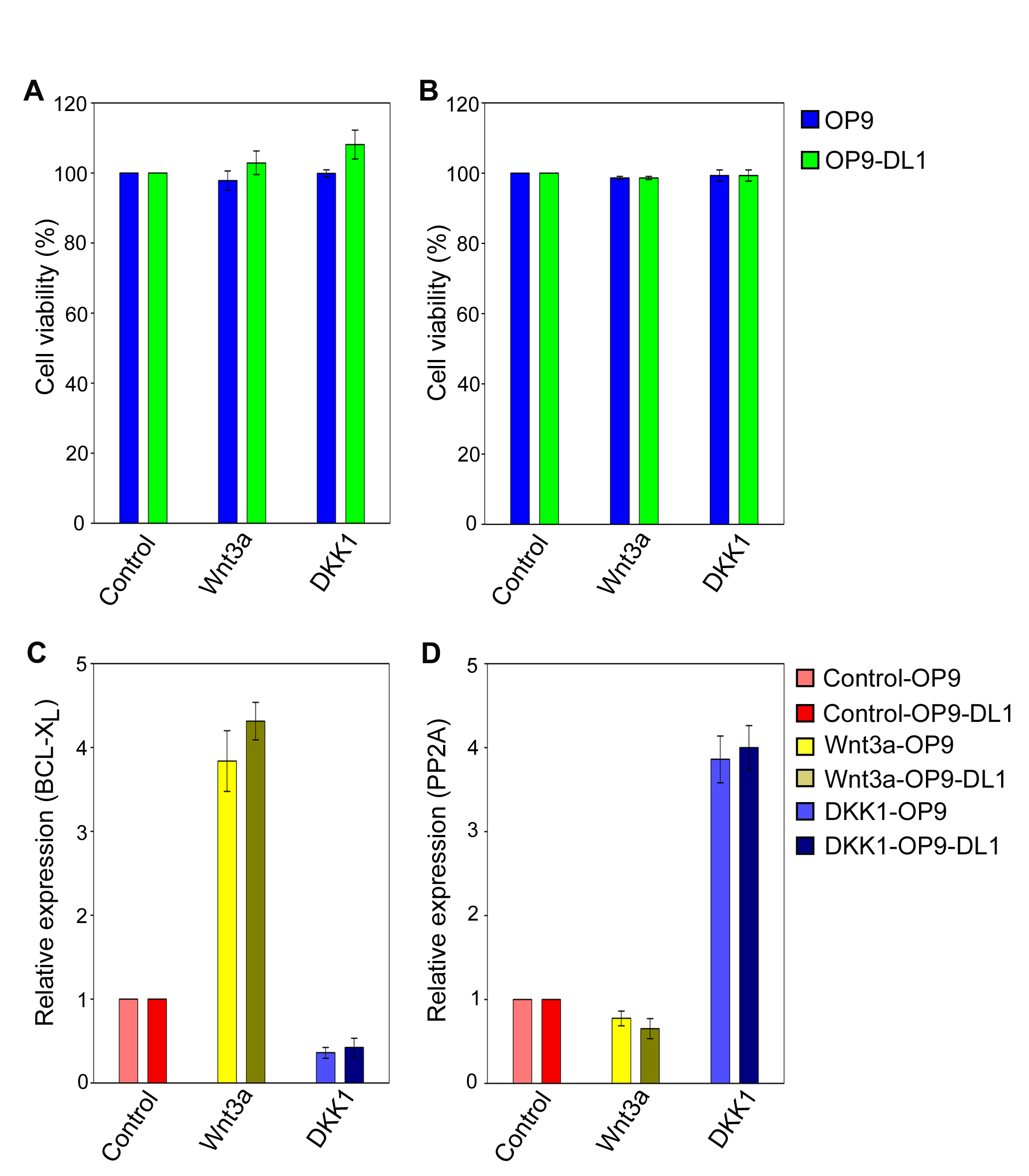

Supplement: Figure S5 — Human thymocytes were isolated from neonatal thymii as described in Materials and Methods and co-cultured with the OP9 or OP9-DL1 cells as described [63] . In brief, thymocytes were cultured on monolayers of OP9 or OP9-DL1 cells for 48 h in RPMI 1640 medium (Invitrogen) supplemented with 10% FCS, along with Wnt3a (10 µg/ml) (Wnt agonist) or DKK1 (100 ng/ml) (Wnt inhibitor). The co-cultures were also supplemented with rIL-7 (5 ng/ml) and FlT3 ligand (5 ng/ml) (R&D Systems). (A) The cells were harvested after 48 h and the cell viability was monitored by MTT assay using standard protocol. Briefly, Thiazolyl Blue Tetrazolium Bromide (USB) was added to the thymocyte culture at a concentration of 1 mg/ml and incubated for 1 h. Cells were harvested by centrifugation, and cell pellet was resuspended in DMSO and measured at 570 nm. The absorbance readings were converted to percentages in viability, and the viability of control untreated cells was normalized to 100%. (B) Cell death was also assessed by the trypan blue dye exclusion method after 48 h co-culture. Thymocytes were harvested after respective time points and resuspended in PBS. To the cell suspension, few drops of 0.4% Tryan blue were added and incubated for 5 min at room temperature. Cells were then observed using hemocytometer, and cells that have not internalized the dye due to active dye exclusion were counted and converted to percentage viability. The viability of control untreated cells was normalized to 100%. As shown in (A) and (B), no significant difference was observed in the viability of thymocytes treated with Wnt3a or DKK1 and co-cultured with control OP9 or OP9-DL1 cells. (C, D) Effect of Wnt signalling on the transcription status of representative Wnt regulated genes BCL-XL (C) and PP2A (D) in thymocytes. Thymocytes were co-cultured with control OP9 or OP9-DL1 cells as described above. Quantitative RT-PCR analysis was performed using RNA extracted from control human thymocytes and thymocytes treated [file pbio.1000296.s005.tif]

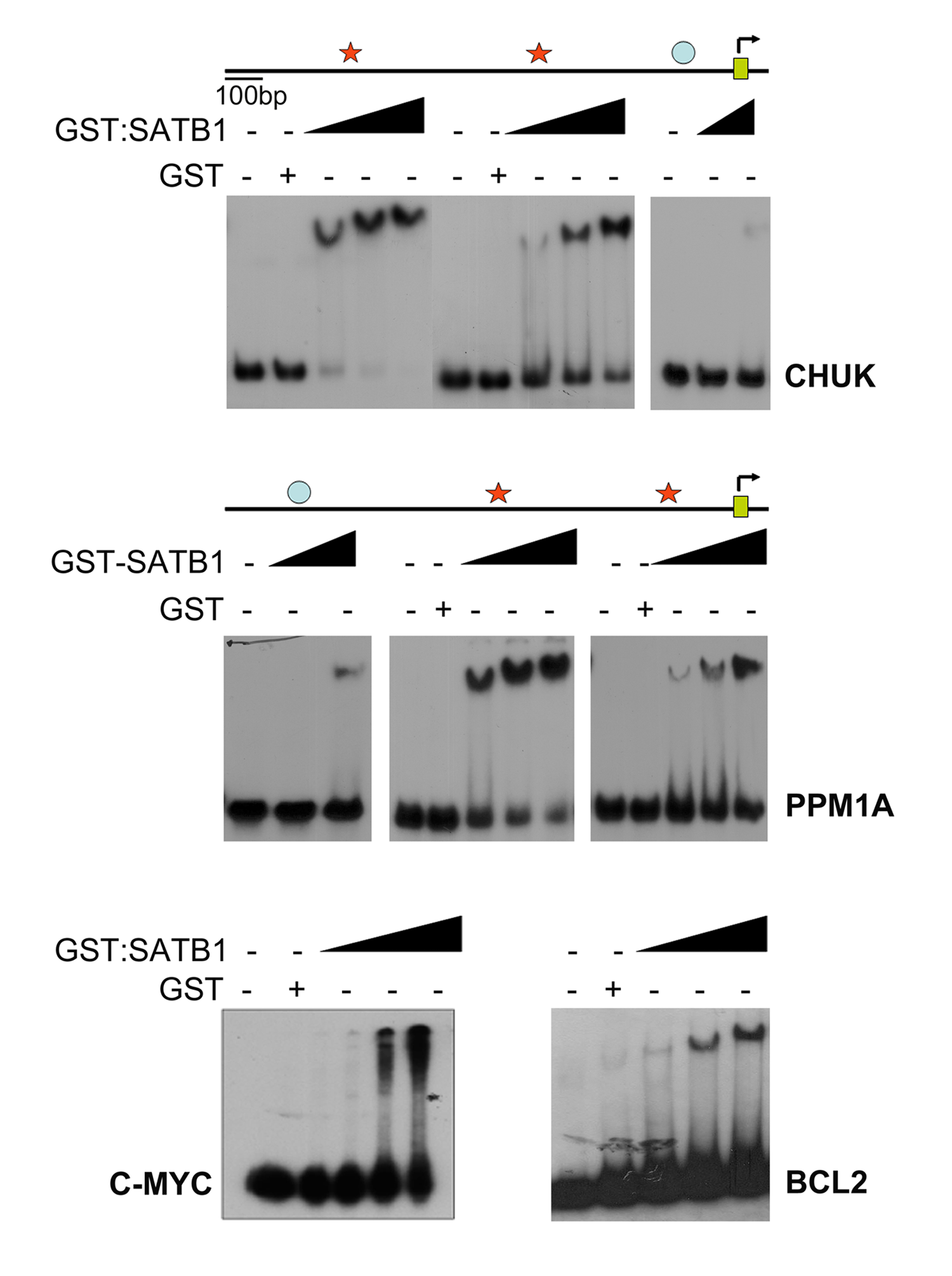

Supplement: Figure S6 — Analysis of SBSs in regulatory regions of multiple genes. EMSA analysis was performed using 32P-labeled upstream regulatory regions from CHUK, PPM1A, c-Myc genes, and the major breakpoint region of BCL2. Purified GST was used as a control for the GST:SATB1 fusion protein. The multiple regions tested from the 1 kb upstream region of CHUK and PPM1A are depicted schematically on top of the EMSA panels. Two regions (one in proximal CHUK promoter and one in distal PPM1A promoter) bound SATB1 at very high concentrations indicating very low affinity. Stars indicate SBSs whereas circles denote non-binding regions. Panel on bottom left depicts the dose-dependent binding of recombinant SATB1 with a 32P-labeled region encompassing −1,183 to −1,383 bp of human c-Myc promoter. (0.72 MB TIF) [file pbio.1000296.s006.tif]

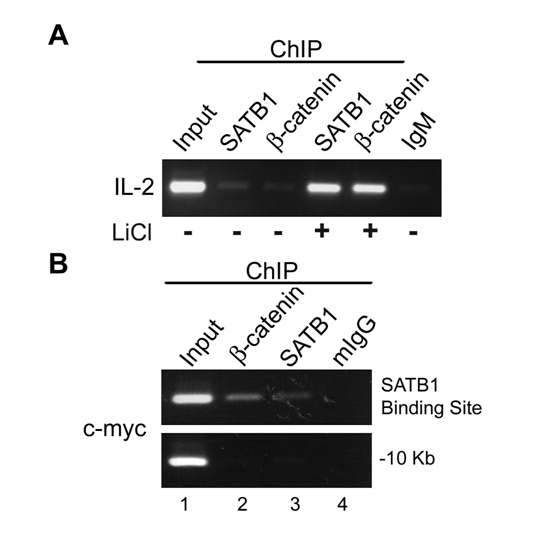

Supplement: Figure S7 — SATB1 and β-catenin occupy the SBSs in IL-2 and c-Myc promoters in vivo. (A) ChIP-PCR analysis in Jurkat cells showing the binding of SATB1 and β-catenin on IL-2 promoter in presence (+) and absence (−) of LiCl. ChIP analysis was performed as described in Materials and Methods. LiCl treatment resulted in increased occupancy of SATB1 at IL-2 promoter SBS, which then presumably recruits β-catenin at this locus. Antibodies used for ChIP are indicated on top of lanes. (B) ChIP-PCR analysis of Jurkat cells treated with Wnt agonist BIO was performed as described in Materials and Methods. SATB1 and β-catenin both occupy the SBS of human c-Myc promoter under these conditions (top panel, lanes 2 and 3). An upstream region (−10 kb) of c-Myc was used as a negative control for ChIP (lower panel). (0.11 MB TIF) [file pbio.1000296.s007.tif]

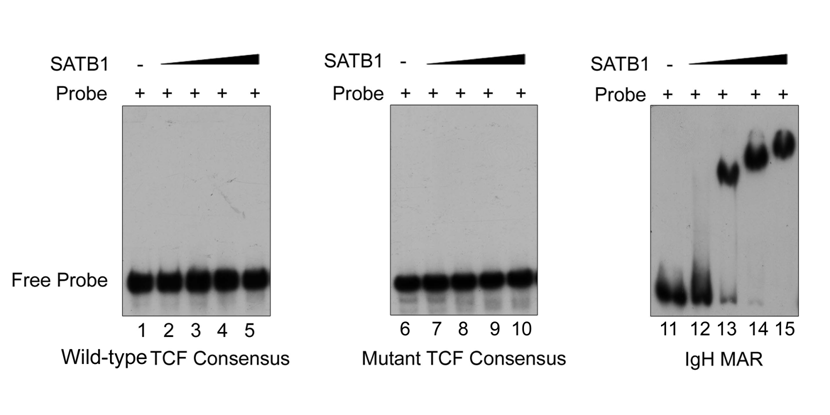

Supplement: Figure S8 — SATB1 does not bind to the TCF consensus in vitro. In vitro binding analysis of TCF consensus binding site using recombinant SATB1 was performed by EMSA as described in Materials and Methods. The various panels depict EMSA using 32P-labeled wild-type (lanes 1–5) and mutant TCF (lanes 6–10) consensus sequences. 32P-labeled IgH-MAR was used as a positive control for SATB1 binding (lanes 11–15). (0.18 MB TIF) [file pbio.1000296.s008.tif]

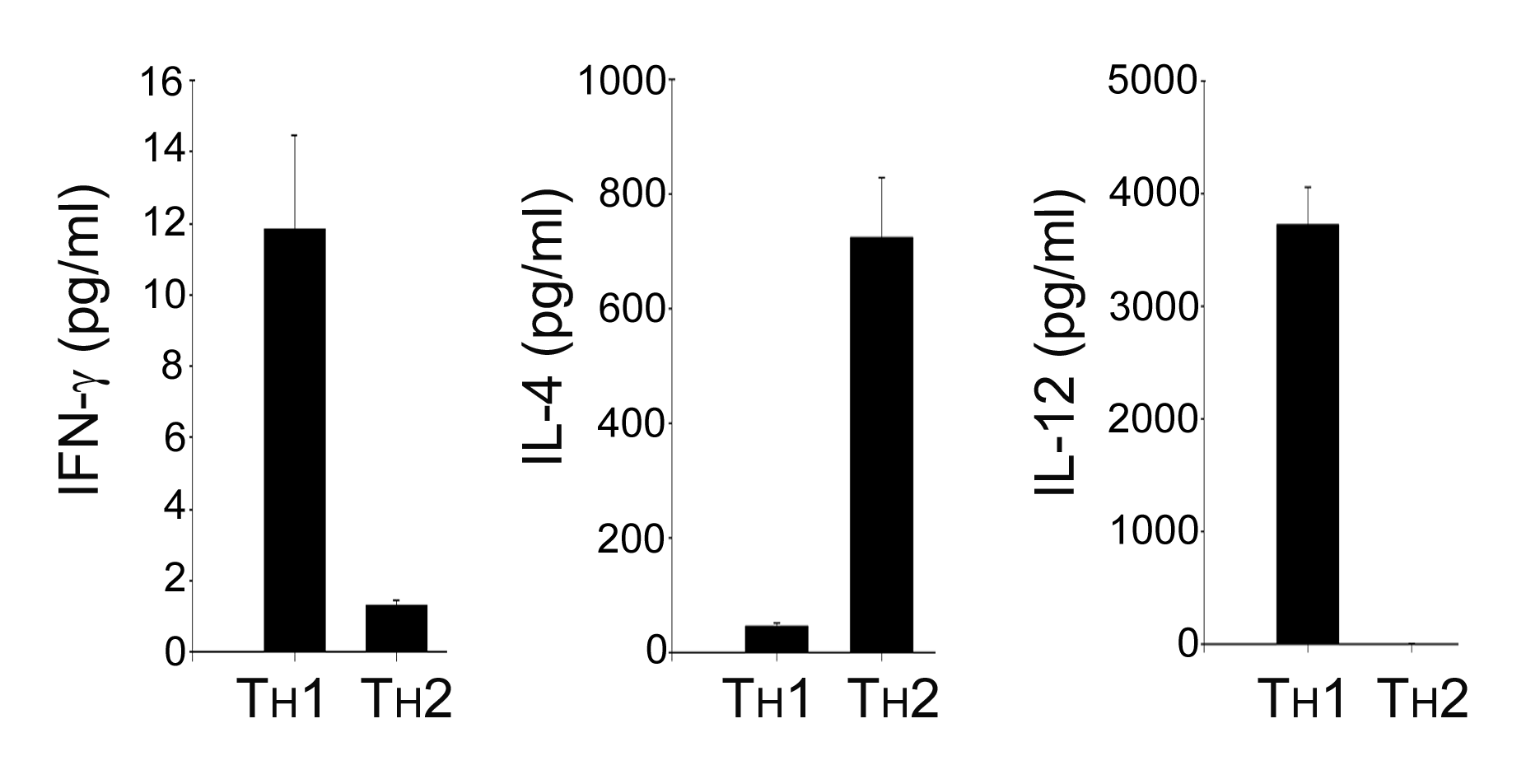

Supplement: Figure S9 — Confirmation of polarization of CD4+ T cells to TH1 and TH2 subsets. Quantitation of marker cytokines in culture supernatants harvested from TH cells grown for 72 h was performed simultaneously using a multiplex bead array reader as described in Materials and Methods. As expected, TH1 cells produced IFN-γ and IL-12 whereas TH2 cells produced IL-4, confirming that the cells were committed to the respective lineages. (5.36 MB TIF) [file pbio.1000296.s009.tif]

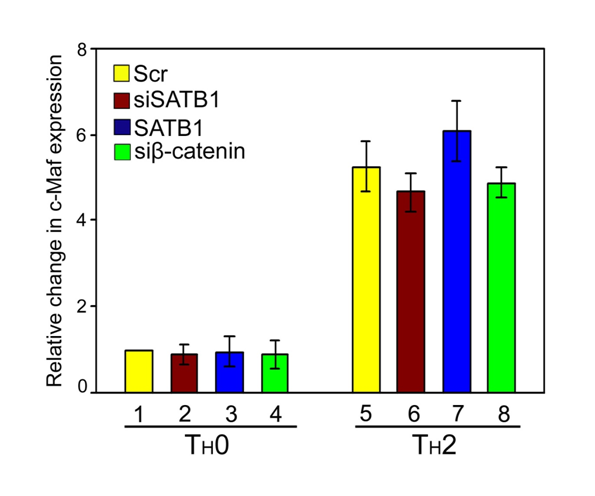

Supplement: Figure S10 — Analysis of c-Maf expression in differentiating TH cells. Naïve CD4+ T cells were transfected with duplex siSATB1, siβ-catenin, or SATB1 overexpression plasmid DNA and differentiated ex vivo as described in Materials and Methods. As the control, duplex scrambled RNA (Scr) was transfected. Upon polarization for 72 h, total RNA was isolated and GATA-3 transcripts were analyzed by quantitative RT-PCR as described in Materials and Methods. c-Maf expression was normalized with β-actin expression in these cells. The graph shows relative changes in c-Maf transcript in control (Scr) (bars 1 and 5), SATB1 silenced (bars 2 and 6), SATB1 overexpressed (bars 3 and 7), and β-catenin silenced (bars 4 and 8) TH0 (bars 1–4) and TH2 (bars 5–8) cells. Changes in expression levels were calculated with respect to the scrambled RNA transfected TH0 subset in which the c-Maf expression level was set to 1 (bar 1). Each error bar represents standard deviation calculated from triplicates. Naïve CD4+ T cells were transfected with duplex siSATB1, siβ-catenin, or SATB1 overexpression plasmid DNA as described in Materials and Methods and differentiated ex vivo as described [26]. As the control, duplex scrambled RNA (Scr) was transfected. Upon polarization for 72 h, total RNA was isolated and GATA-3 transcripts were analyzed by quantitative RT-PCR as described in Materials and Methods. GATA-3 expression was normalized with β-actin expression in these cells. The graph shows fold changes in GATA-3 transcript in control (Scr) (bar 1), SATB1 silenced (bar 2), SATB1 overexpressed (bar 3), and β-catenin silenced (bar 4) TH2 cells. Fold changes were calculated with respect to the scrambled RNA transfected TH0 subset in which the GATA-3 expression level was set to baseline (bar 1). Each error bar represents standard deviation calculated from triplicates. (0.11 MB TIF) [file pbio.1000296.s010.tif]

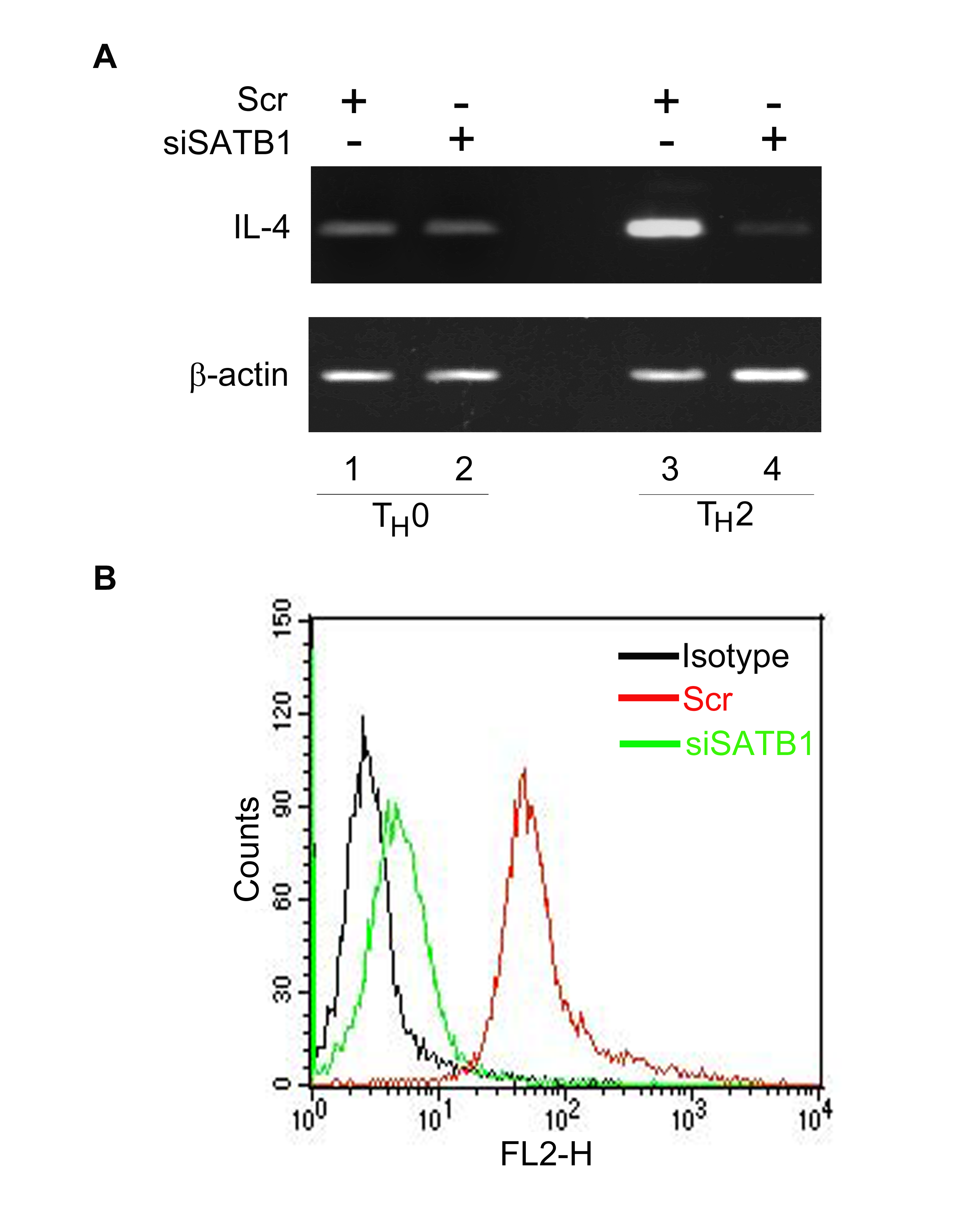

Supplement: Figure S11 — IL-4 is downregulated upon siRNA-mediated silencing of SATB1 in differentiating TH2 cells. Human CD4+ T cells were transfected with Scrambled (Scr) and si SATB1 synthetic duplex RNAs and then polarized to TH2 as described in Materials and Methods. After 72 h the cells were harvested and used for monitoring the expression of IL-4. (A) IL-4 gene expression profiling. Total RNA was isolated and IL-4 transcripts were analyzed by RT-PCR as described in Materials and Methods. GAPDH expression served as the control for amount of RNA in the RT-PCRs. (B) The CD4+ T cells were polarized under TH2 conditions and harvested after 3 d. Four h prior to harvesting the cells, Brefeldin A was added to the culture media. The cells were fixed using 1% paraformaldehyde for 20 min at room temperature. The cells were then permeabilized using 0.5% Saponin and stained with anti-IL-4-PE conjugate (BD Biosciences) for 30 min. Stained cells were acquired on flow cytometer (BD FACS Calibur) and analyzed using CellQuest software (BD Biosciences). (1.96 MB TIF) [file pbio.1000296.s011.tif]

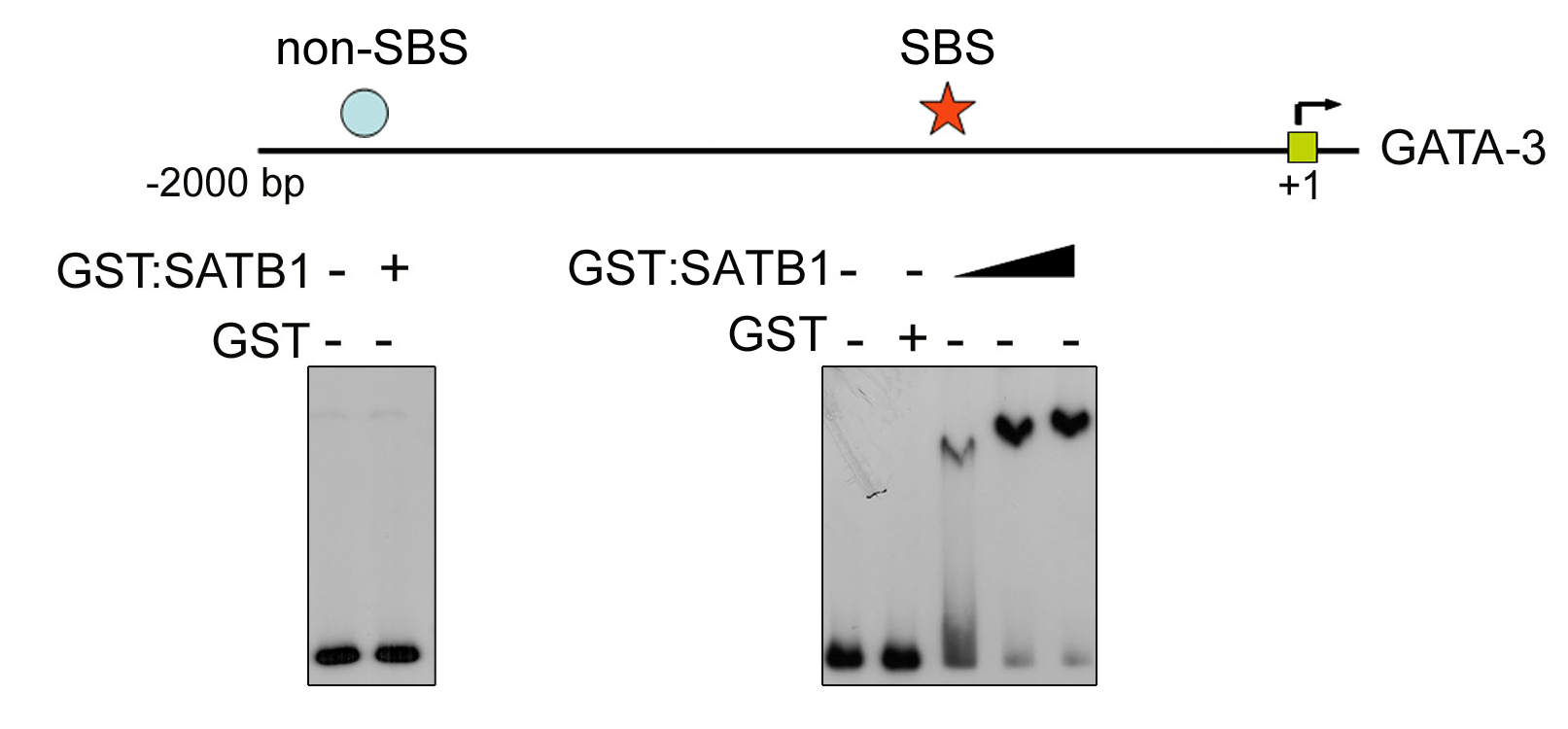

Supplement: Figure S12 — Analysis of upstream regulatory regions of GATA-3 . In vitro binding analysis of GATA-3 promoter using recombinant SATB1 was performed by EMSA as described in Materials and Methods. The binding of SATB1 across the 2 kb (+1 to −2,000 bp) upstream regulatory region of GATA-3 was monitored by using 32P-labeled fragments of this region. We focused on a high affinity SBS at a region spanning −900 to −600 bp and a non-SBS at an upstream region encompassing −1,800 to −1,500 bp of the human GATA-3 promoter. The schematic on top of the EMSA panels depicts the relative positions of these regions within the promoter. A star indicates position of SBS, whereas a circle denotes non-binding site. (0.25 MB TIF) [file pbio.1000296.s012.tif]
