# Supplementary material for: Global Regulator SATB1 Recruits β-Catenin and Regulates TH2 Differentiation in Wnt-Dependent Manner
Source: PLoS Biol. 2010 Jan 26;8(1):e1000296. doi: 10.1371/journal.pbio.1000296 (PMC2811152; doi:10.1371/journal.pbio.1000296)
Supplement: Table S1 — List of genes and gene IDs. The genes studied here or mentioned in the text are tabulated along with their respective gene IDs. (0.05 MB DOC) [file pbio.1000296.s013.doc]

**Supplementary table 1**

| **Gene** | **Gene ID (NCBI)** |
| --- | --- |
| β Catenin | 1499 |
| Bcl2 | 596 |
| Bcl-xl | 598 |
| CHUK | 1147 |
| cMAf | 17132 |
| cMyc | 17869 |
| ERBB2 | 2064 |
| FOSB | 2354 |
| GATA3 (Hu) | 2625 |
| GATA3 (ms) | 14462 |
| GSK3 β | 2932 |
| IFNγ | 3458 |
| IL10 | 3586 |
| IL12 | 3592 |
| IL13 | 3596 |
| IL2 | 3558 |
| IL4 | 3565 |
| p300 | 2033 |
| PCAF | 8850 |
| PP2A | 55241 |
| SATB1 (Hu) | 6304 |
| SATB1 (ms) | 20230 |
| STAT4 | 20849 |
| STAT6 | 20852 |
| T-bet | 57765 |
| TCF1 | 51176 |
| TCF4 | 6925 |
| ThPOK | 51043 |
| TIMP1 | 7076 |
| TRAIL | 8743 |
