# Supplementary material for: Expression of Concern: Global Regulator SATB1 Recruits β-Catenin and Regulates TH2 Differentiation in Wnt-Dependent Manner
Source: PLoS Biol. 2022 Nov 23;20(11):e3001908. doi: 10.1371/journal.pbio.3001908 (PMC9683845; doi:10.1371/journal.pbio.3001908)
Supplement: S1 File — (ZIP) [file pbio.3001908.s001.zip › 6557773 Original Files/Fig 1.pptx]

## Slide 1
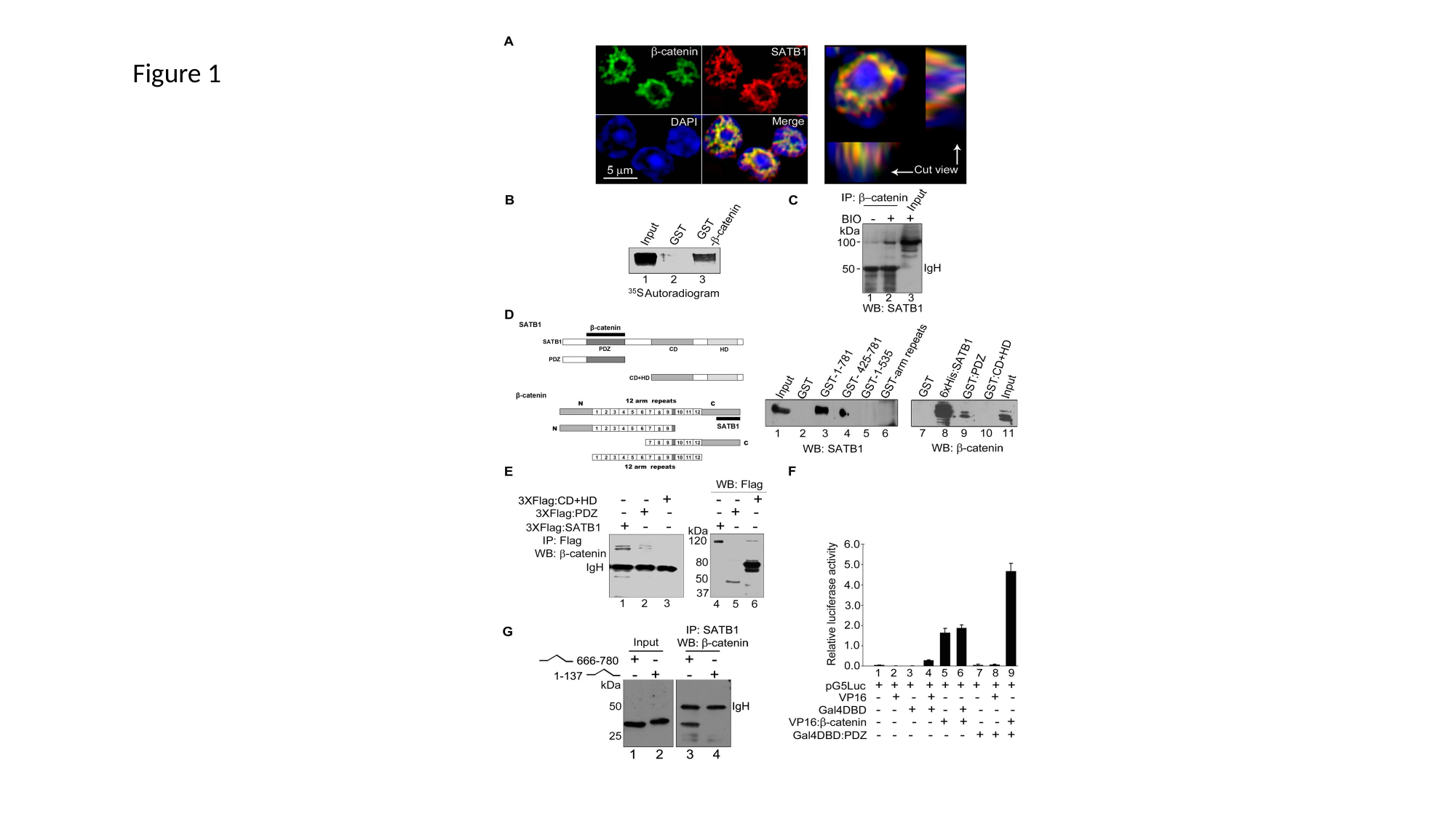

Figure 1

## Slide 2
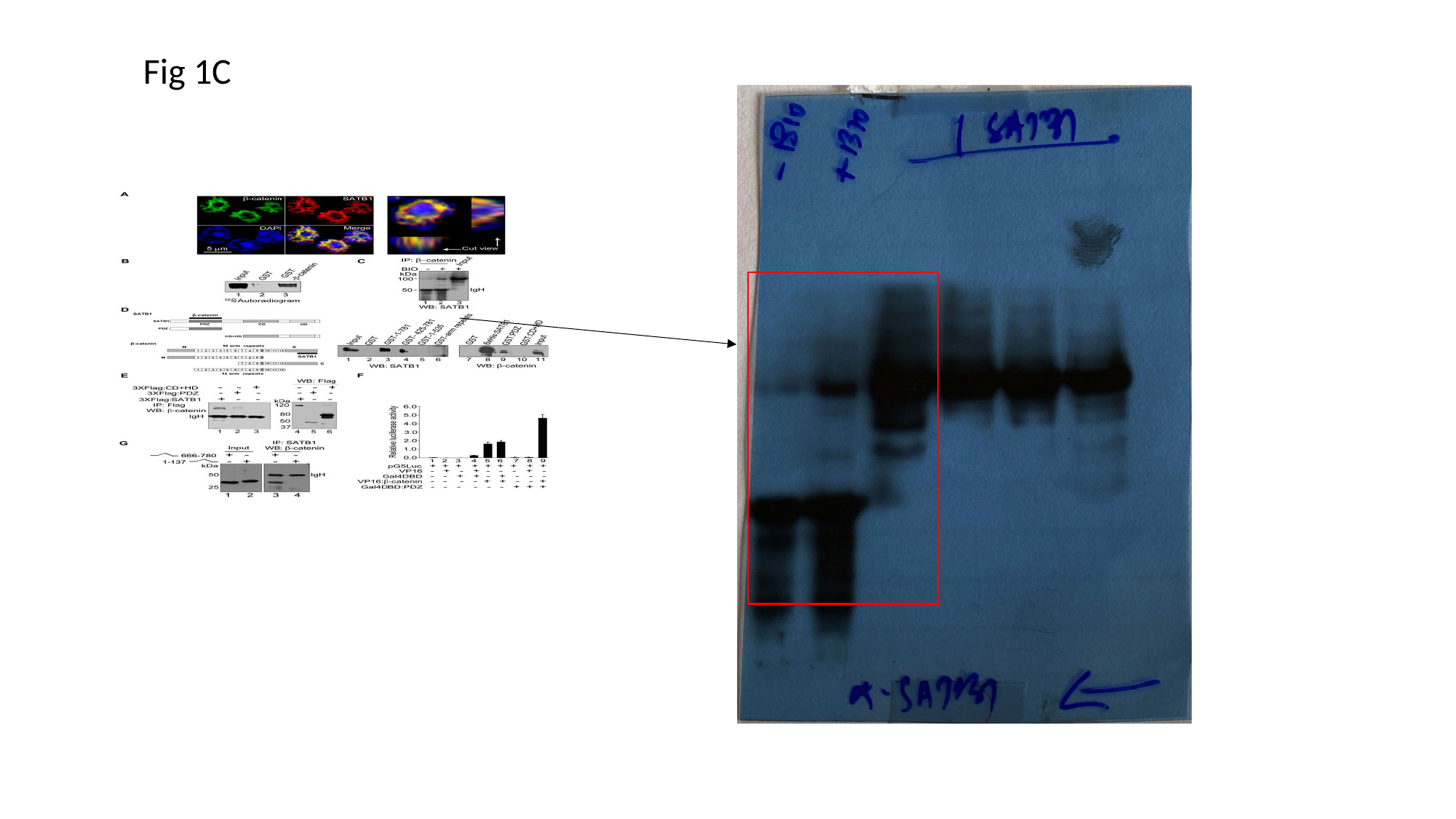

Fig 1C

## Slide 3
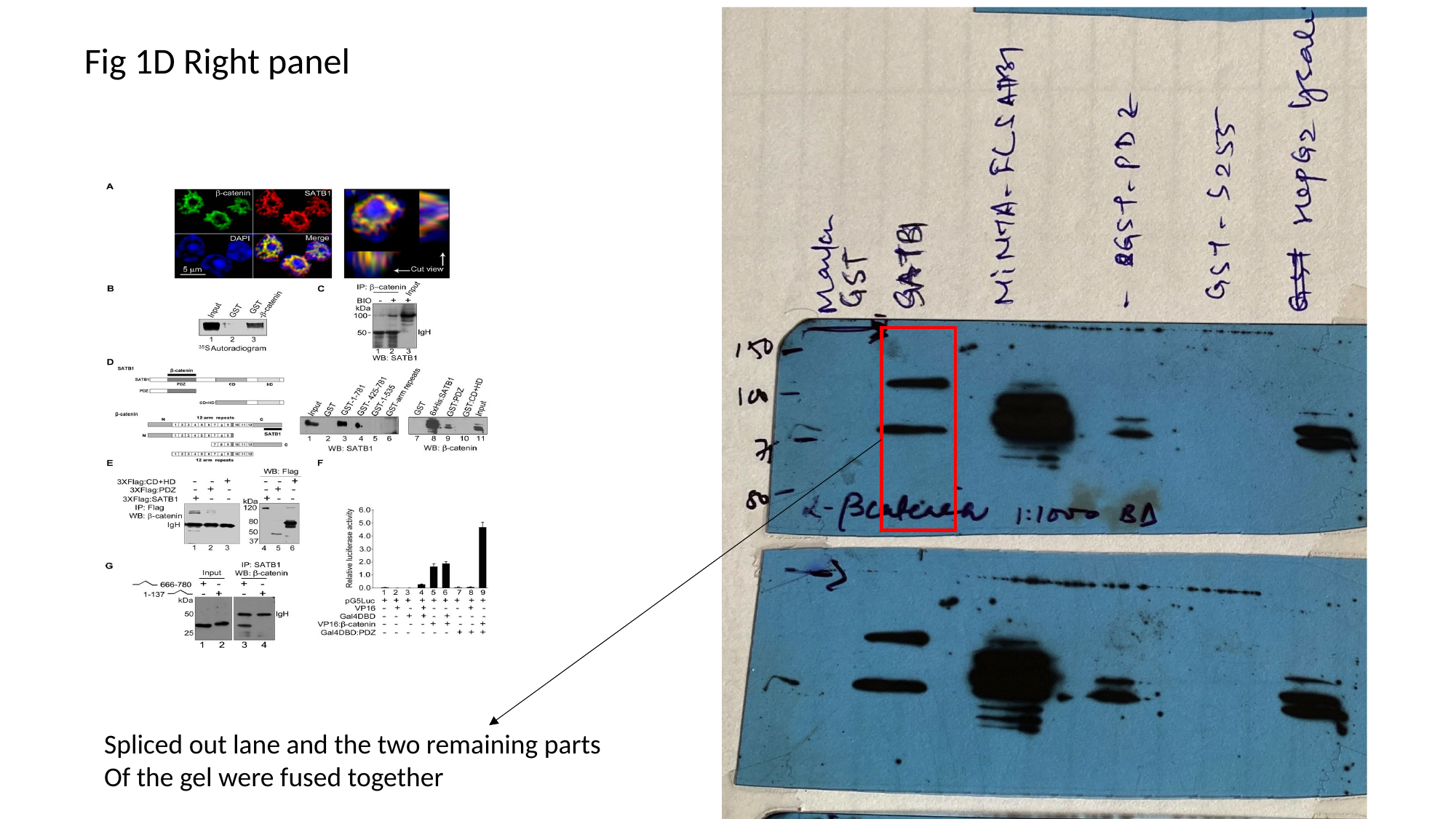

Fig 1D Right panel
Spliced out lane and the two remaining parts
Of the gel were fused together

## Slide 4
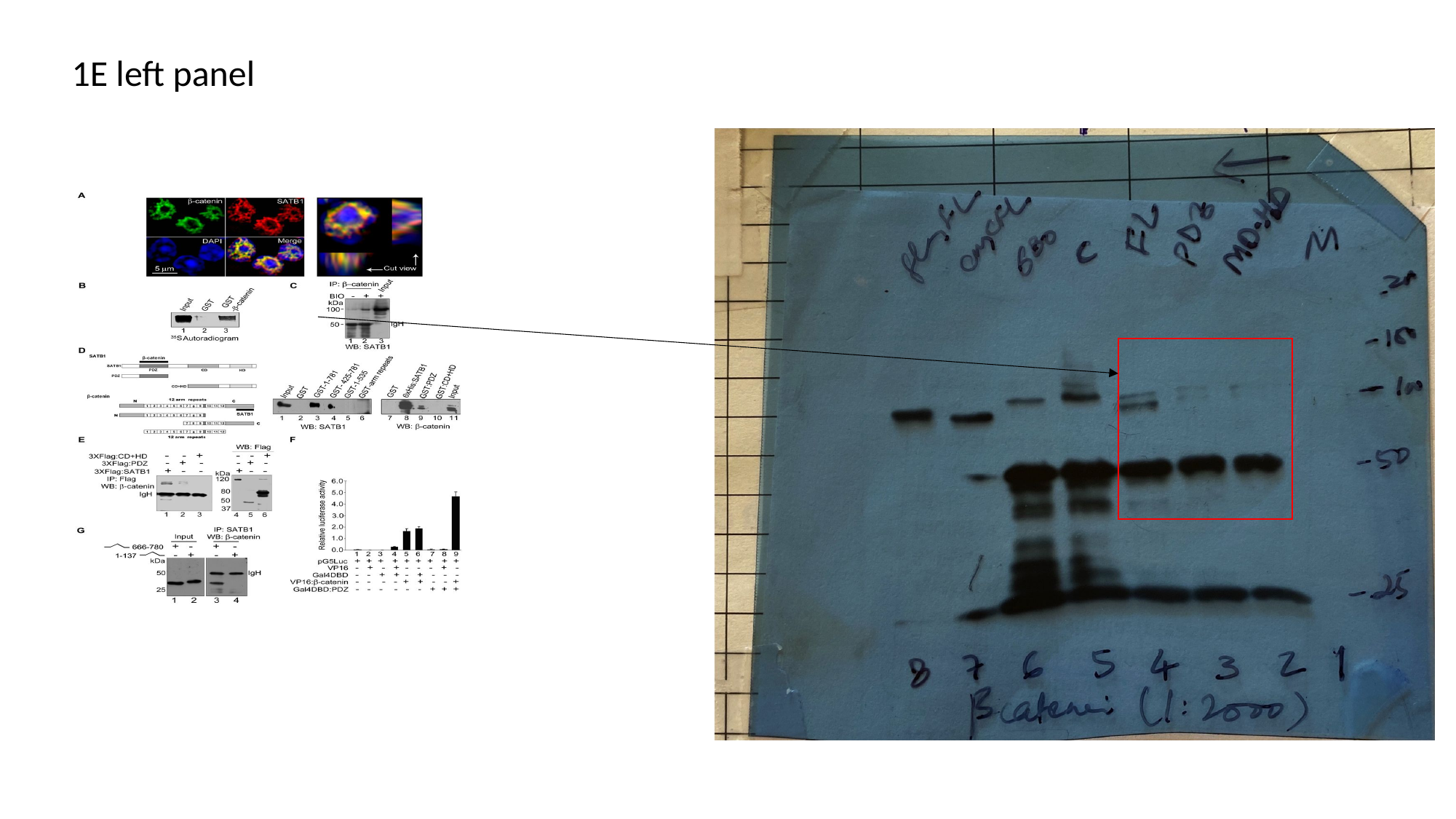

1E left panel

## Slide 5
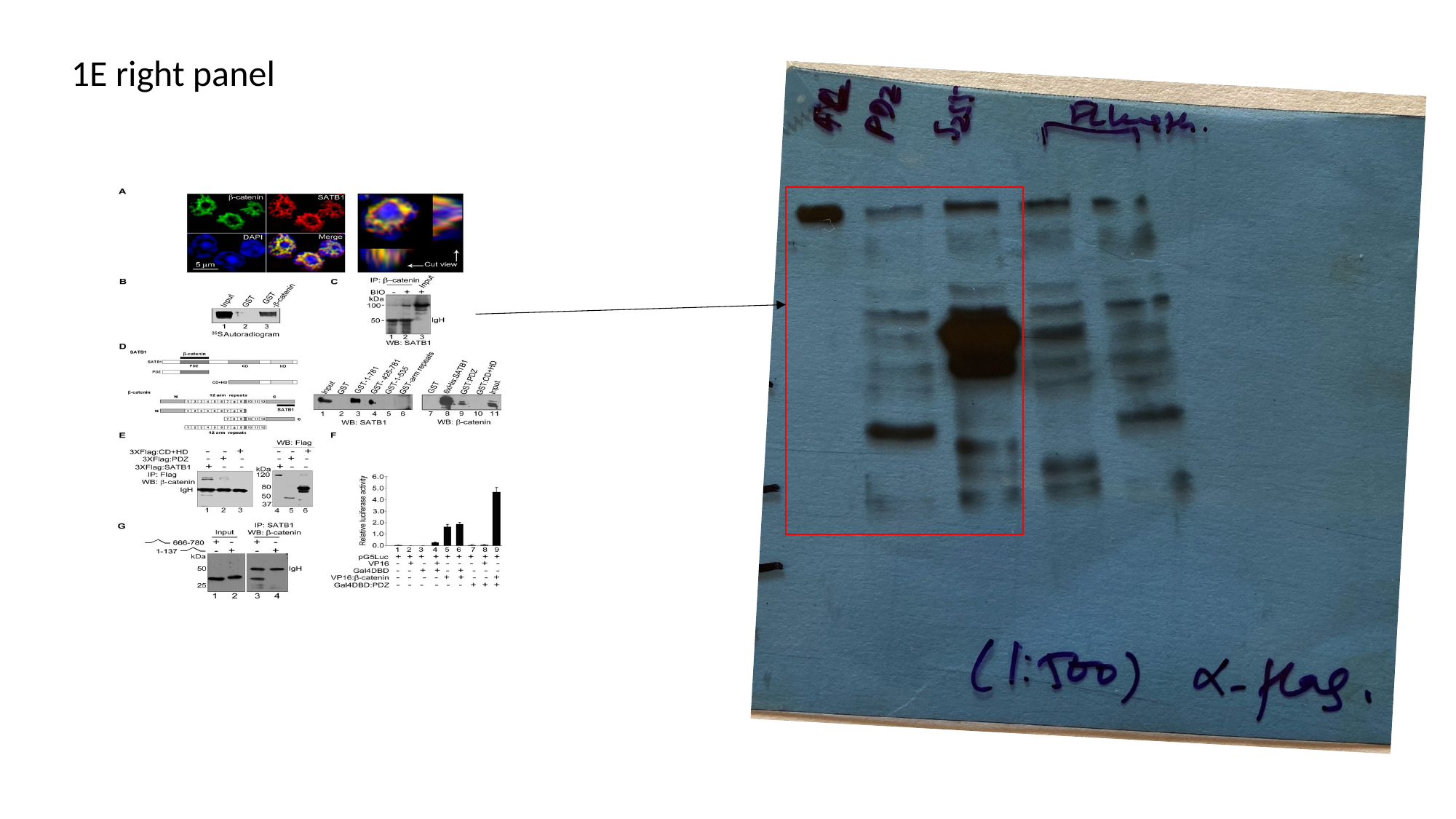

1E right panel

## Slide 6
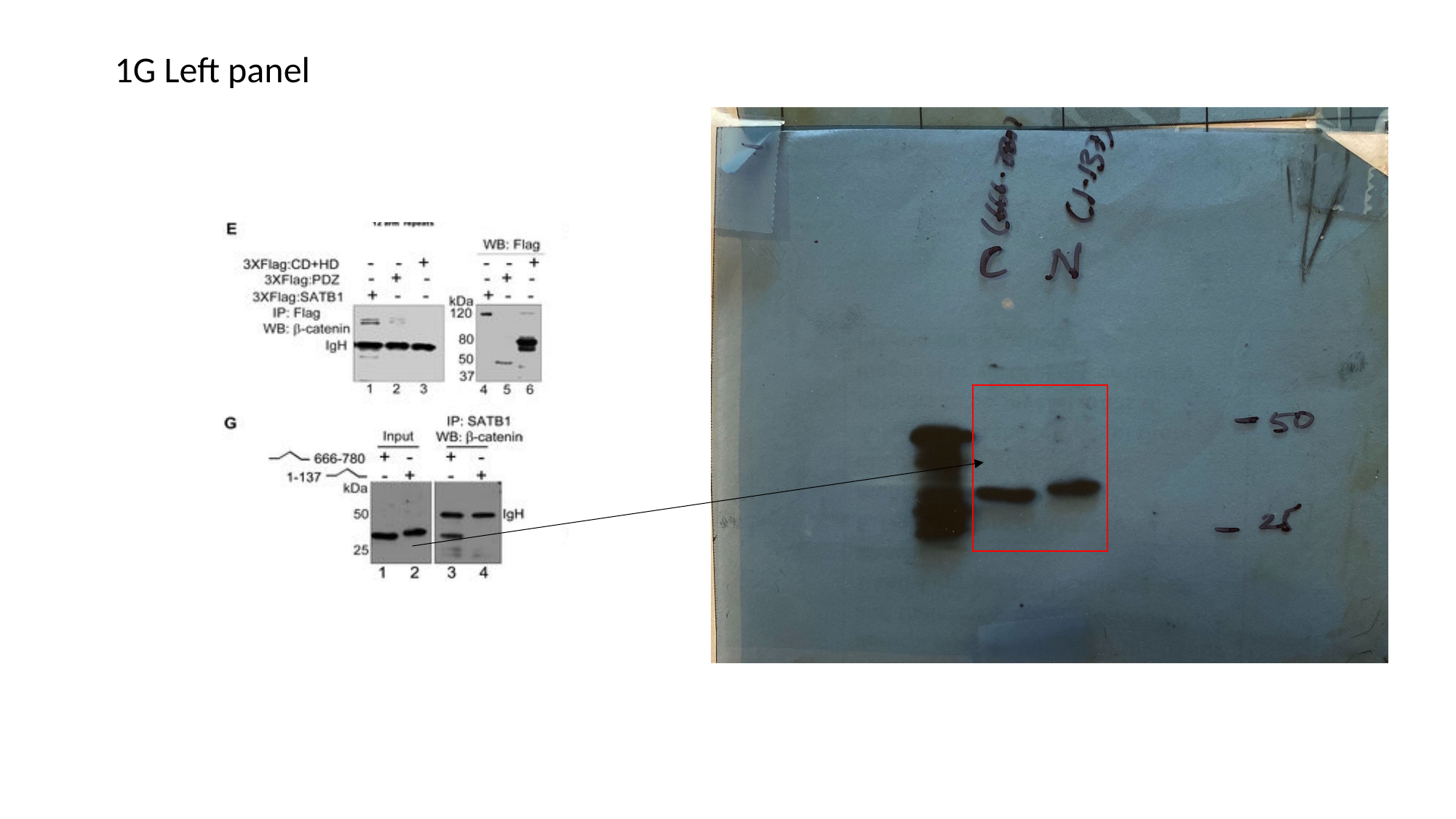

1G Left panel

## Slide 7
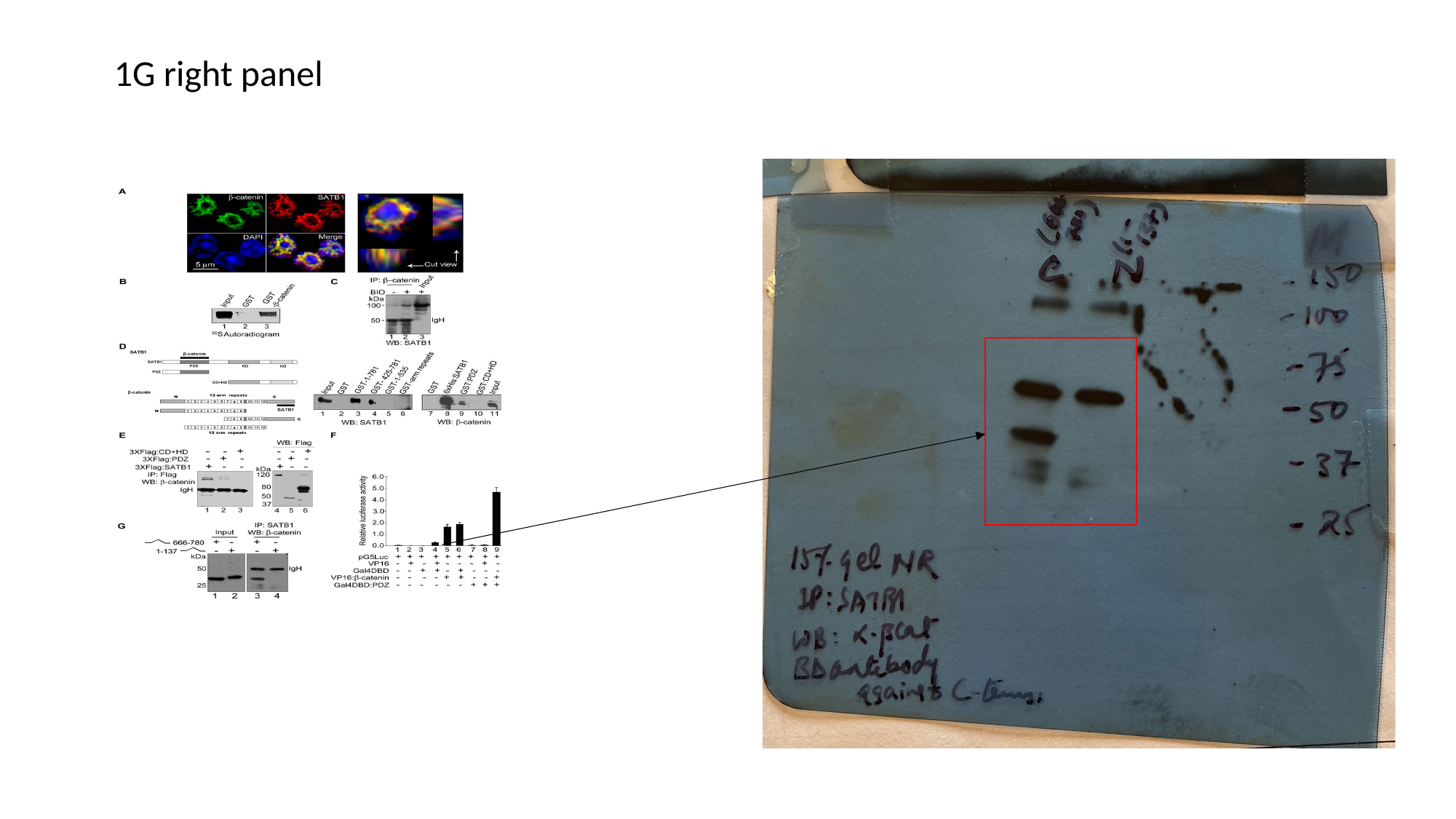

1G right panel
