# Supplementary material for: Expression of Concern: Global Regulator SATB1 Recruits β-Catenin and Regulates TH2 Differentiation in Wnt-Dependent Manner
Source: PLoS Biol. 2022 Nov 23;20(11):e3001908. doi: 10.1371/journal.pbio.3001908 (PMC9683845; doi:10.1371/journal.pbio.3001908)
Supplement: S1 File — (ZIP) [file pbio.3001908.s001.zip › 6557773 Original Files/Fig 5.pptx]

## Slide 1
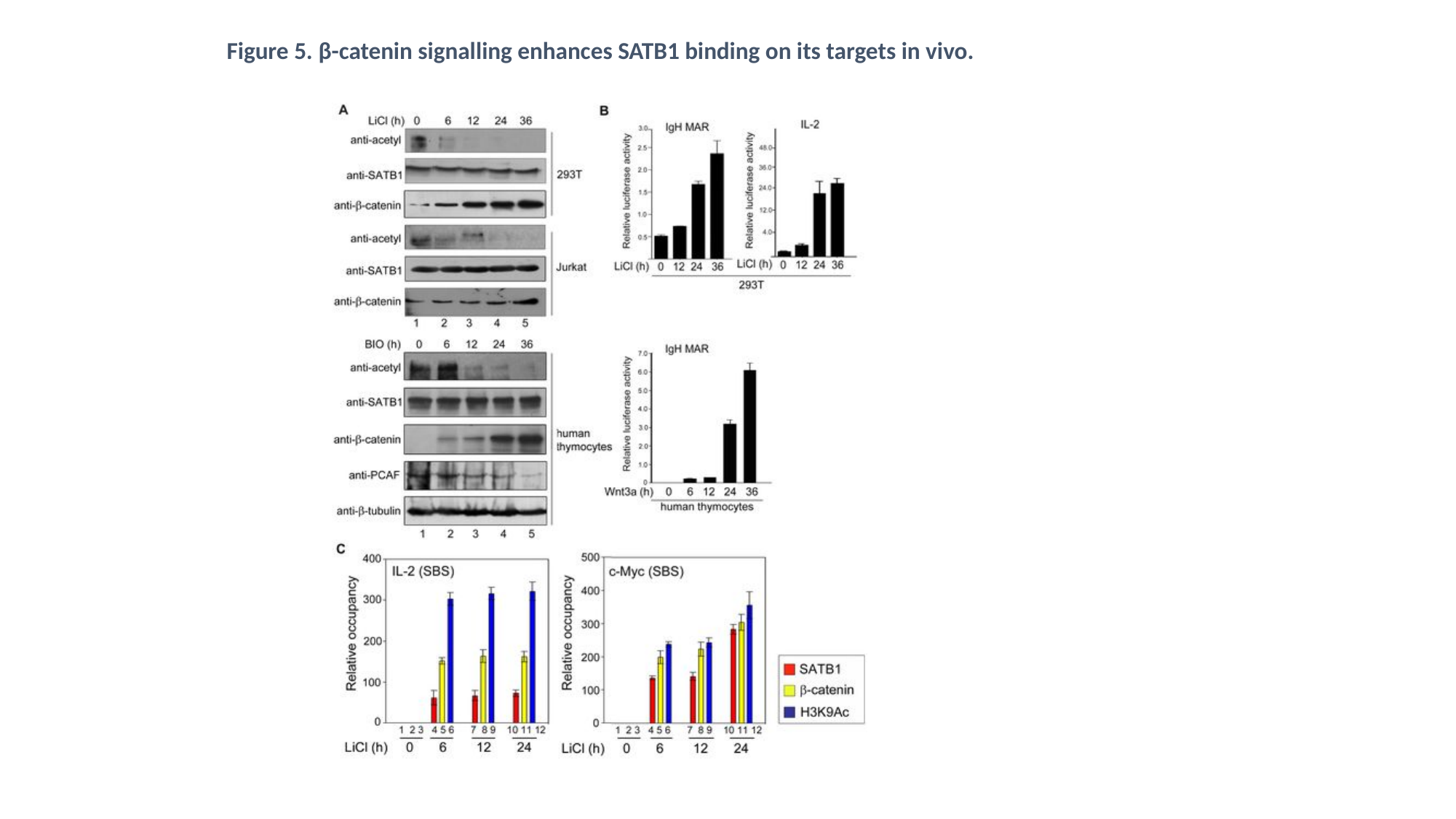

Figure 5. β-catenin signalling enhances SATB1 binding on its targets in vivo.

## Slide 2
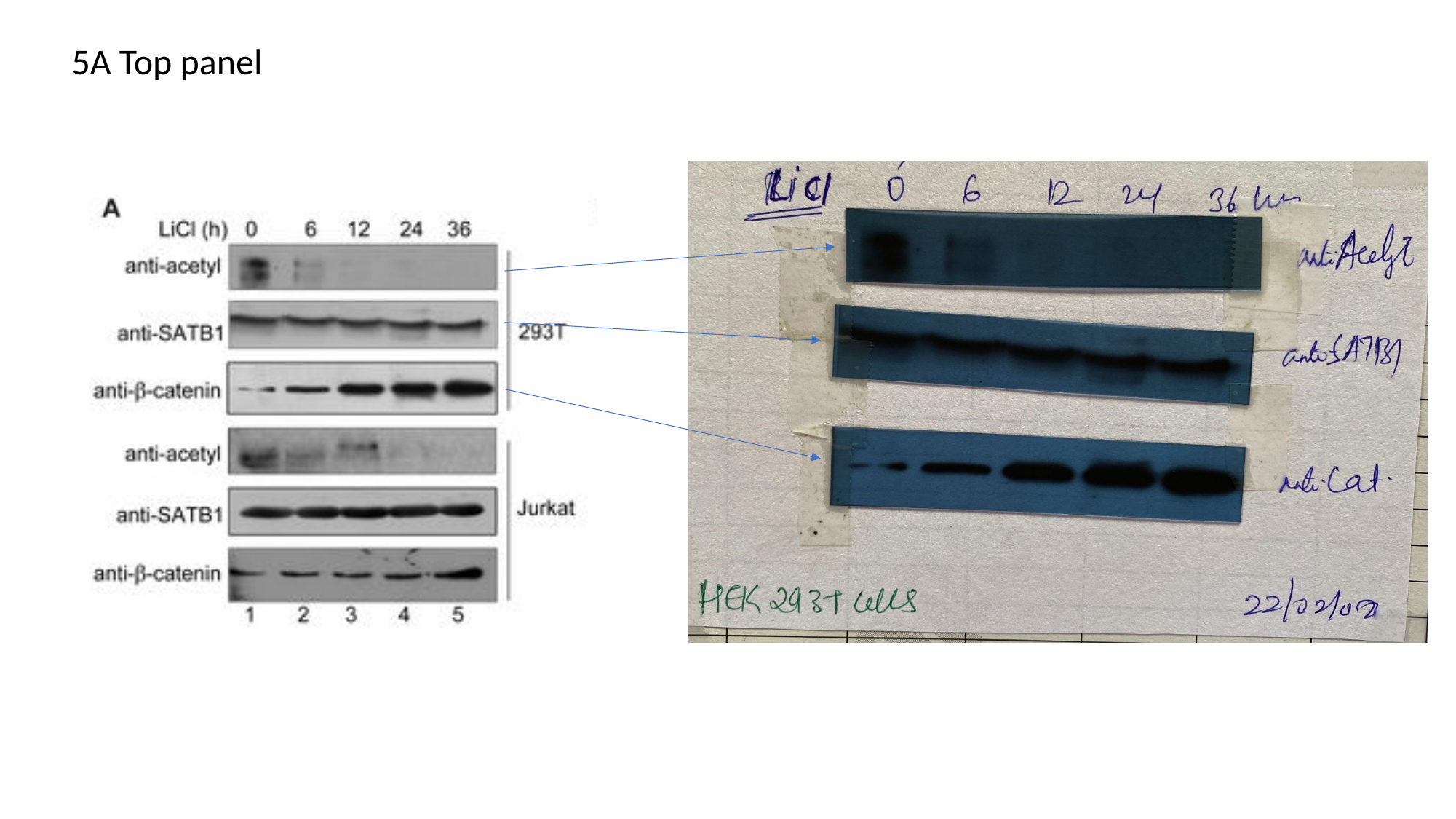

5A Top panel

## Slide 3
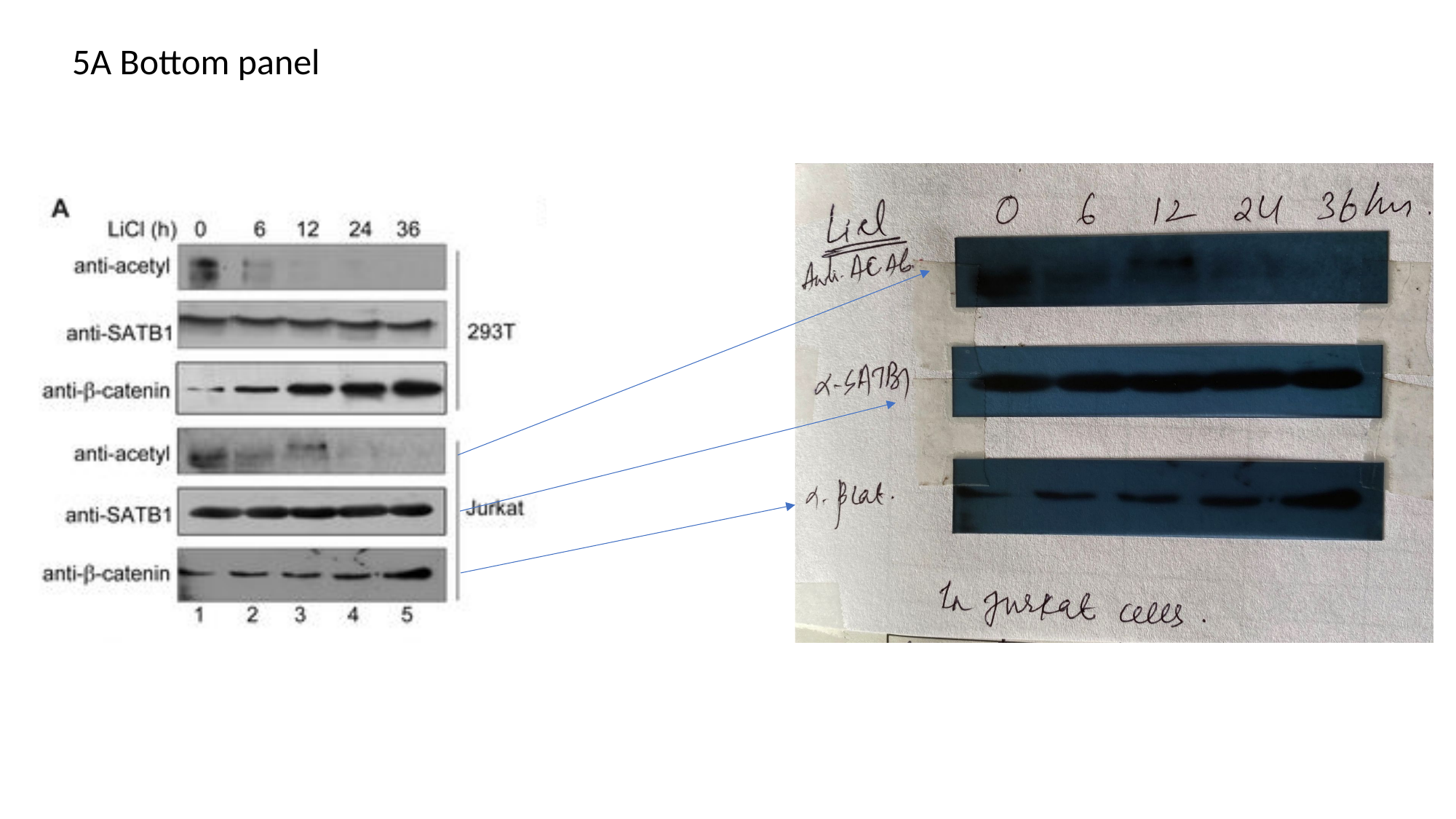

5A Bottom panel

## Slide 4
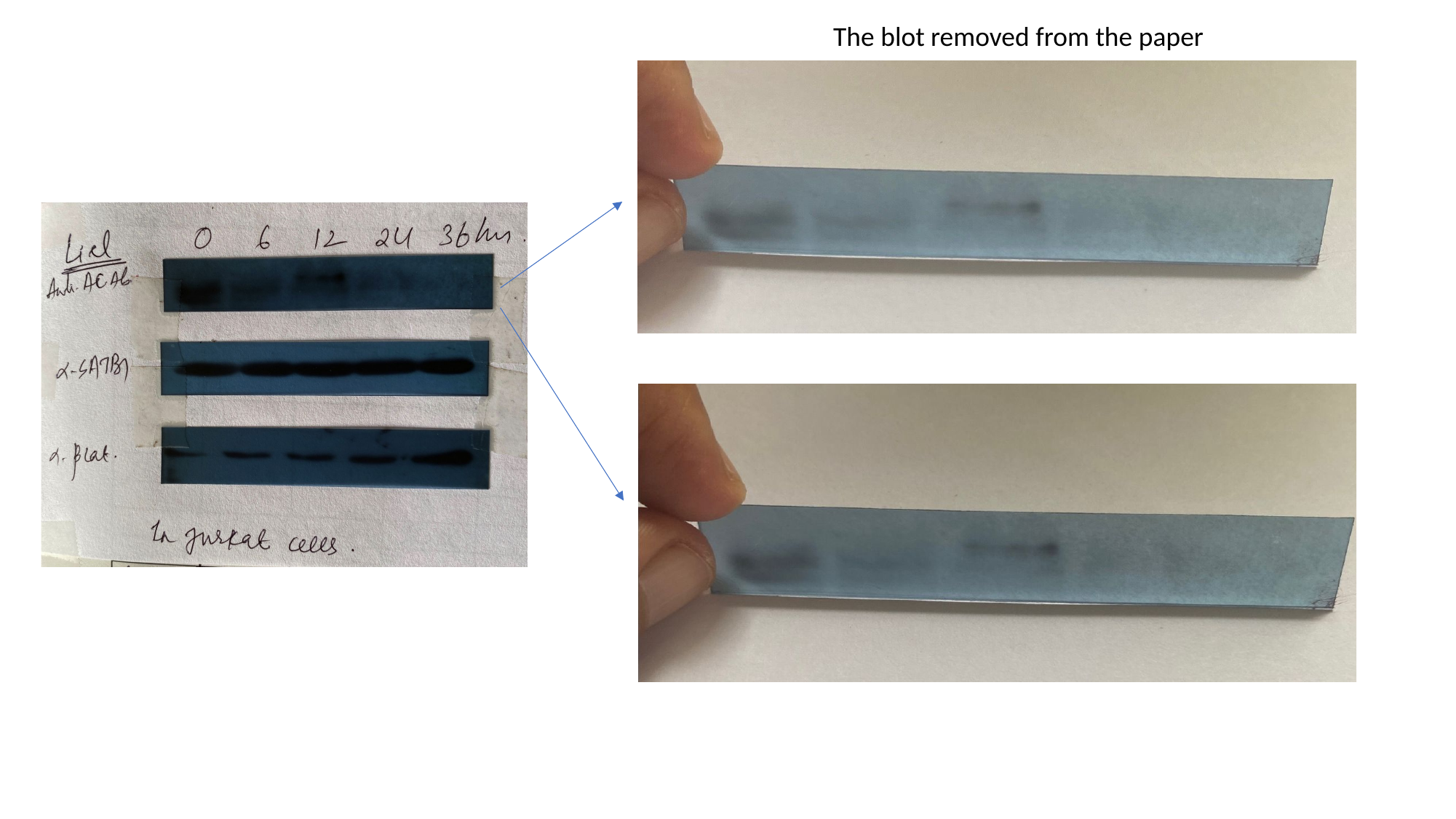

The blot removed from the paper

## Slide 5
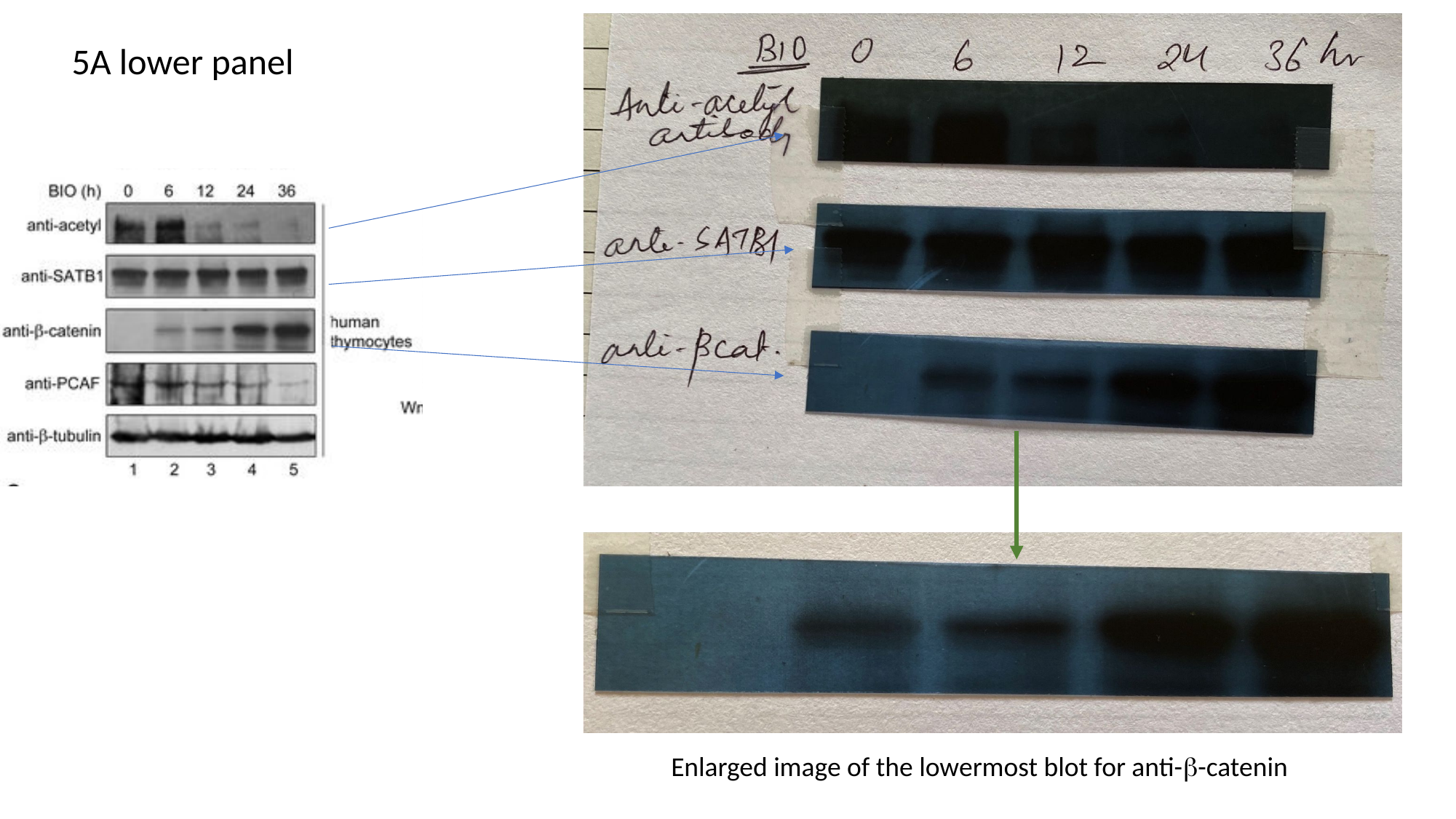

5A lower panel
Enlarged image of the lowermost blot for anti-b-catenin

## Slide 6
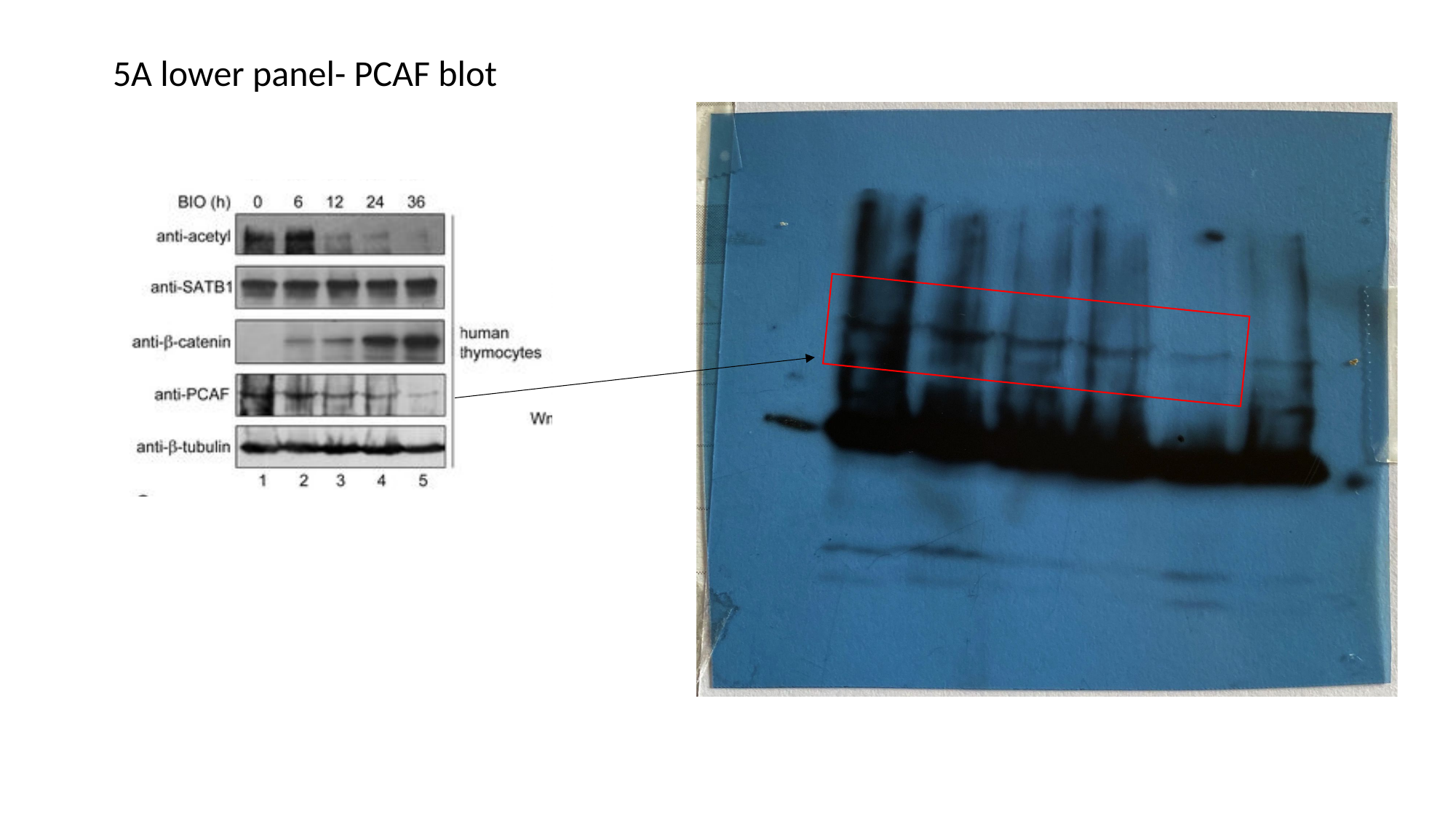

5A lower panel- PCAF blot

## Slide 7
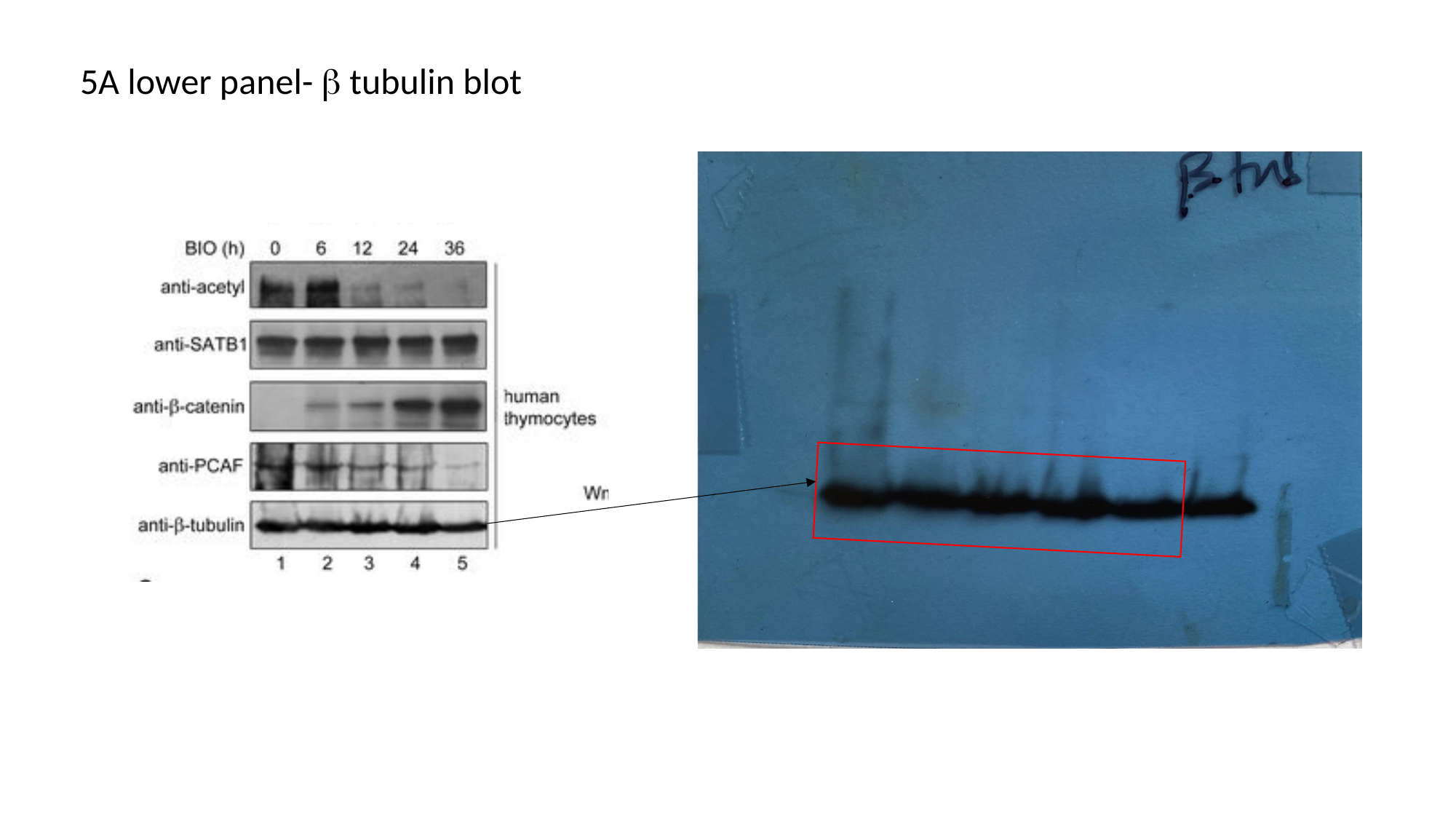

5A lower panel- b tubulin blot
