# Supplementary material for: Expression of Concern: Global Regulator SATB1 Recruits β-Catenin and Regulates TH2 Differentiation in Wnt-Dependent Manner
Source: PLoS Biol. 2022 Nov 23;20(11):e3001908. doi: 10.1371/journal.pbio.3001908 (PMC9683845; doi:10.1371/journal.pbio.3001908)
Supplement: S1 File — (ZIP) [file pbio.3001908.s001.zip › 6557773 Original Files/Fig 7.pptx]

## Slide 1
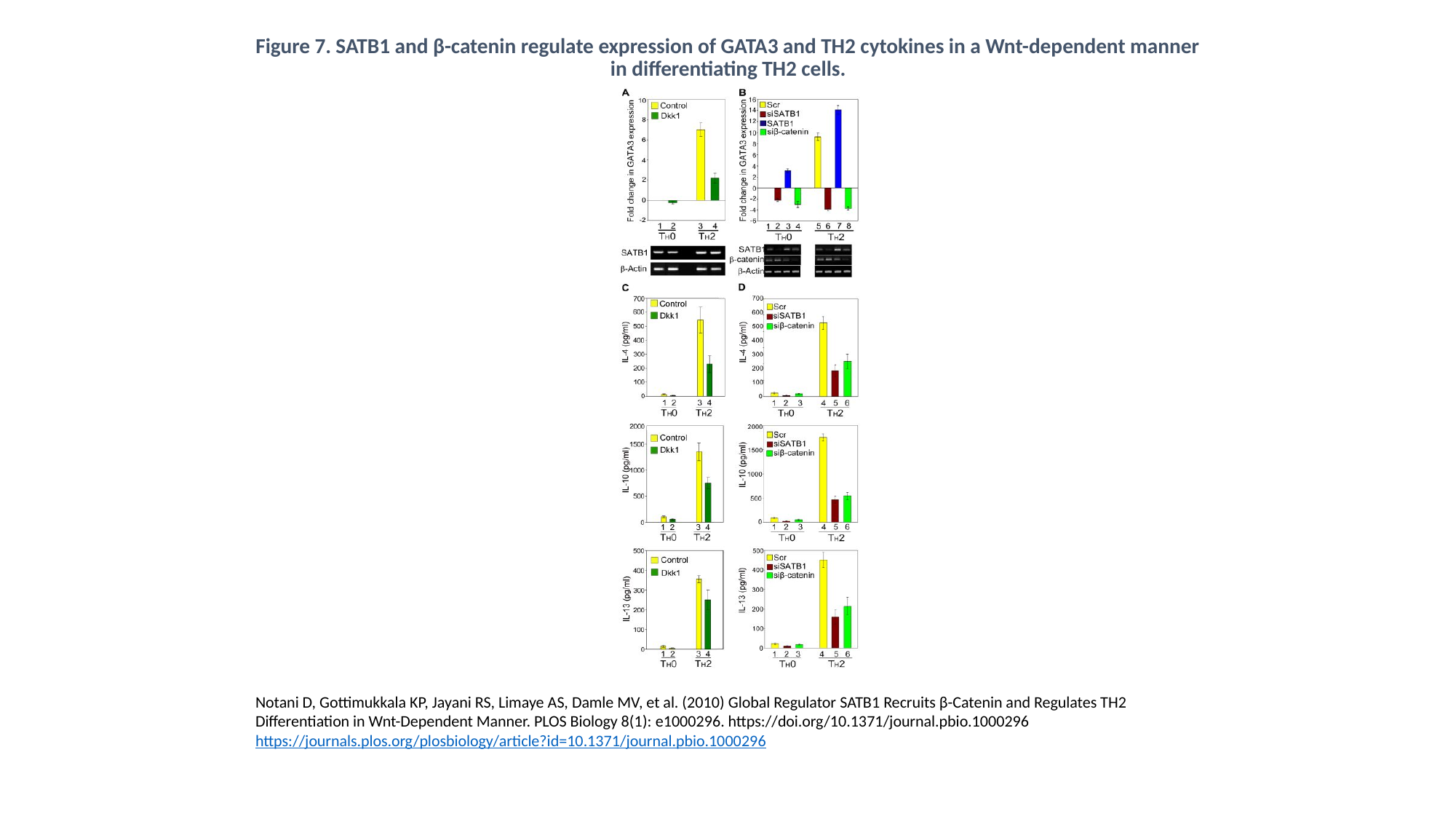

Figure 7. SATB1 and β-catenin regulate expression of GATA3 and TH2 cytokines in a Wnt-dependent manner in differentiating TH2 cells.
Notani D, Gottimukkala KP, Jayani RS, Limaye AS, Damle MV, et al. (2010) Global Regulator SATB1 Recruits β-Catenin and Regulates TH2 Differentiation in Wnt-Dependent Manner. PLOS Biology 8(1): e1000296. https://doi.org/10.1371/journal.pbio.1000296
https://journals.plos.org/plosbiology/article?id=10.1371/journal.pbio.1000296

## Slide 2
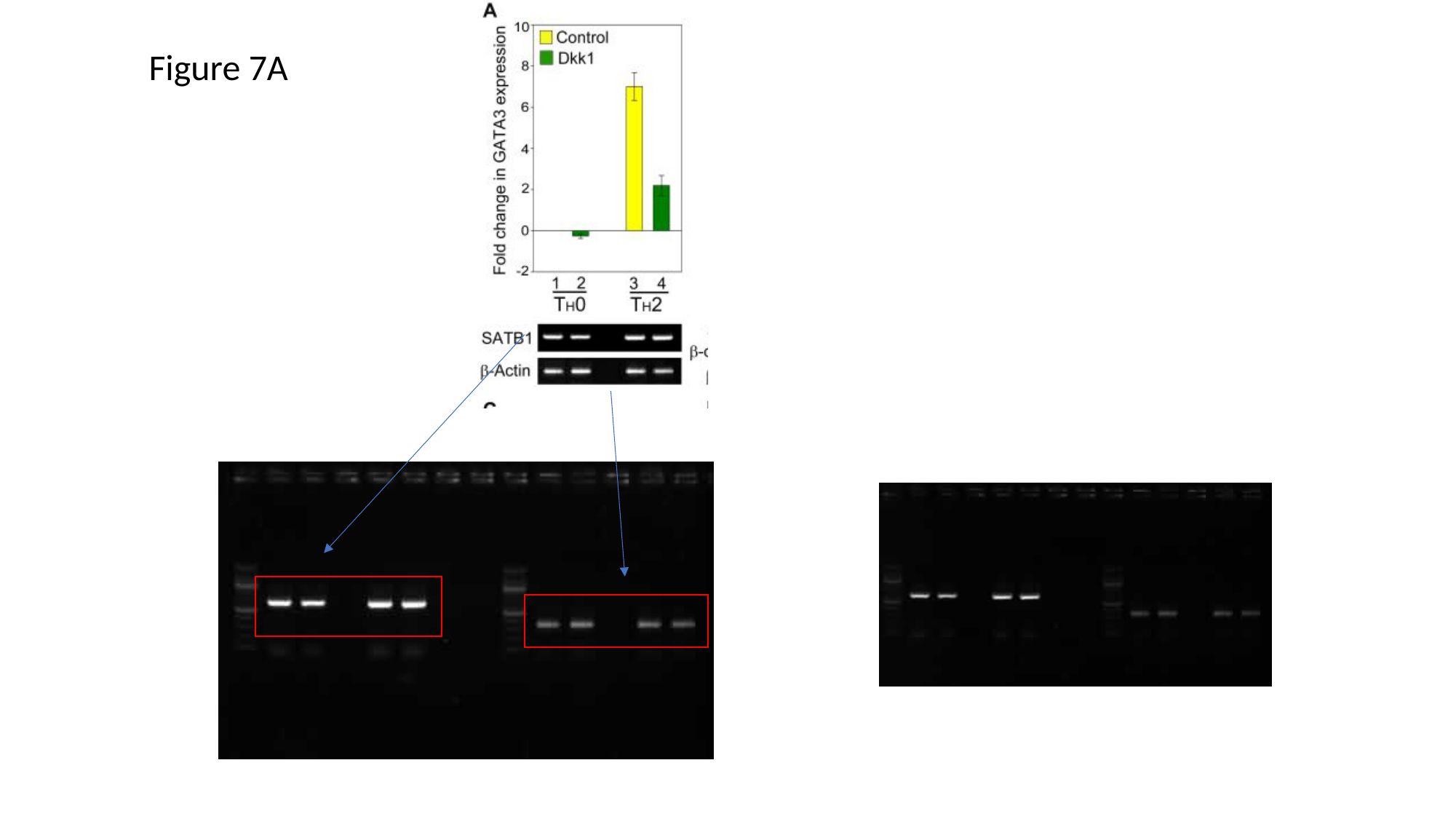

Figure 7A

## Slide 3
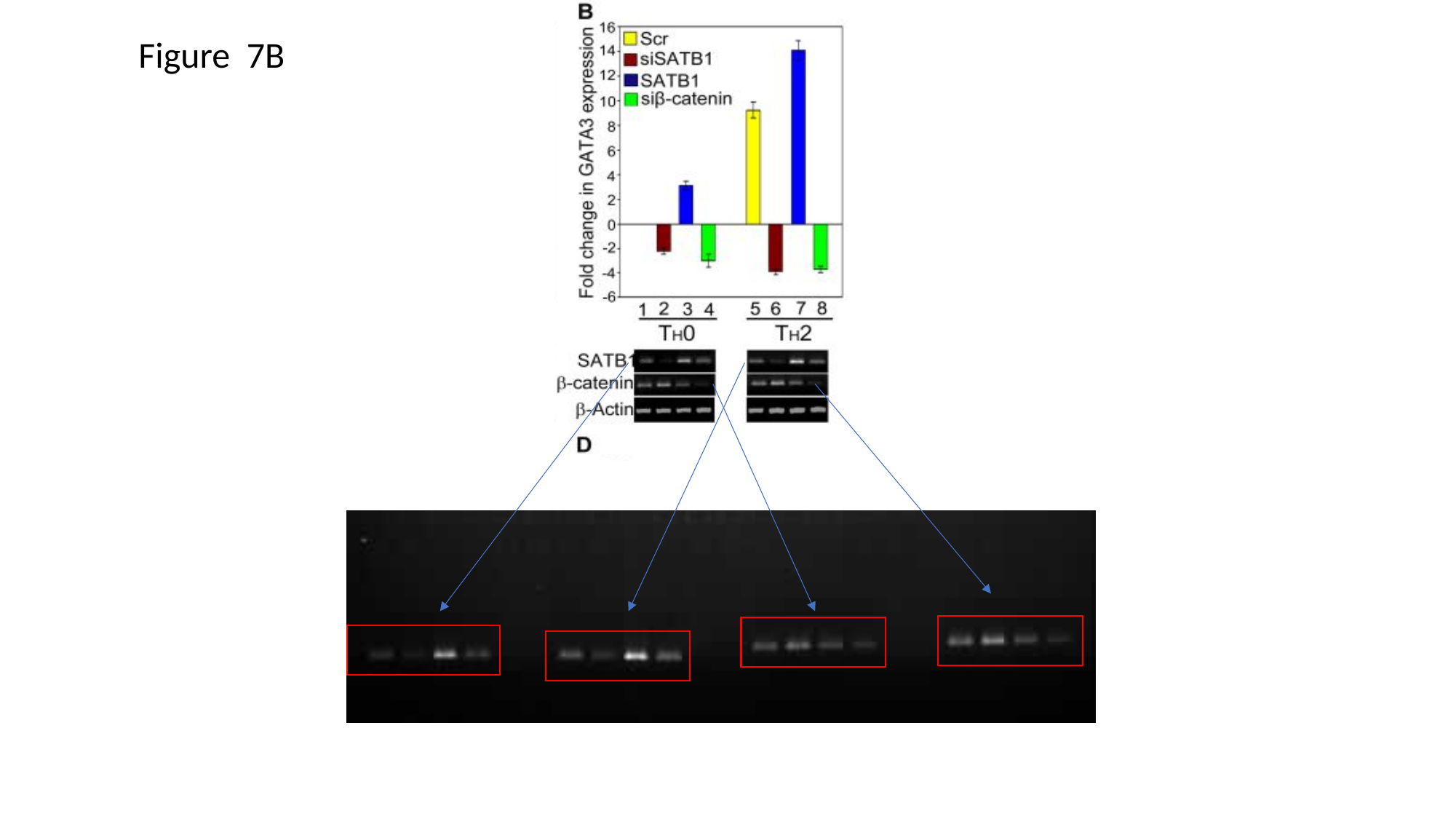

Figure 7B
