# Supplementary material for: Expression of Concern: Global Regulator SATB1 Recruits β-Catenin and Regulates TH2 Differentiation in Wnt-Dependent Manner
Source: PLoS Biol. 2022 Nov 23;20(11):e3001908. doi: 10.1371/journal.pbio.3001908 (PMC9683845; doi:10.1371/journal.pbio.3001908)
Supplement: S1 File — (ZIP) [file pbio.3001908.s001.zip › 6557773 Original Files/Fig S2.pptx]

## Slide 1
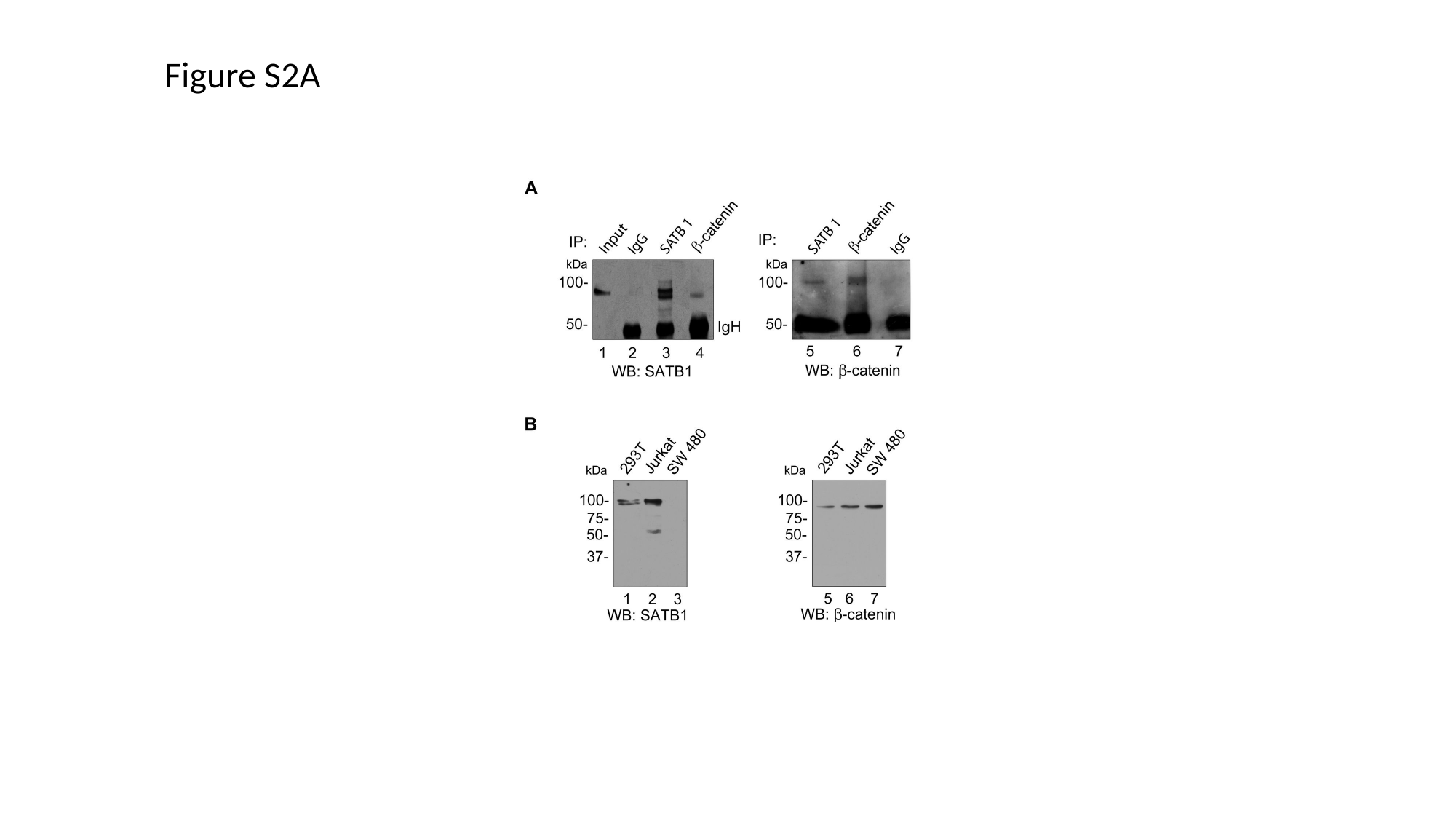

Figure S2A

## Slide 2
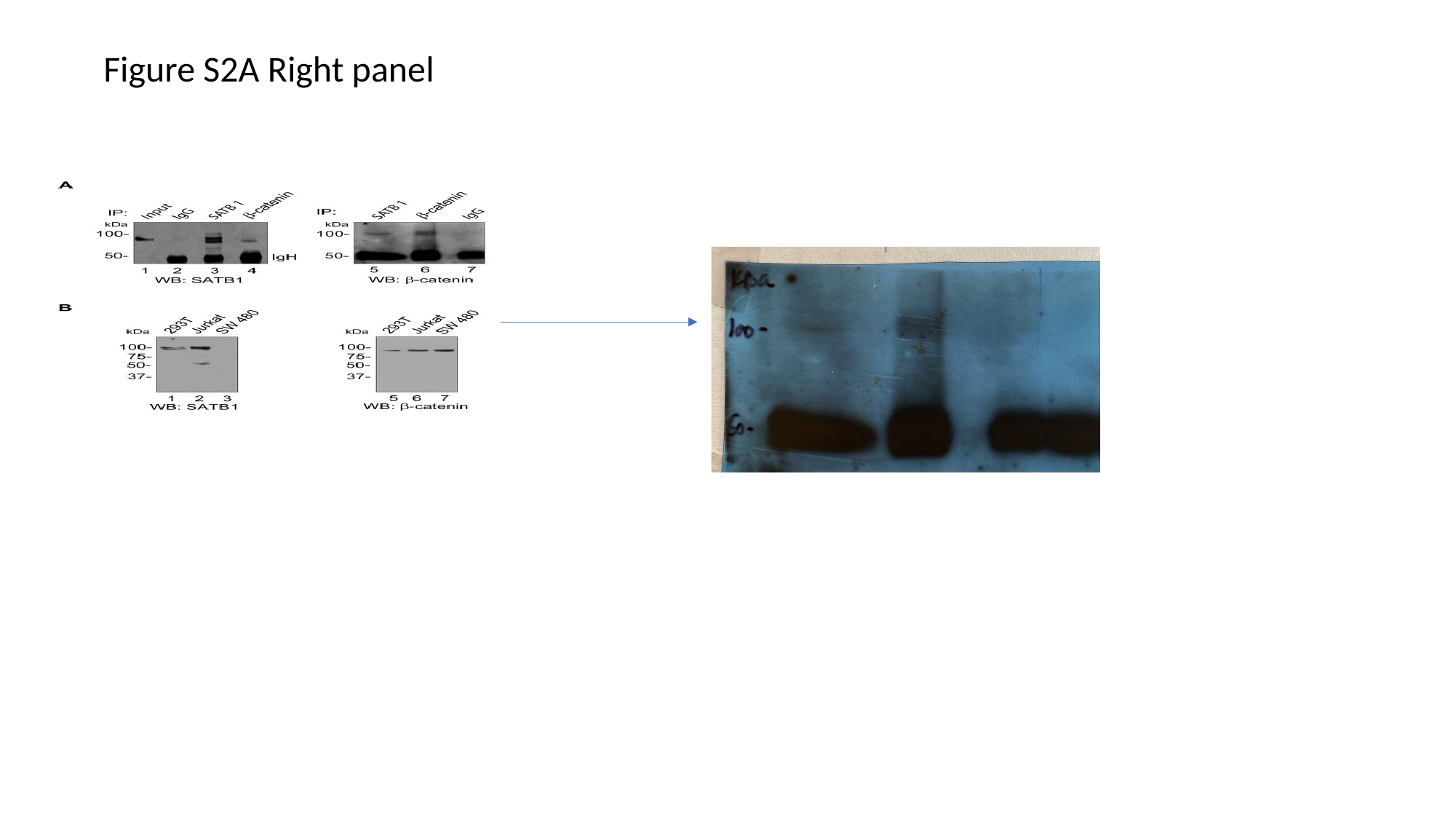

Figure S2A Right panel

## Slide 3
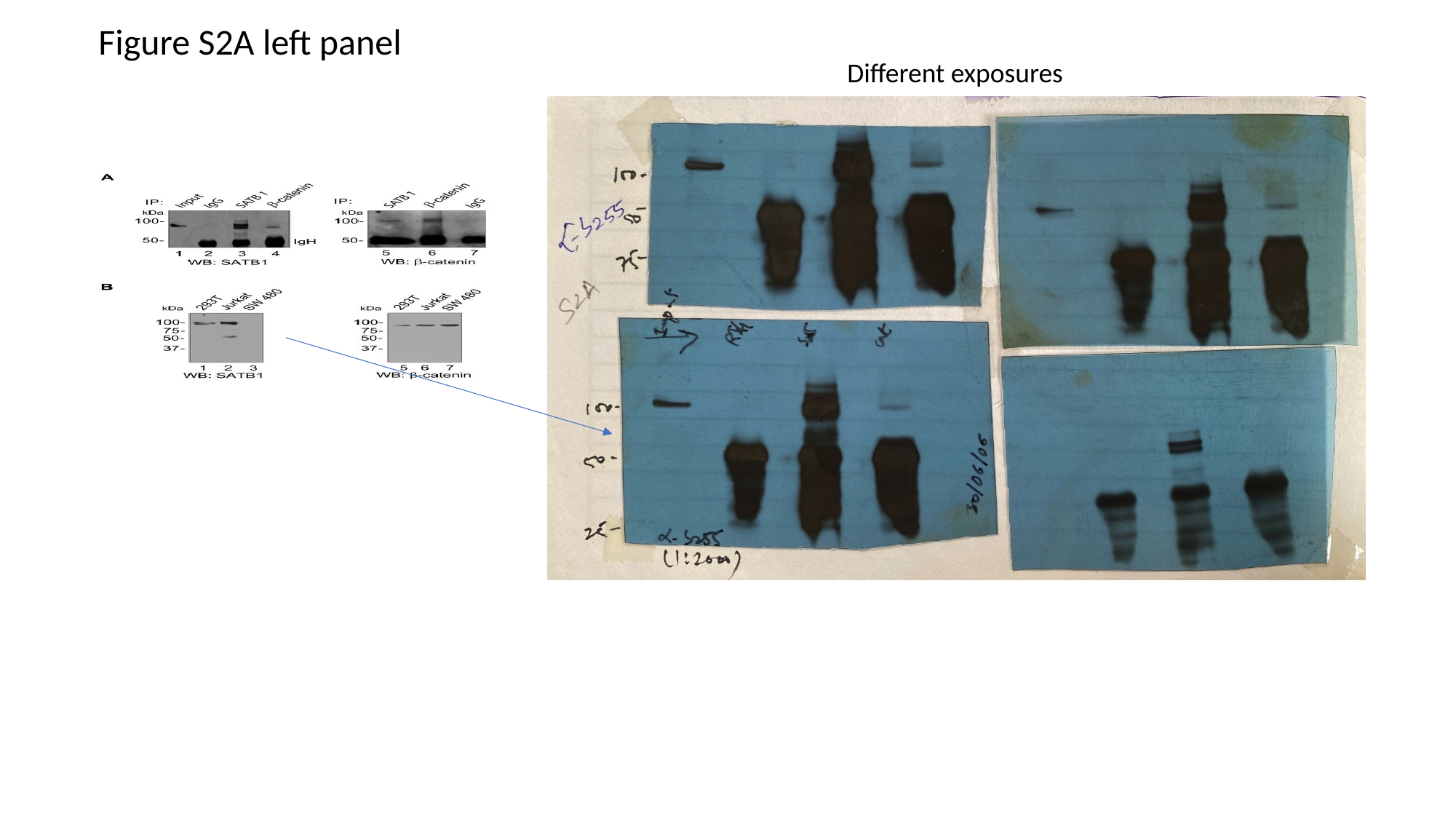

Figure S2A left panel
Different exposures

## Slide 4
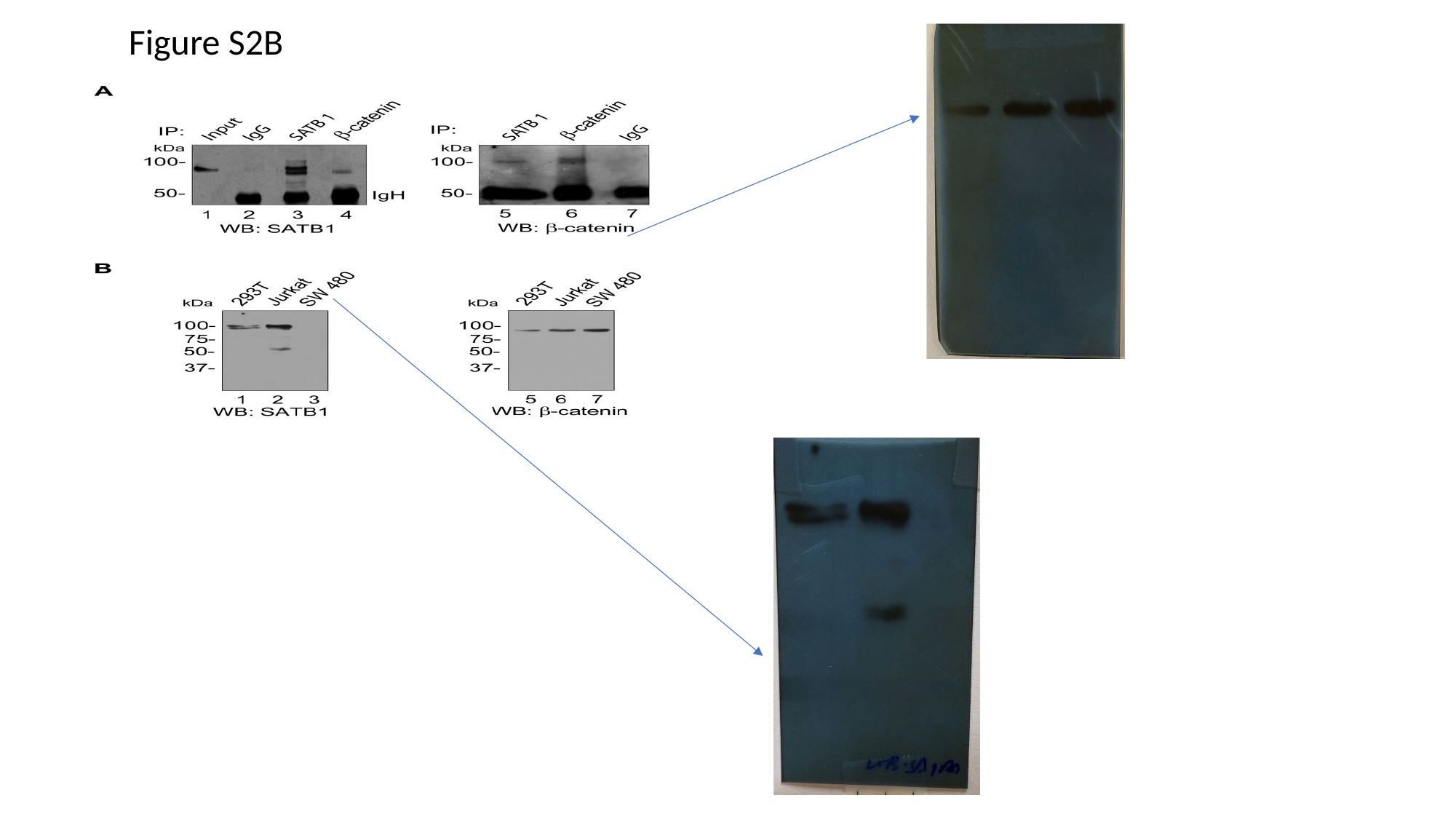

Figure S2B
