# Supplementary material for: Expression of Concern: Global Regulator SATB1 Recruits β-Catenin and Regulates TH2 Differentiation in Wnt-Dependent Manner
Source: PLoS Biol. 2022 Nov 23;20(11):e3001908. doi: 10.1371/journal.pbio.3001908 (PMC9683845; doi:10.1371/journal.pbio.3001908)
Supplement: S1 File — (ZIP) [file pbio.3001908.s001.zip › 6557773 Original Files/Fig S6 middle panel revised (1).pptx]

## Slide 1
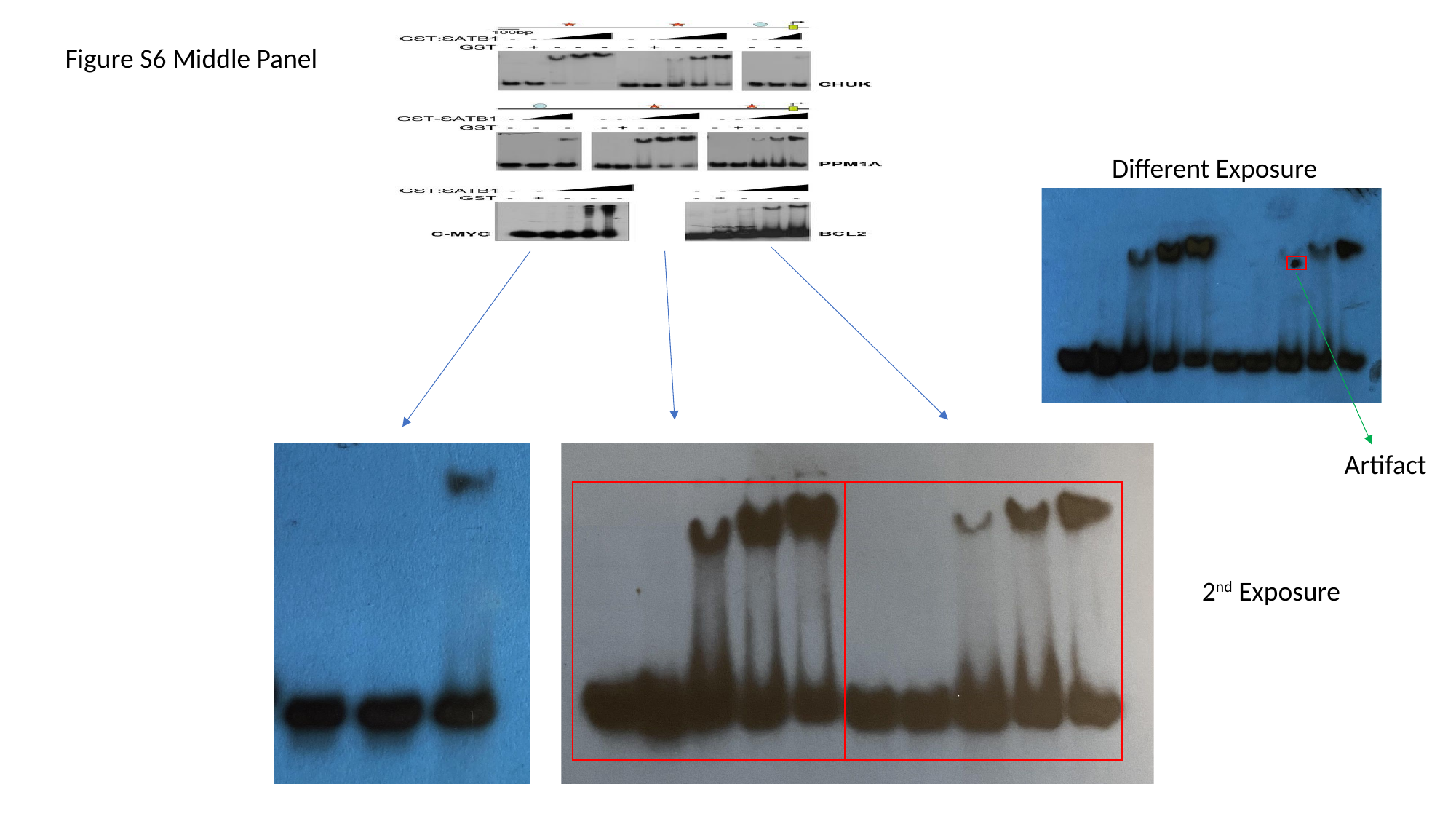

Figure S6 Middle Panel
 Different Exposure
Artifact
2nd Exposure

## Slide 2
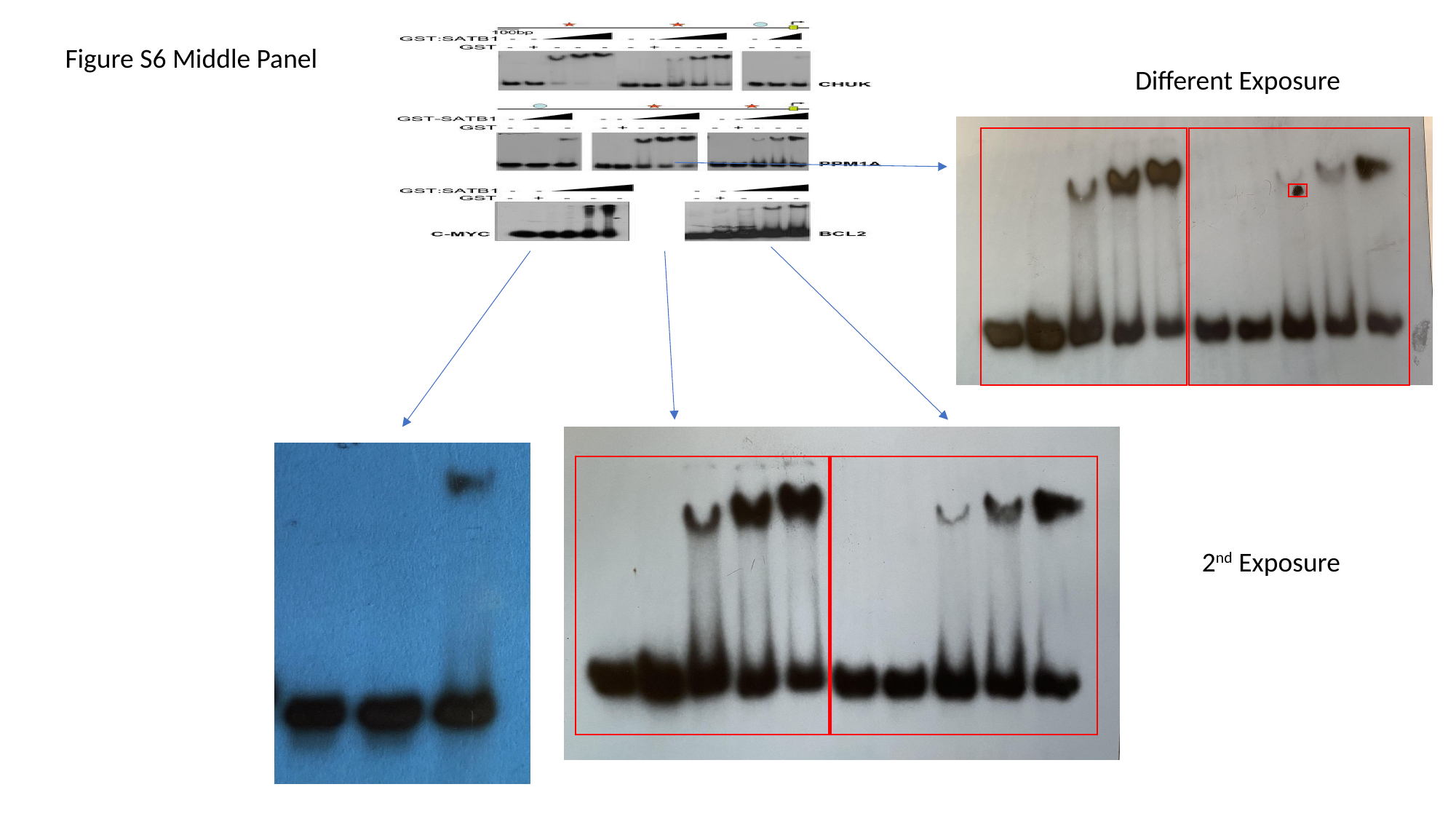

Figure S6 Middle Panel
 Different Exposure
2nd Exposure
