# Supplementary figures and images for: Expression of Concern: Global Regulator SATB1 Recruits β-Catenin and Regulates TH2 Differentiation in Wnt-Dependent Manner
Source: PLoS Biol. 2022 Nov 23;20(11):e3001908. doi: 10.1371/journal.pbio.3001908 (PMC9683845; doi:10.1371/journal.pbio.3001908)

## Slide 1
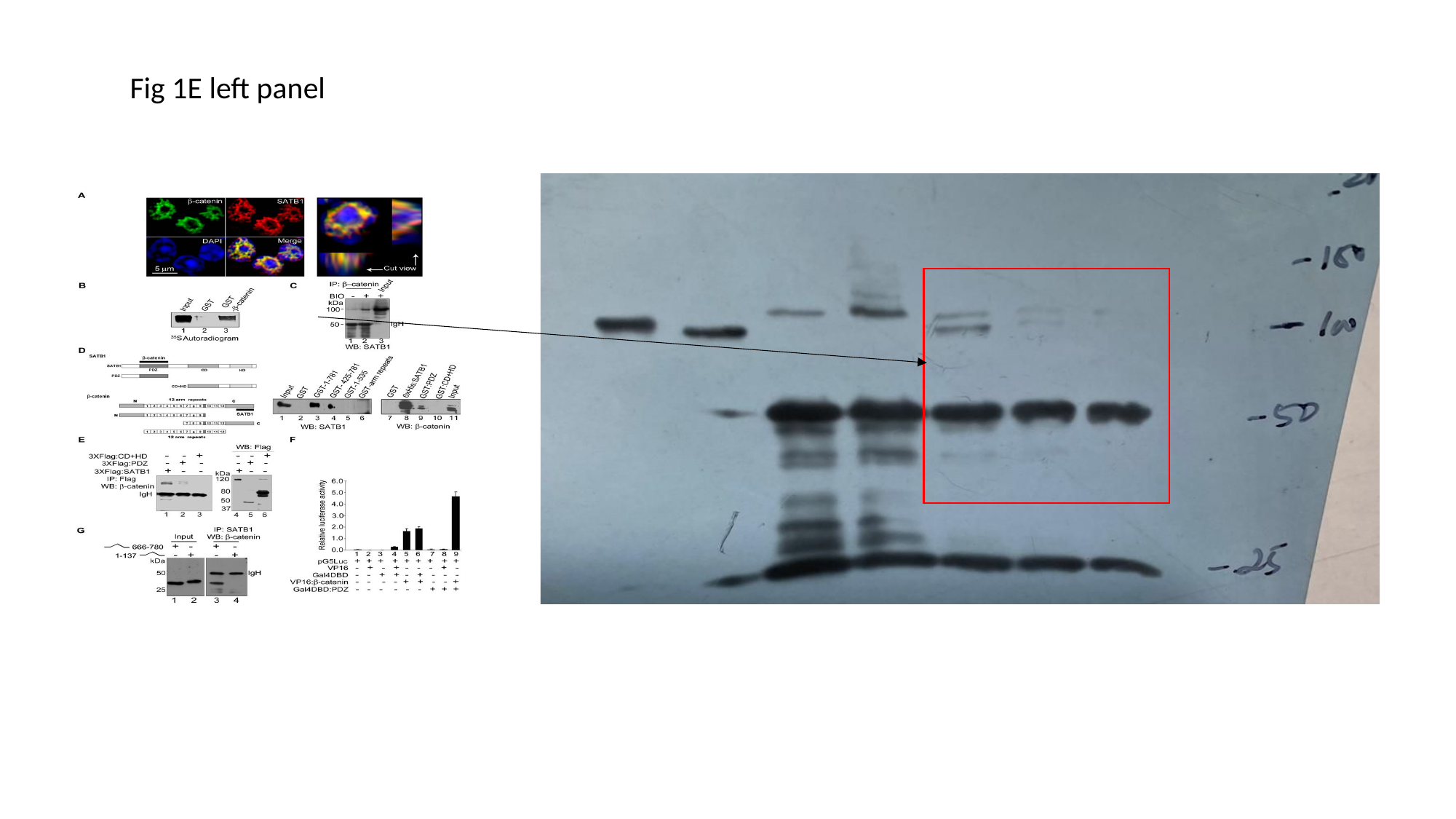

Fig 1E left panel

Supplement: S1 File — (ZIP) [file pbio.3001908.s001.zip › 6557773 Original Files/Fig 1E left panel (1) (1).pptx]

## Slide 1
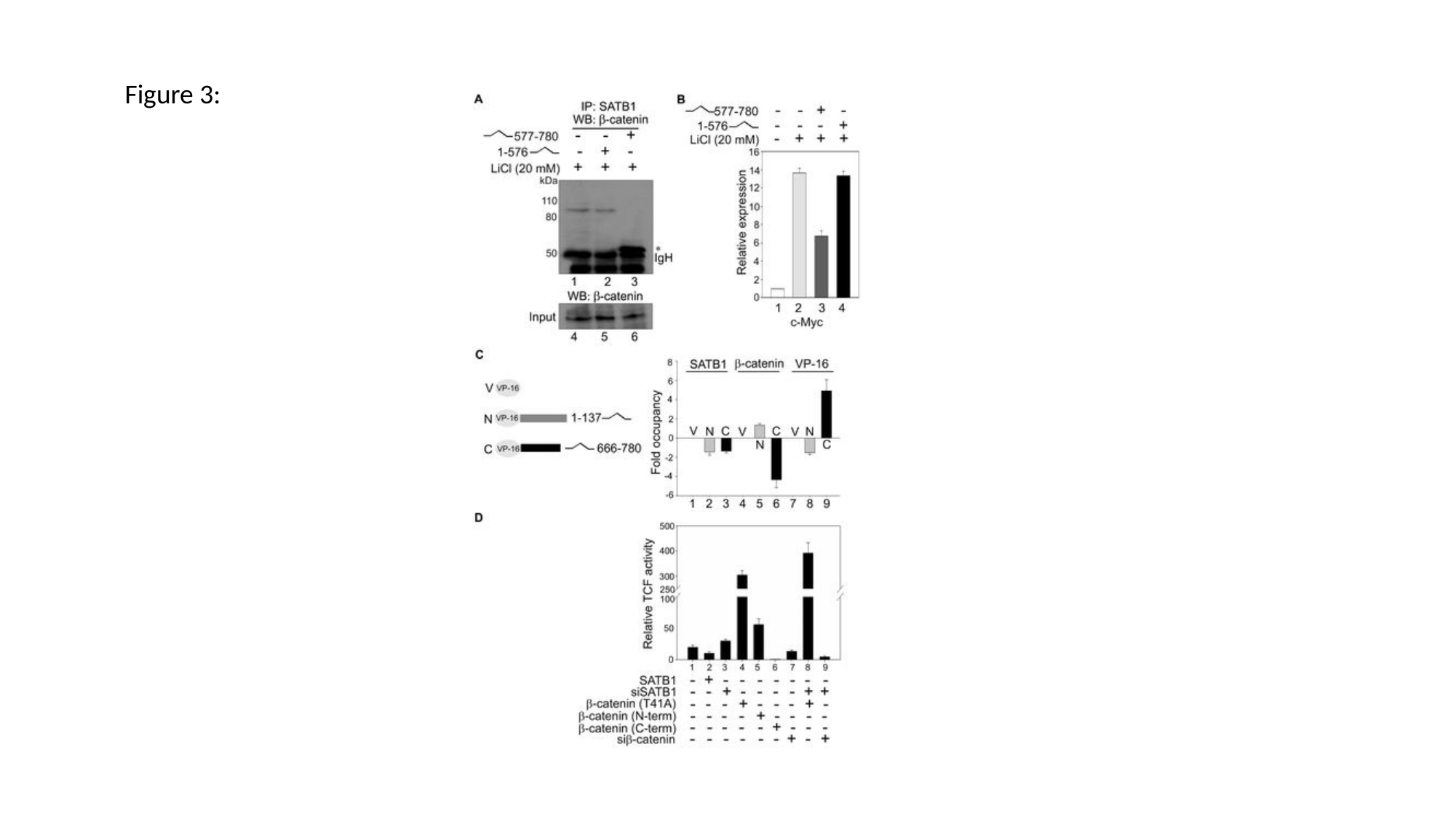

Figure 3:

## Slide 2
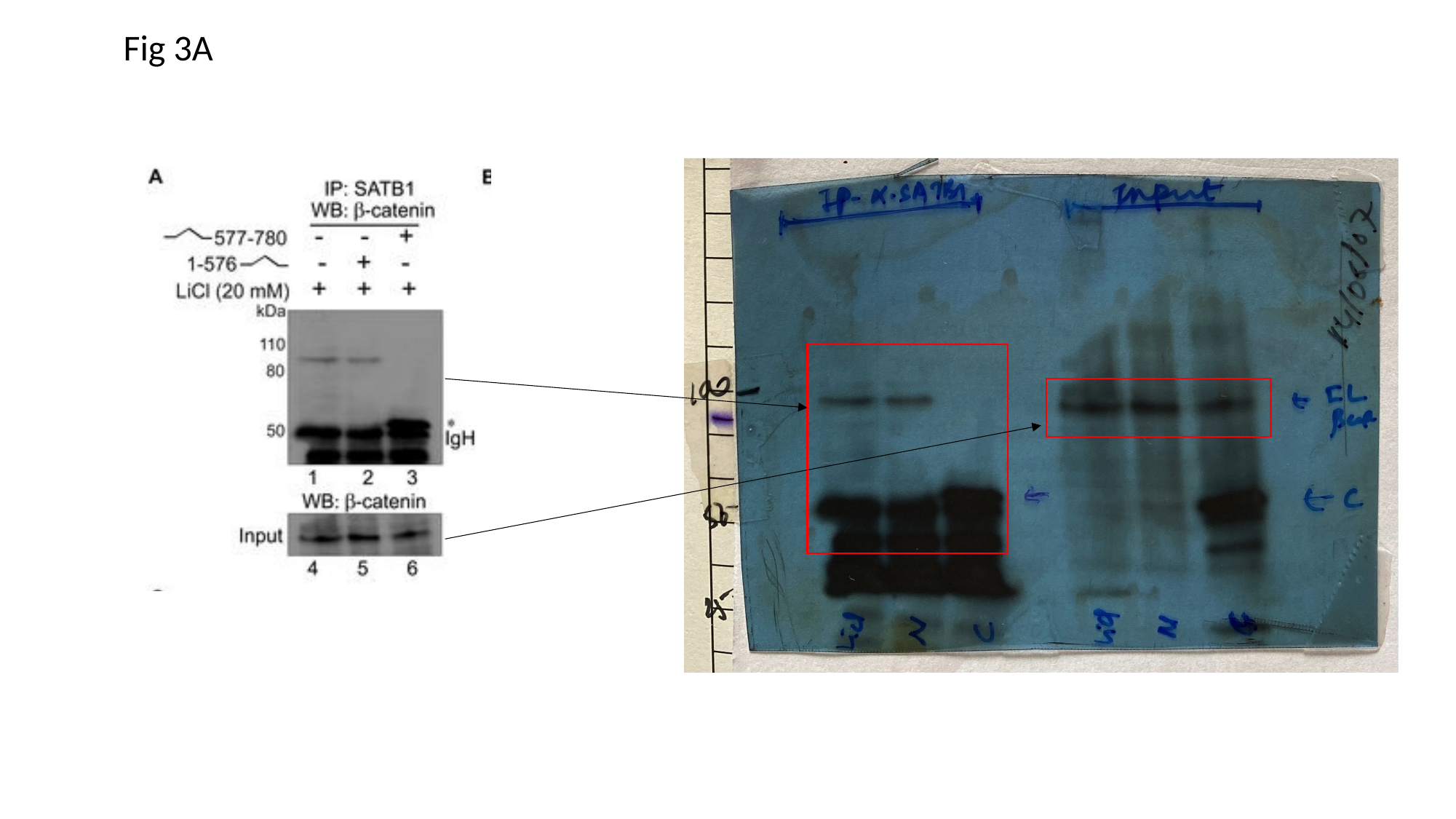

Fig 3A

Supplement: S1 File — (ZIP) [file pbio.3001908.s001.zip › 6557773 Original Files/Fig 3.pptx]

## Slide 1
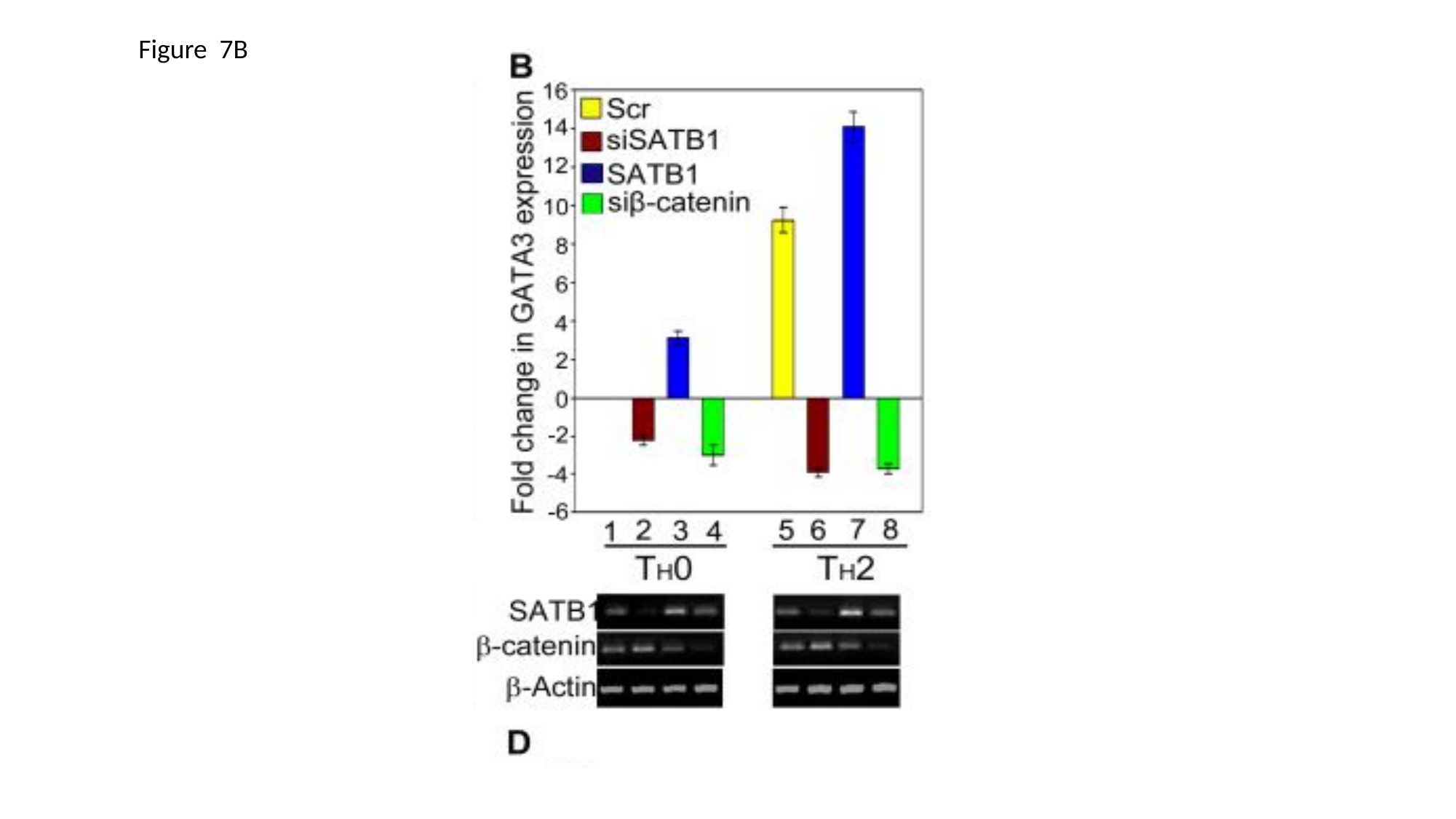

Figure 7B

## Slide 2
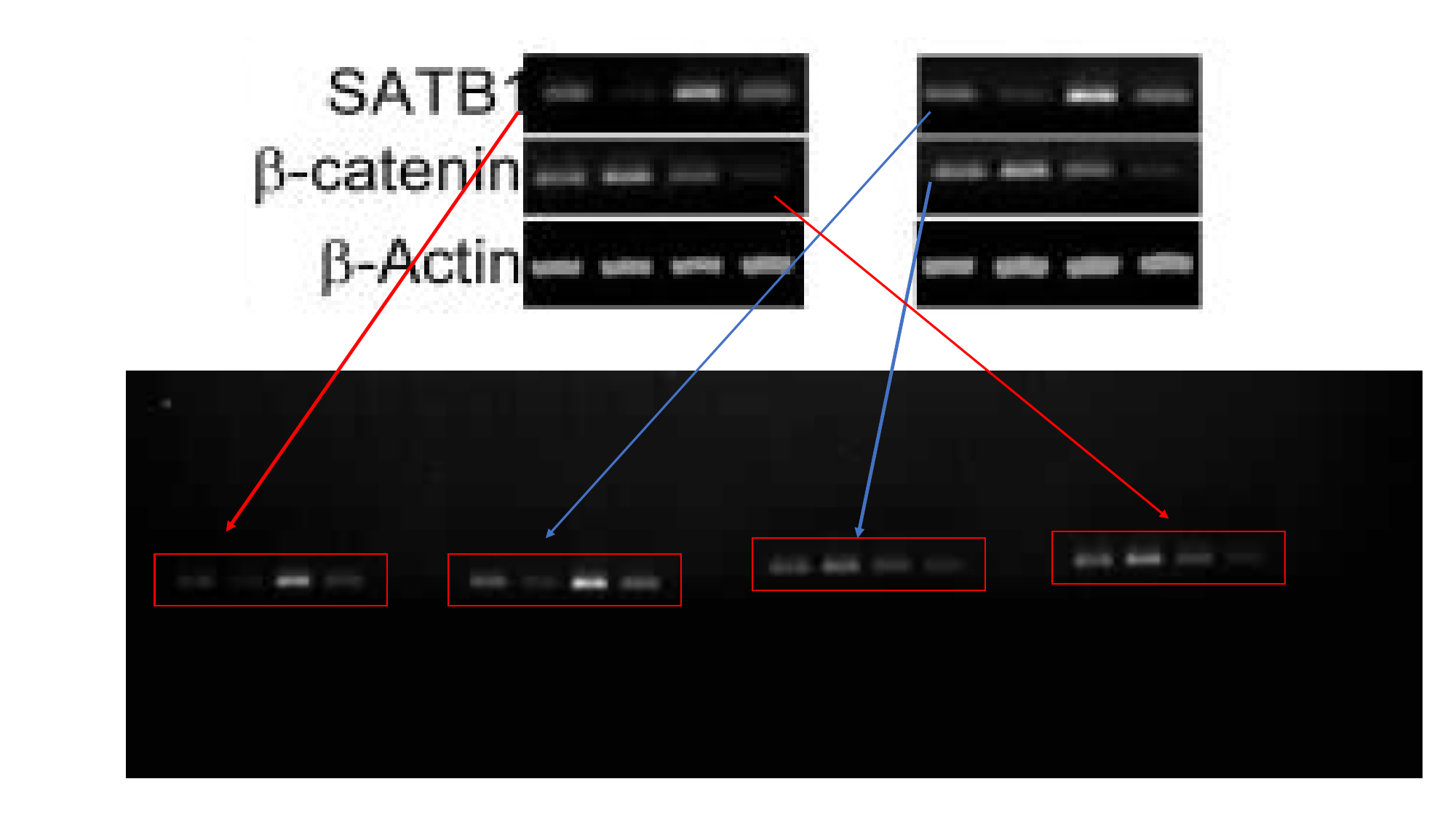

Supplement: S1 File — (ZIP) [file pbio.3001908.s001.zip › 6557773 Original Files/Fig 7b revised (2).pptx]

## Slide 1
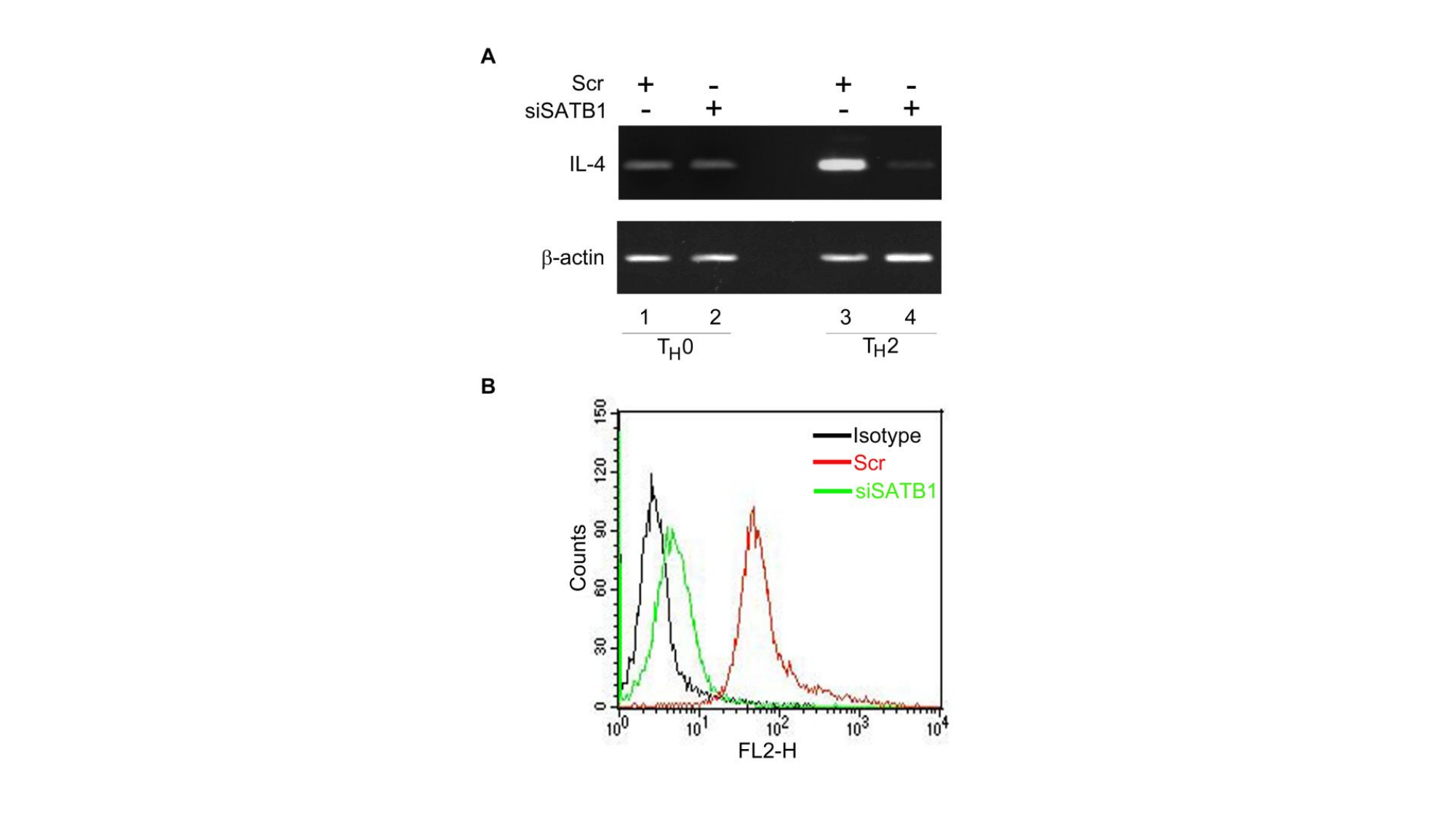

## Slide 2
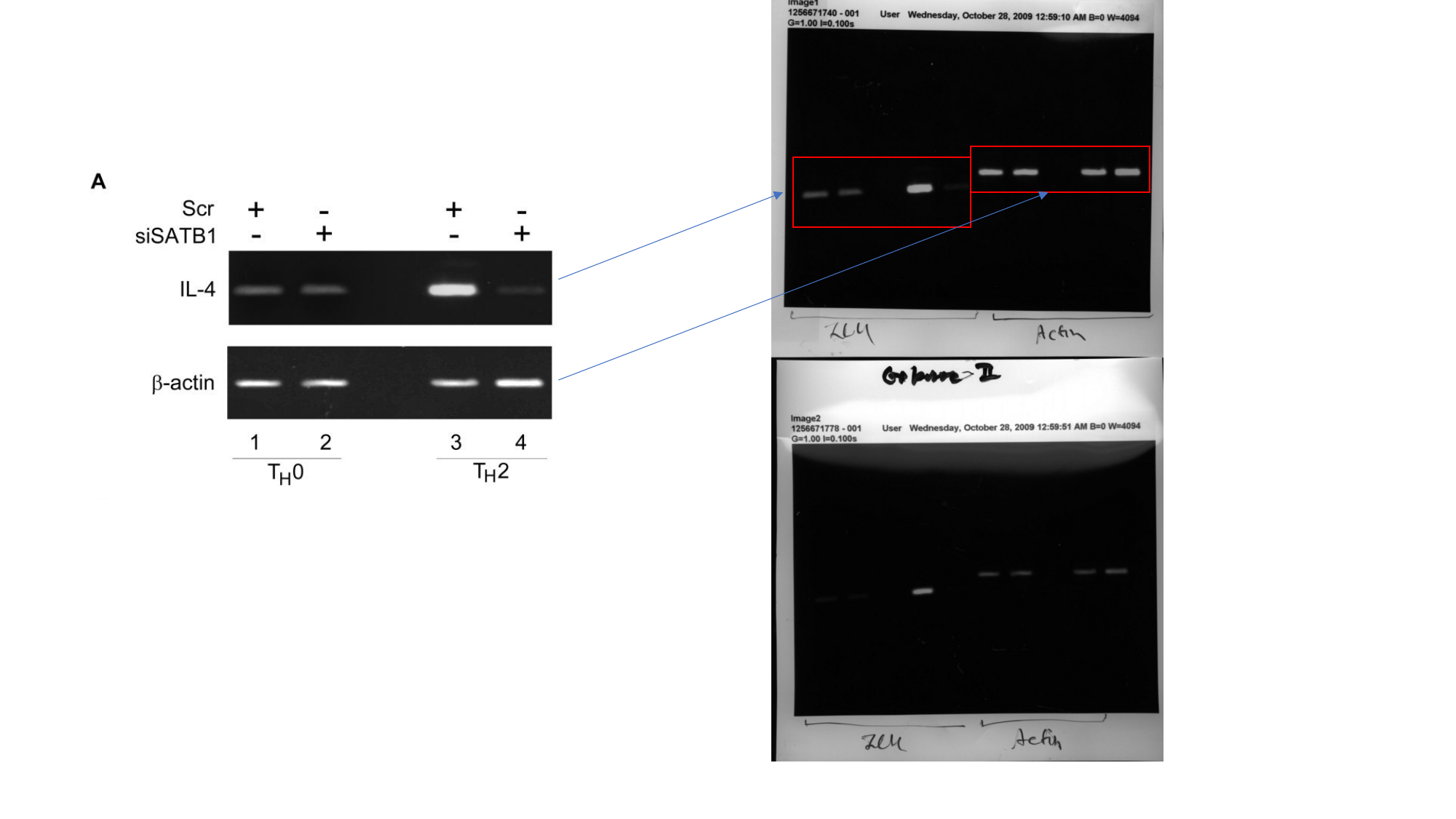

Supplement: S1 File — (ZIP) [file pbio.3001908.s001.zip › 6557773 Original Files/Fig S11a.pptx]

## Slide 1
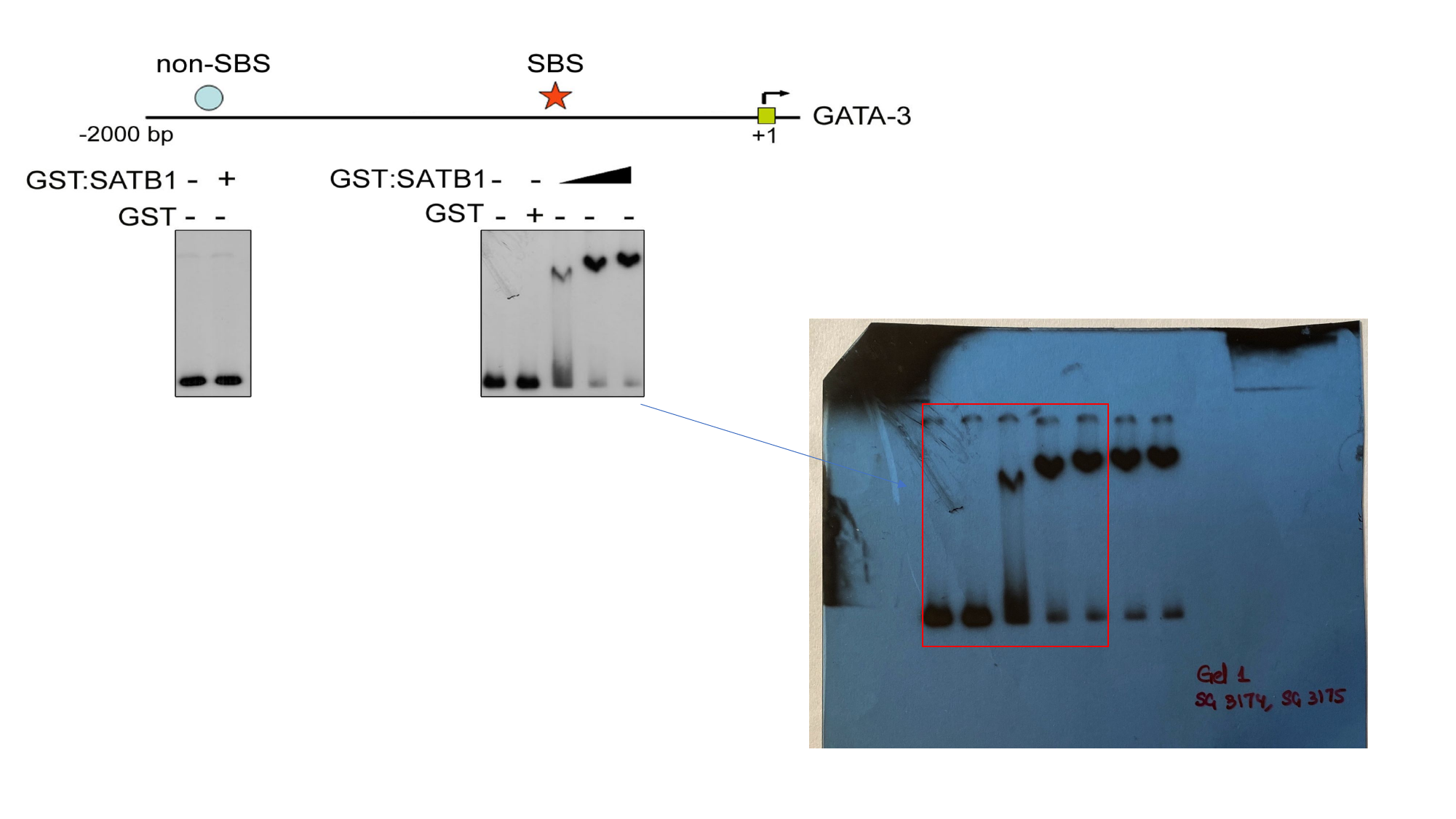

Supplement: S1 File — (ZIP) [file pbio.3001908.s001.zip › 6557773 Original Files/Fig S12.pptx]

## Slide 1
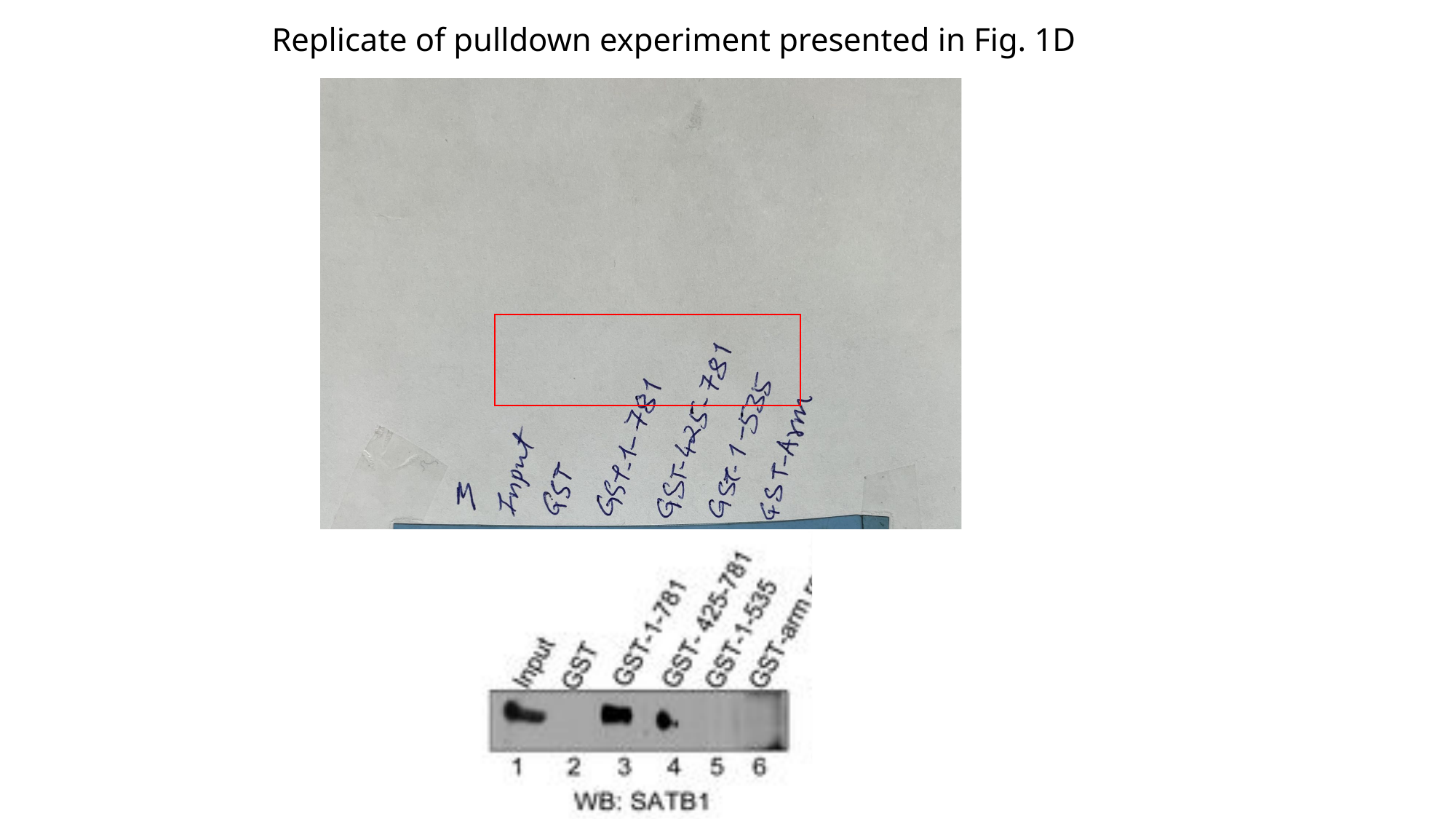

# Replicate of pulldown experiment presented in Fig. 1D

Supplement: S1 File — (ZIP) [file pbio.3001908.s001.zip › 6557773 Original Files/Pulldown experiment Fig. 1D replicate.pptx]

## Slide 1
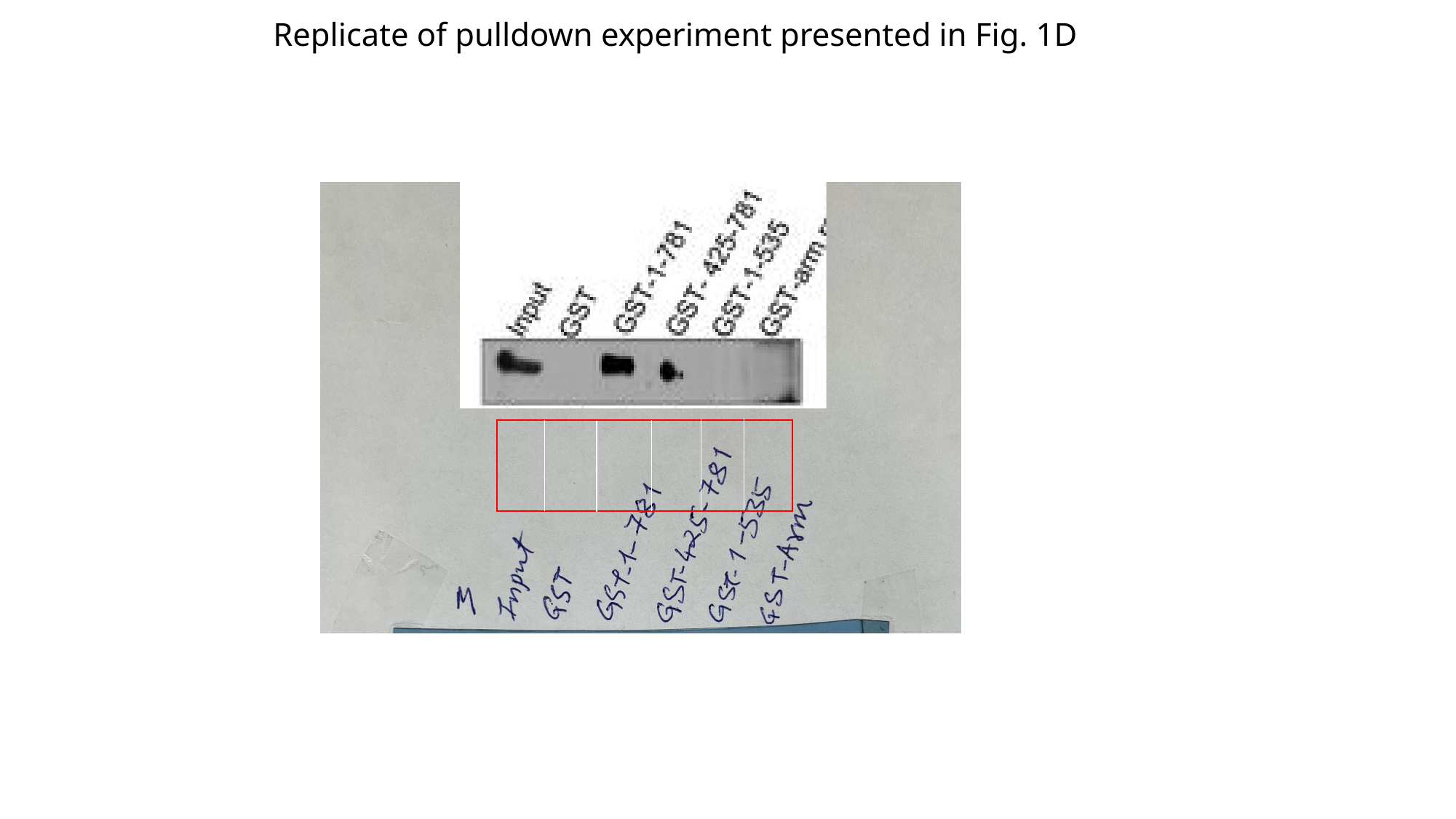

# Replicate of pulldown experiment presented in Fig. 1D

Supplement: S2 File — (ZIP) [file pbio.3001908.s002.zip › 6557773 Replicate Files/Fig. 1D Pulldown experiment replicate_lanes overlapped with published image (2).pptx]
