# Supplementary material for: Expression of Concern: Global Regulator SATB1 Recruits β-Catenin and Regulates TH2 Differentiation in Wnt-Dependent Manner
Source: PLoS Biol. 2022 Nov 23;20(11):e3001908. doi: 10.1371/journal.pbio.3001908 (PMC9683845; doi:10.1371/journal.pbio.3001908)
Supplement: S2 File — (ZIP) [file pbio.3001908.s002.zip › 6557773 Replicate Files/Coomassie blue stained protein gels_New.pptx]

## Slide 1
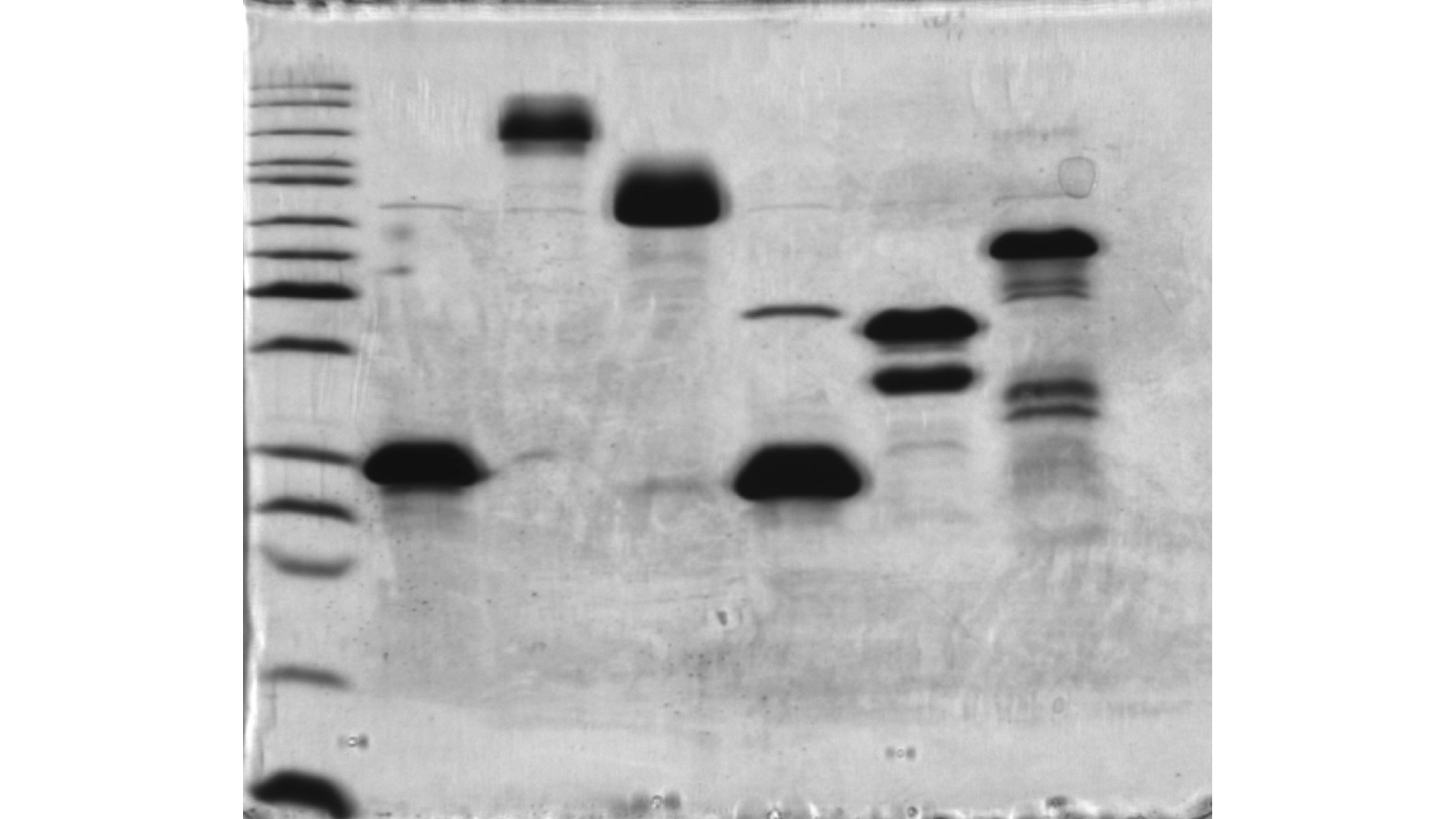

## Slide 2
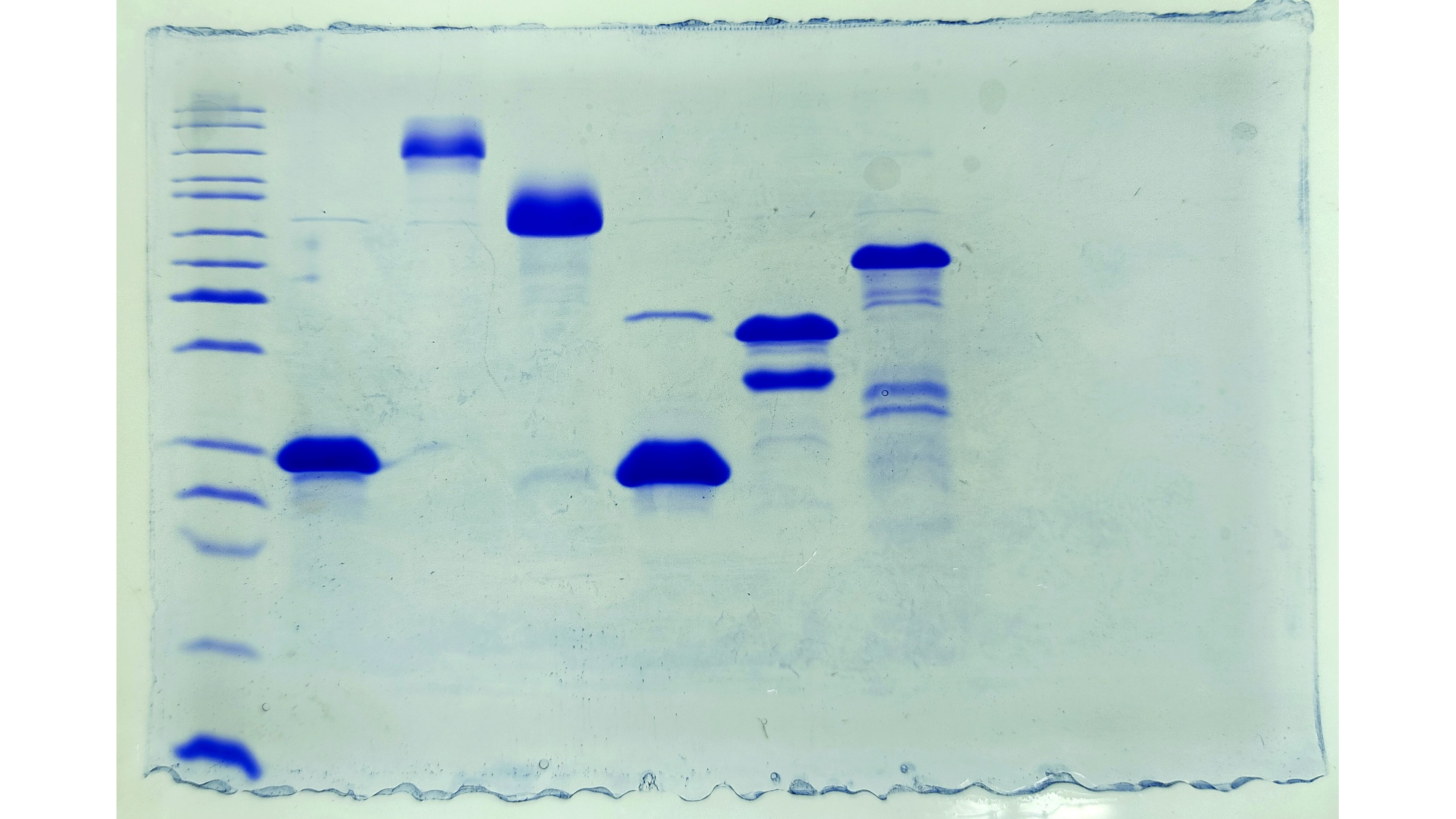

## Slide 3
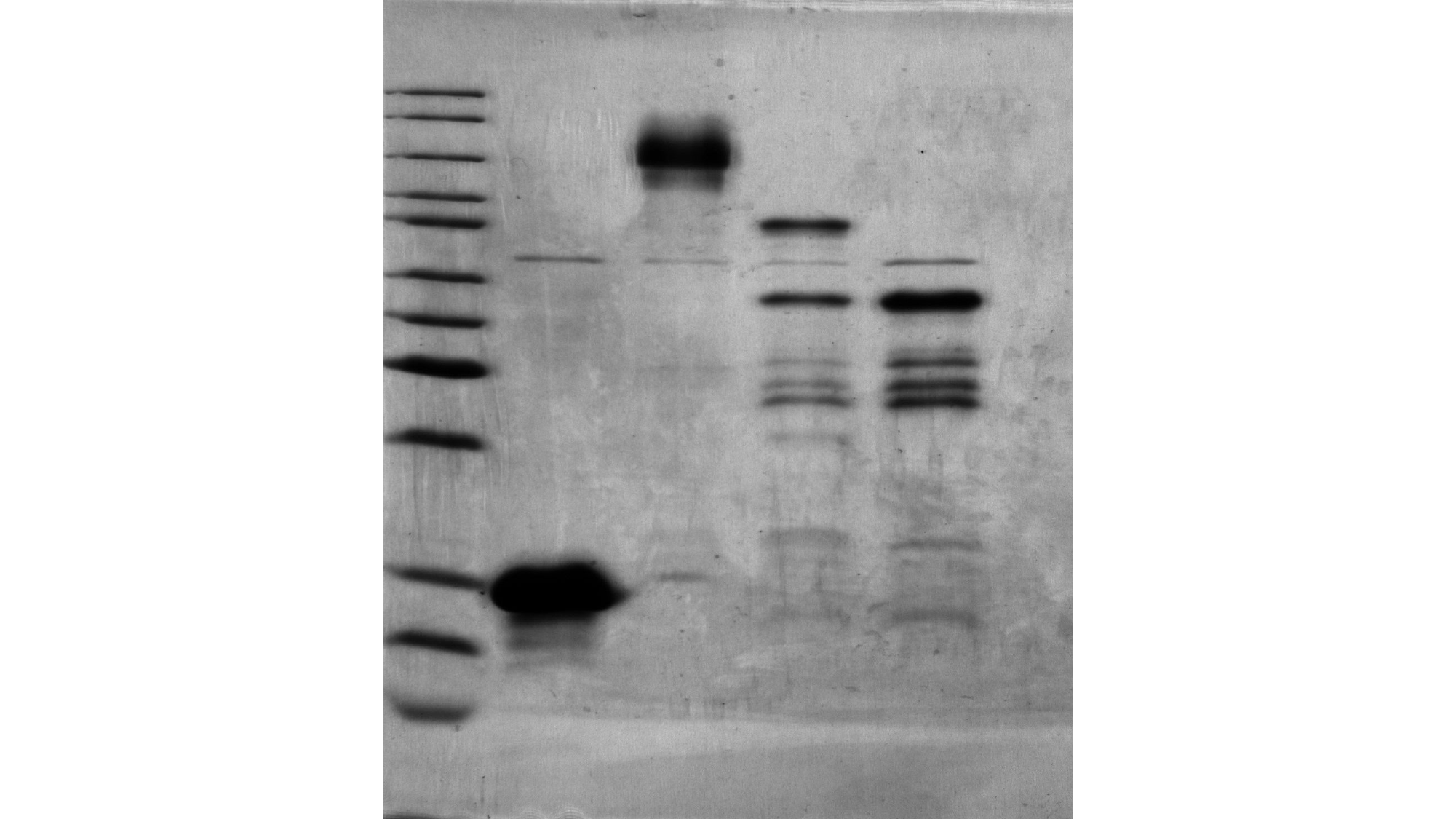

## Slide 4
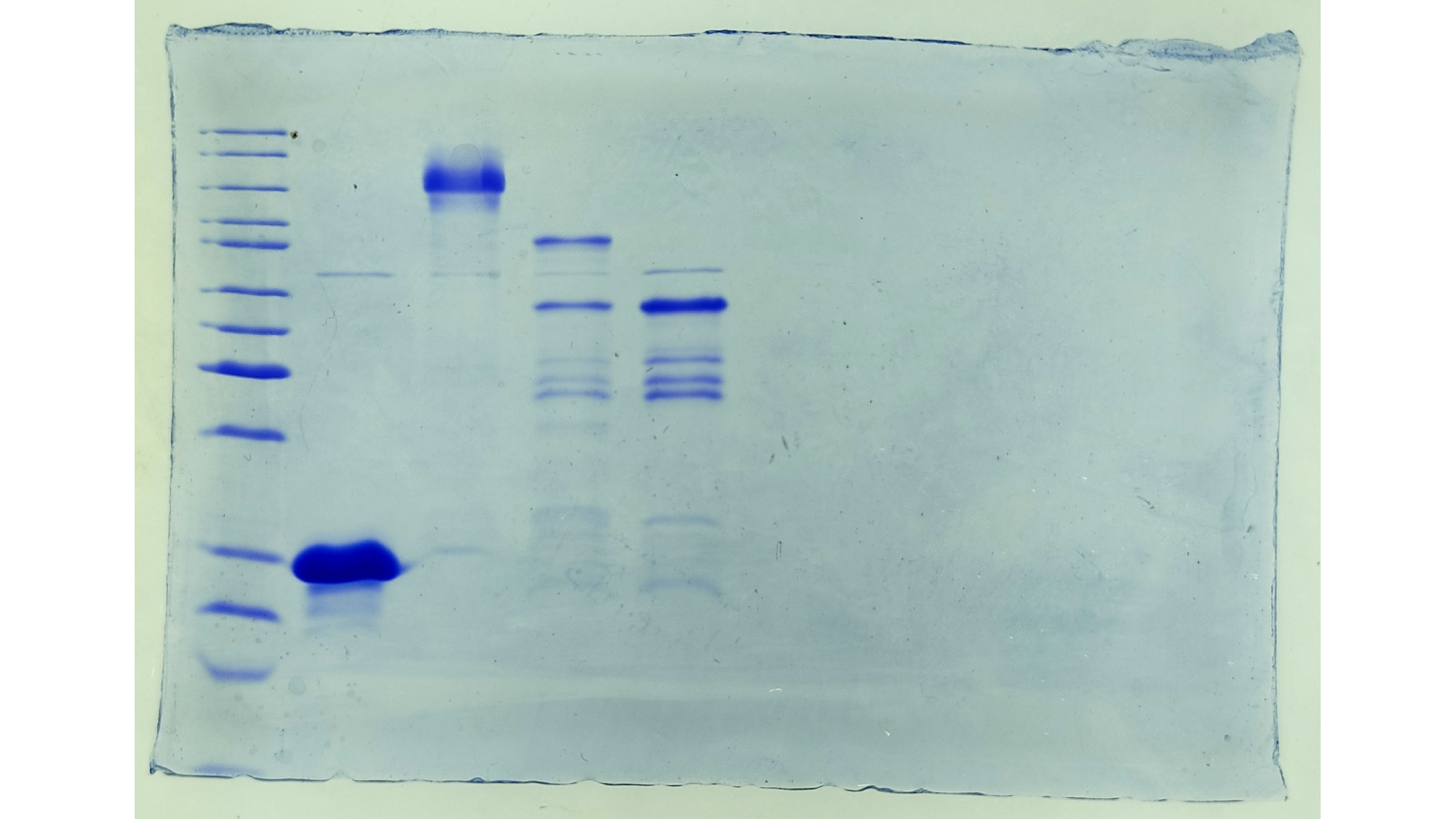

## Slide 5
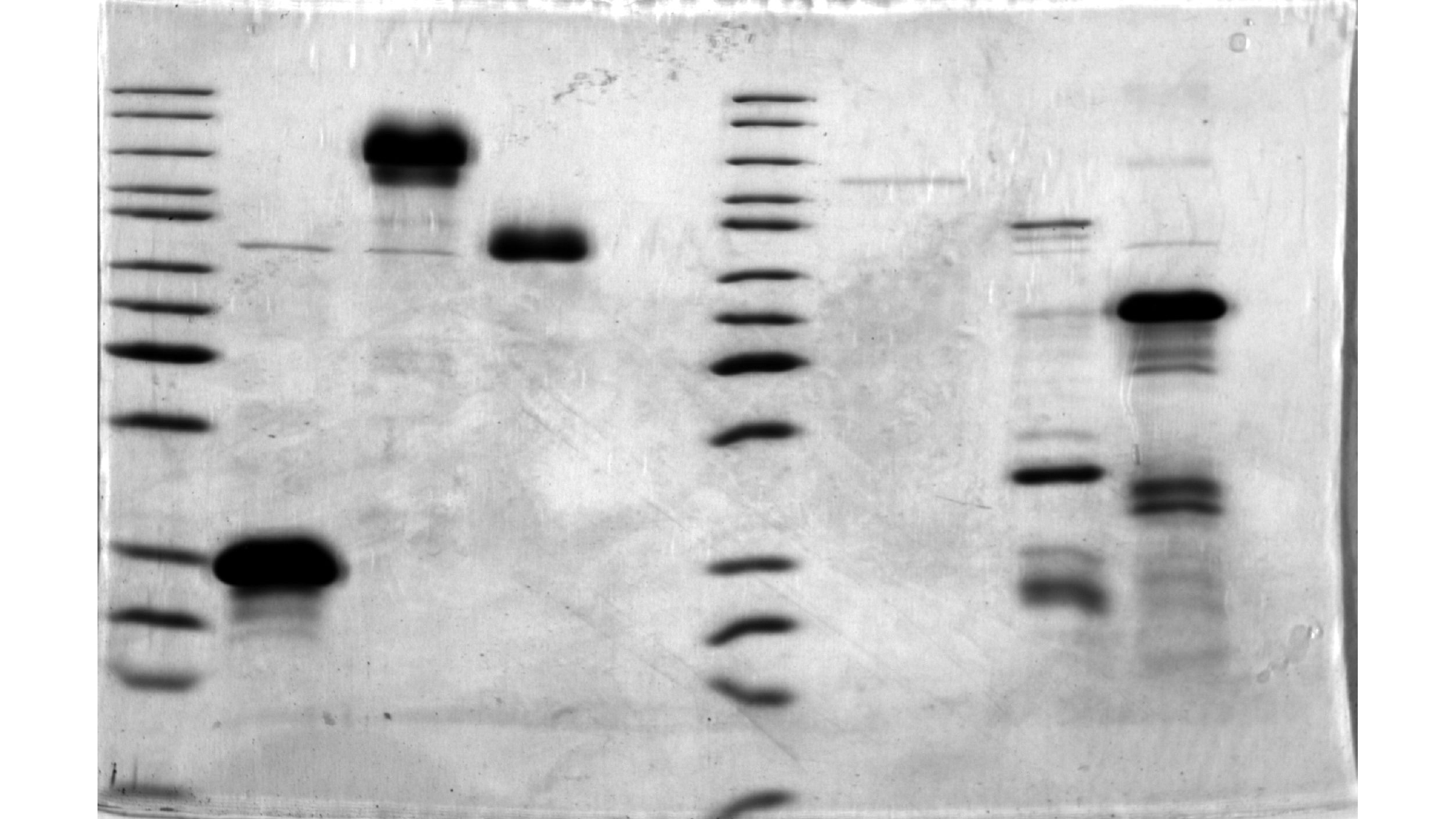

## Slide 6
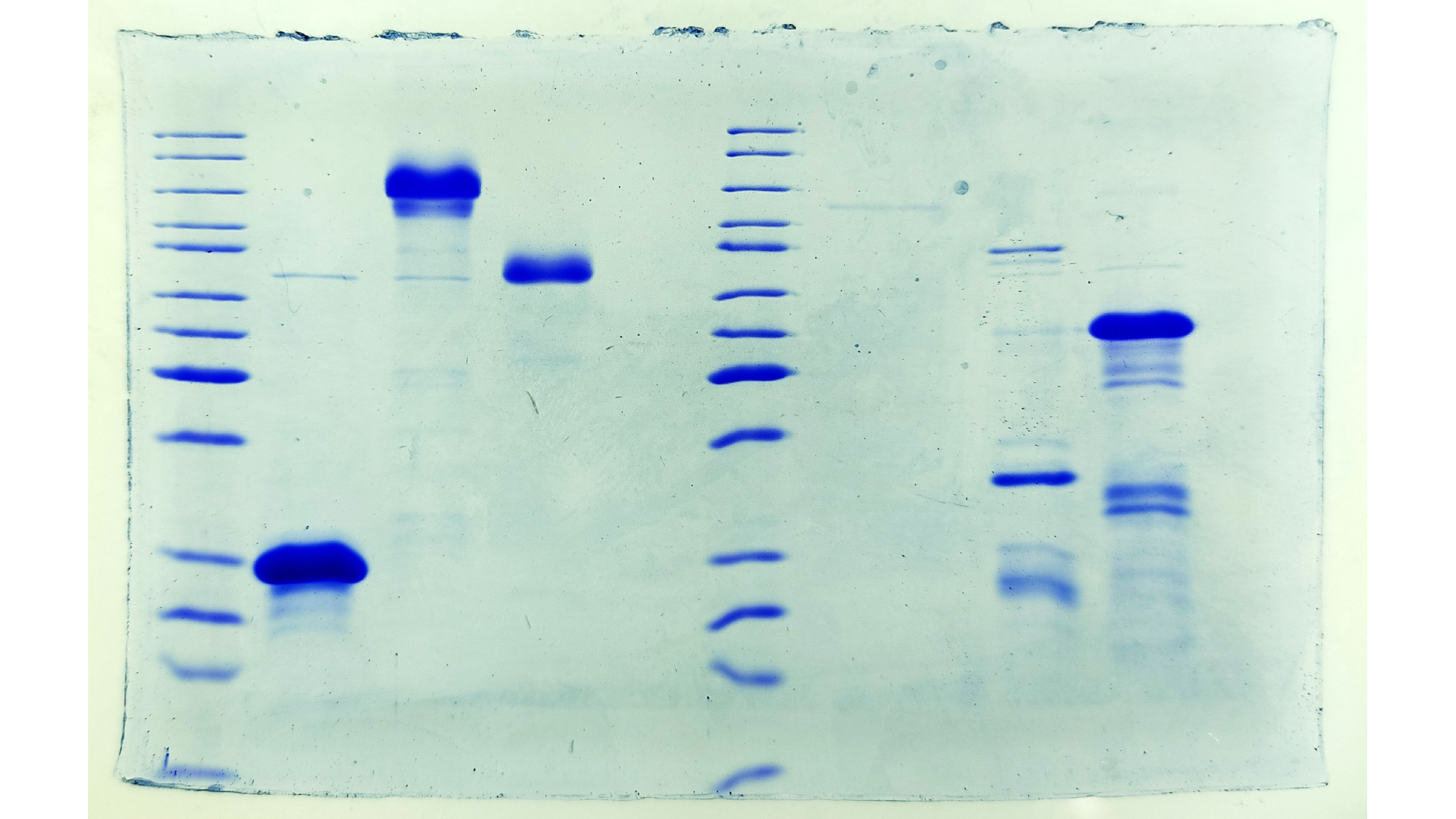

## Slide 7
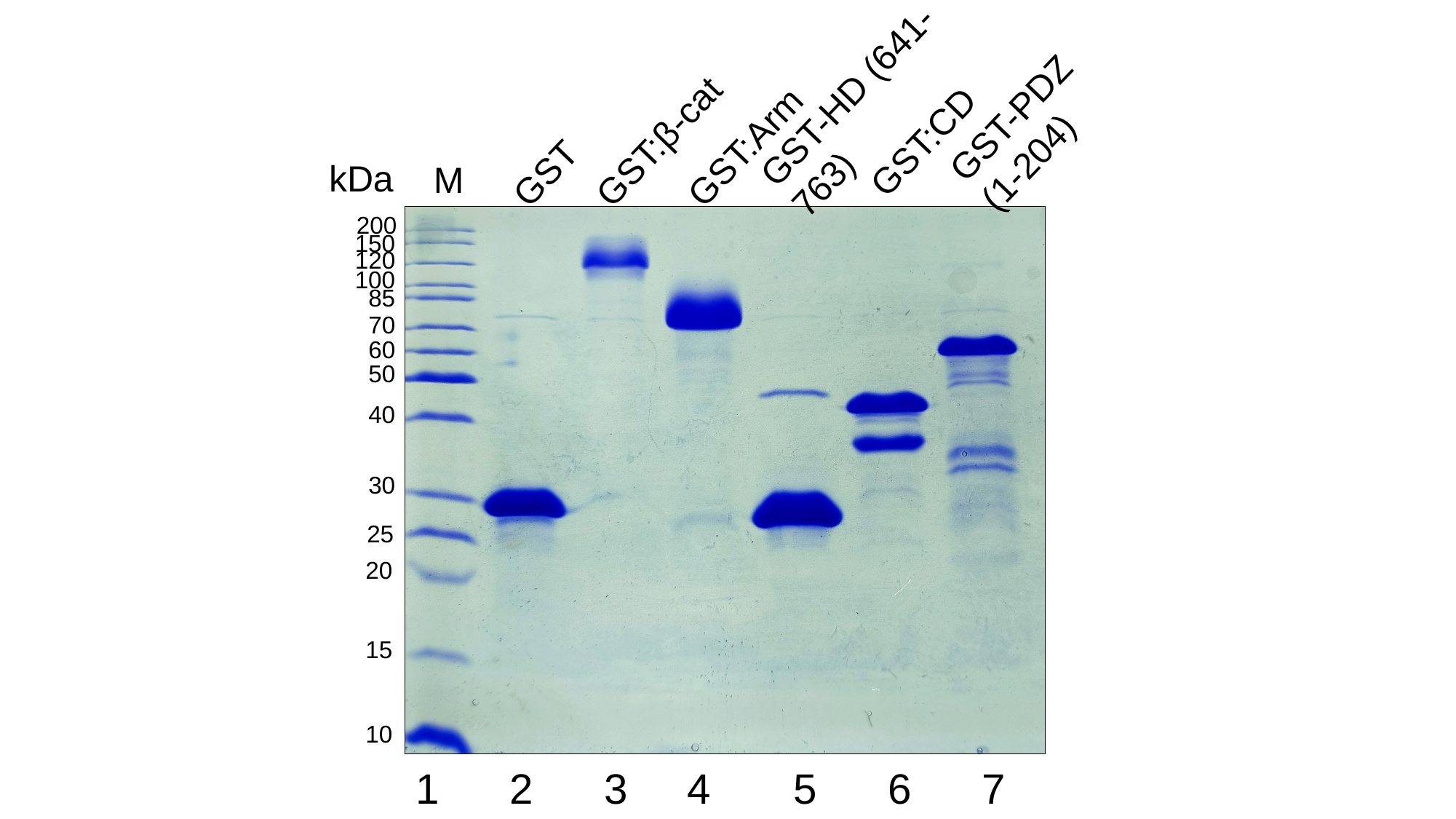

GST-HD (641-763)
GST-PDZ (1-204)
GST:CD
GST:β-cat
GST:Arm
GST
kDa
M
200
150
120
100
85
70
60
50
40
30
25
20
15
10
1 2 3 4 5 6 7

## Slide 8
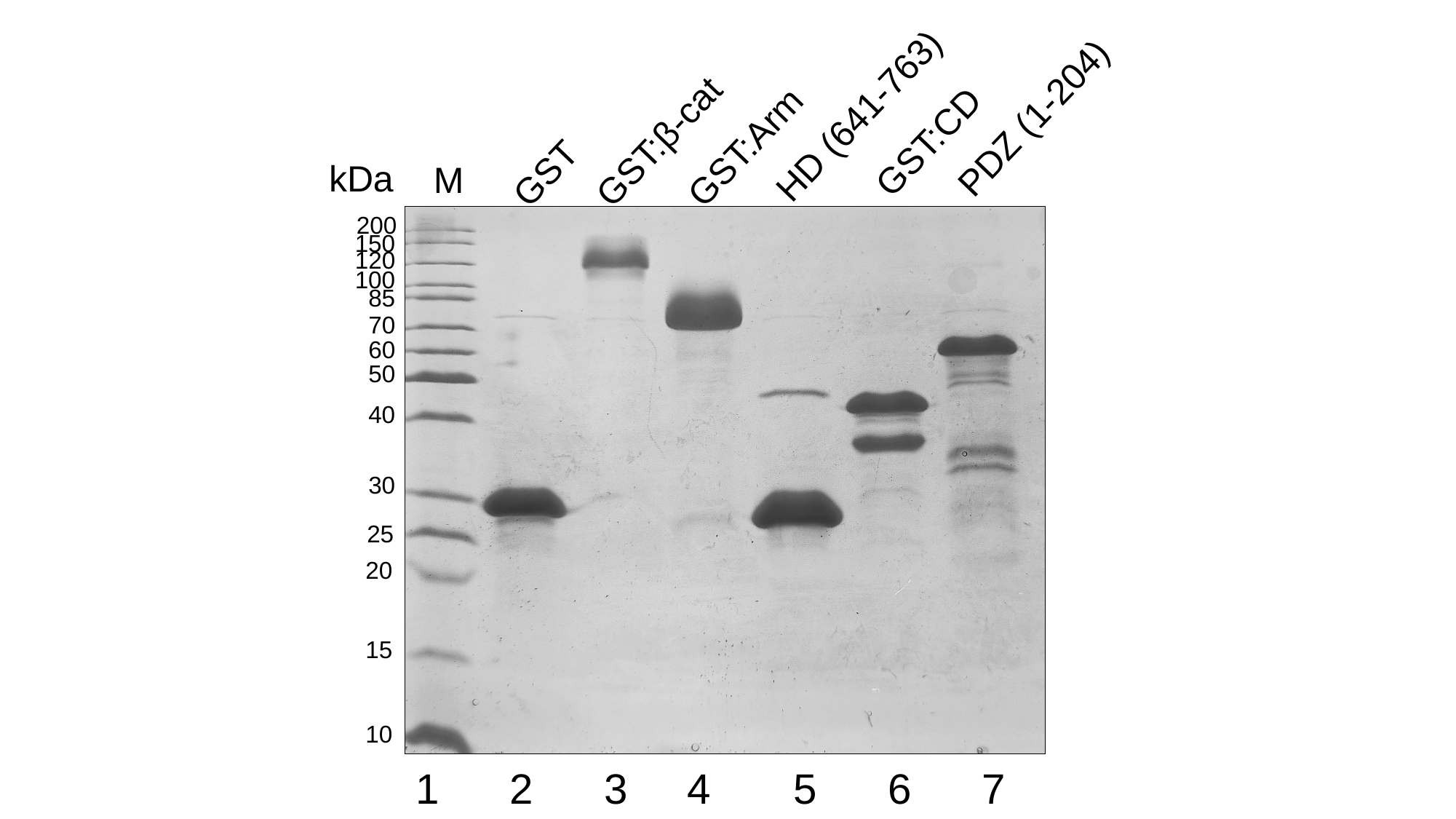

HD (641-763)
PDZ (1-204)
GST:CD
GST:β-cat
GST:Arm
GST
kDa
M
200
150
120
100
85
70
60
50
40
30
25
20
15
10
1 2 3 4 5 6 7

## Slide 9
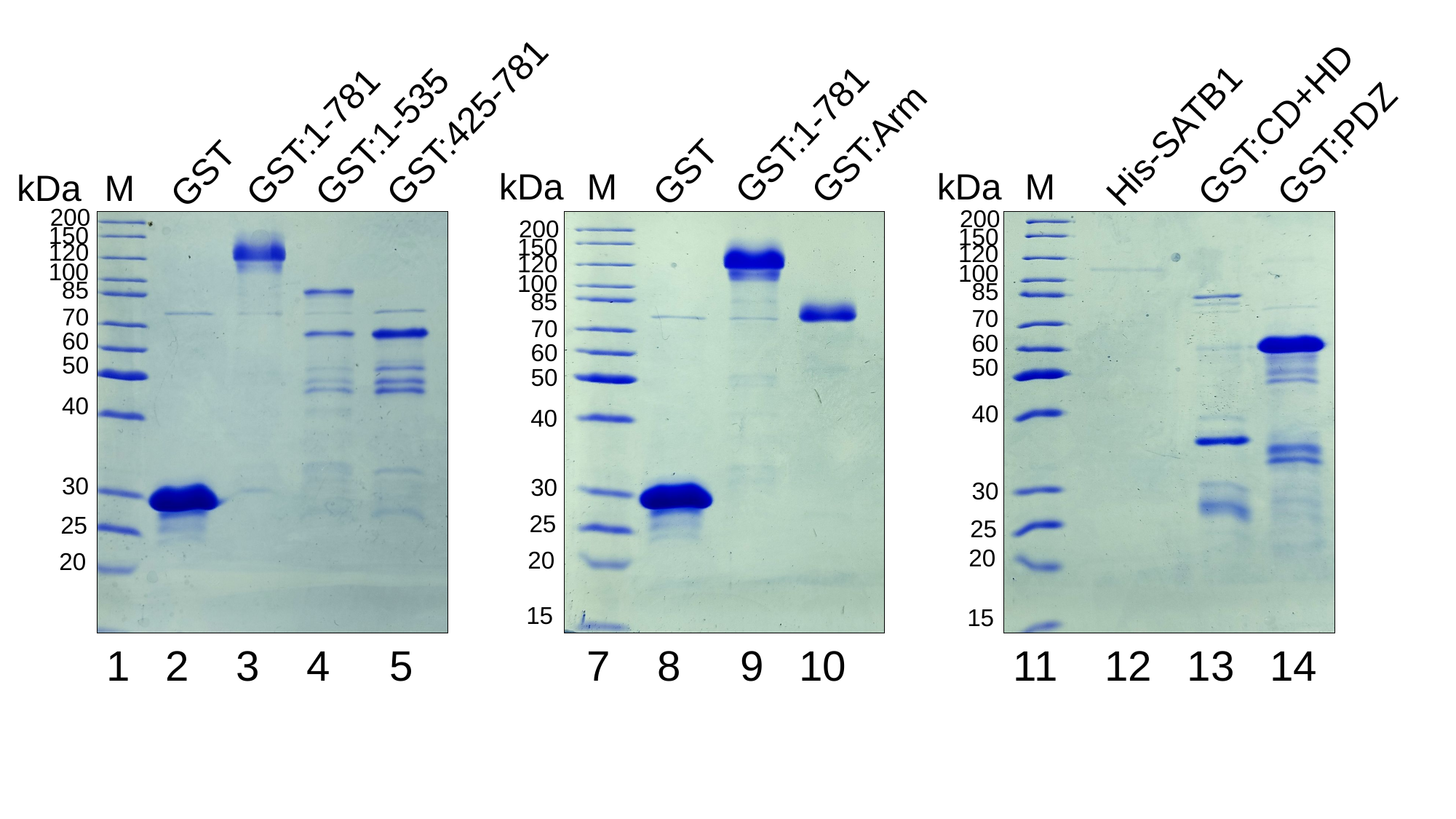

GST:425-781
GST:CD+HD
His-SATB1
GST:1-781
GST:Arm
GST:PDZ
GST:1-535
GST:1-781
GST
GST
kDa
kDa
M
M
kDa
M
200
150
120
100
85
70
60
50
40
30
25
20
200
150
120
100
85
70
60
50
40
30
25
20
15
200
150
120
100
85
70
60
50
40
30
25
20
15
1 2 3 4 5
 7 8 9 10
11 12 13 14

## Slide 10
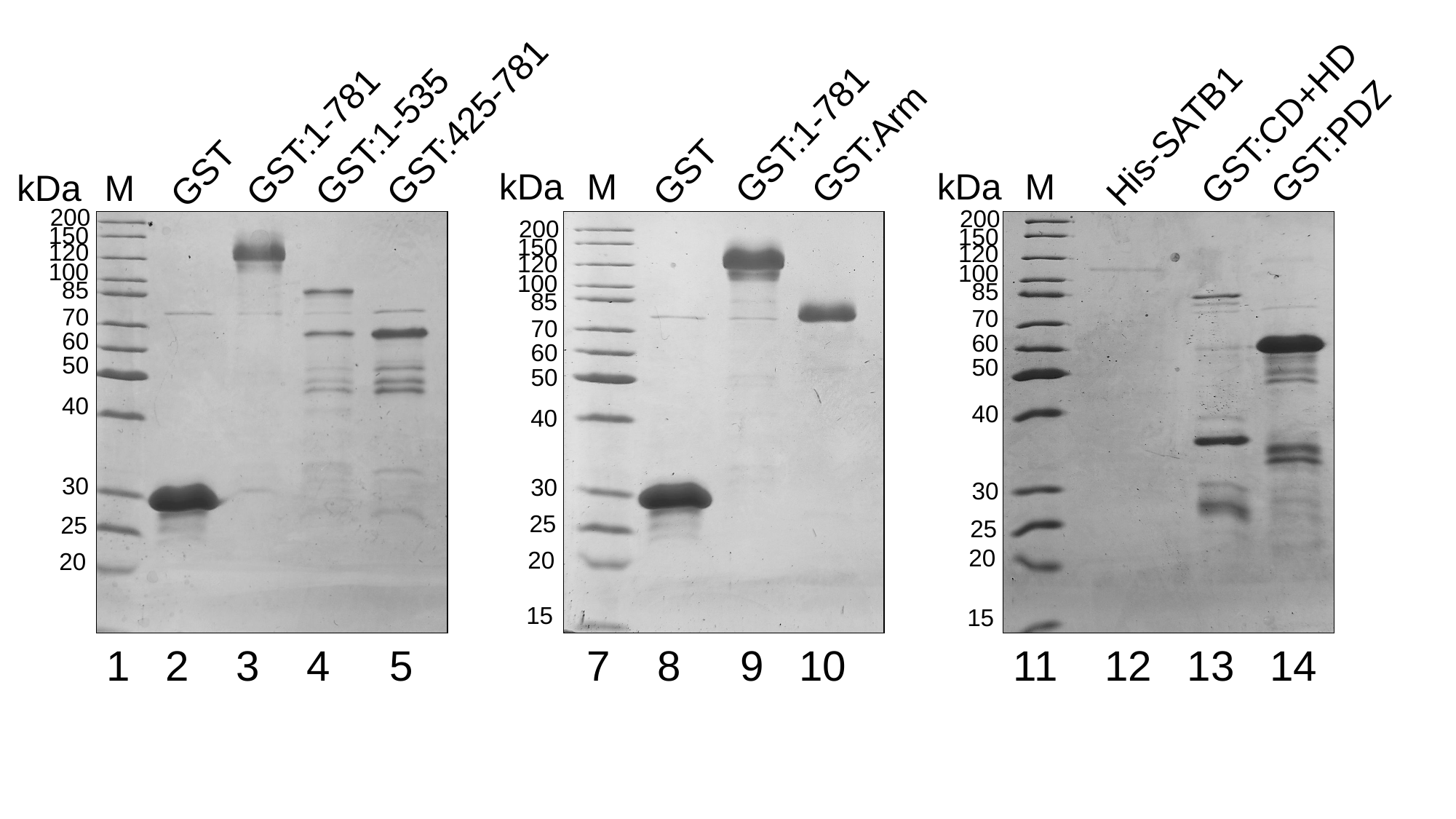

GST:425-781
GST:CD+HD
His-SATB1
GST:1-781
GST:PDZ
GST:Arm
GST:1-535
GST:1-781
GST
GST
kDa
kDa
M
M
kDa
M
200
150
120
100
85
70
60
50
40
30
25
20
200
150
120
100
85
70
60
50
40
30
25
20
15
200
150
120
100
85
70
60
50
40
30
25
20
15
1 2 3 4 5
 7 8 9 10
11 12 13 14
