# Supplementary material for: Expression of Concern: Global Regulator SATB1 Recruits β-Catenin and Regulates TH2 Differentiation in Wnt-Dependent Manner
Source: PLoS Biol. 2022 Nov 23;20(11):e3001908. doi: 10.1371/journal.pbio.3001908 (PMC9683845; doi:10.1371/journal.pbio.3001908)

Interaction of SATB1 with Full-length beta-catenin (1-781)

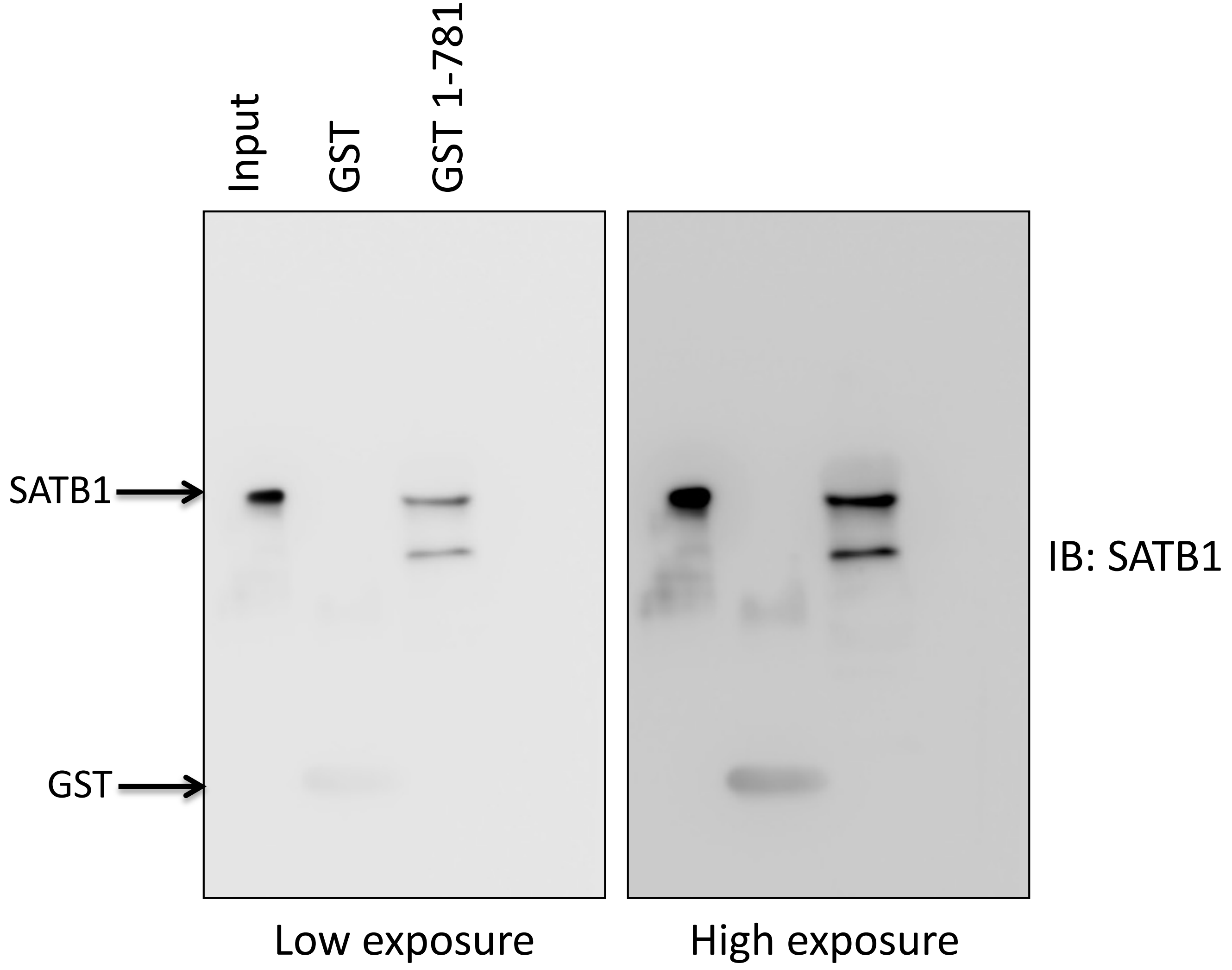

Pull down assay to monitor domain specific interactions (Fig. 1D)

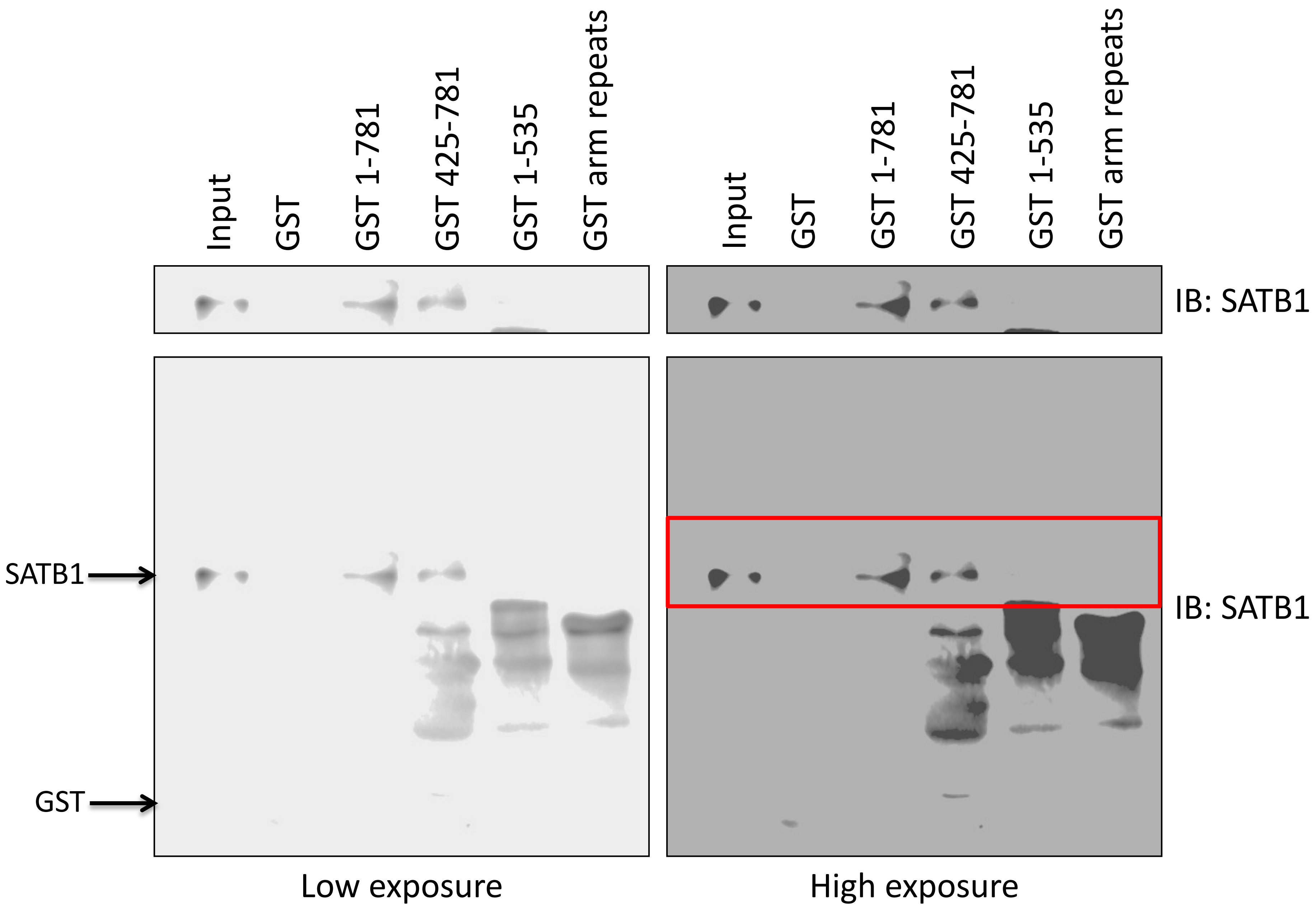

Supplement: S2 File — (ZIP) [file pbio.3001908.s002.zip › 6557773 Replicate Files/Pulldown_Repeat.pdf]
